# Precision-weighted estimates of neonatal, post-neonatal and child mortality for 640 districts in India, National Family Health Survey 2016

**DOI:** 10.7189/jogh.10.020405

**Published:** 2020-12

**Authors:** Rockli Kim, Lathan Liou, Yun Xu, Rakesh Kumar, George Leckie, Mudit Kapoor, R Venkataramanan, Alok Kumar, William Joe, S V Subramanian

**Affiliations:** 1 Division of Health Policy & Management, College of Health Science, Korea University, Seoul, South Korea; 2 Harvard Center for Population & Development Studies, Cambridge, Massachusetts, USA; 3 Department of Public Health and Primary Care, University of Cambridge, Cambridge, UK; 4 SuperMap Software Co. Ltd, Beijing, China; 5 Tata Trusts, Mumbai, India; 6 Centre for Multilevel Modelling, University of Bristol, UK; 7 Economic and Planning Unit, Indian Statistical Institute (ISI), New Delhi, India; 8 University of Warwick, Coventry, England; 9 Medical Health & Family Welfare Department, Government of Uttar Pradesh, Lucknow, India; 10 Institute of Economic Growth (IEG), University of Delhi Enclave, Delhi, India; 11 Department of Social and Behavioral Sciences, Harvard T.H. Chan School of Public Health, Boston, Massachusetts, USA; 12 National Institution for Transforming India (NITI) Honorary Senior Fellow, Government of India, New Delhi, India

## Abstract

**Background:**

The conventional indicators of infant and under-five mortality are aggregate deaths occurring in the first year and the first five years, respectively. Monitoring deaths by <1 month (neonatal), 1-11 months (post-neonatal), and 12-59 months (child) can be more informative given various etiological causes that may require different interventions across these three mutually exclusive periods. For optimal resource allocation, it is also necessary to track progress in robust estimates of child survival at a smaller geographic and administrative level.

**Methods:**

Data on 259 627 children came from the 2015-2016 Indian National Family Health Survey. We used a random effects model to account for the complex survey design and sampling variability, and predicted district-specific probabilities of neonatal, post-neonatal, and child mortality. The resulting precision-weighted estimates are more reliable as they pool information and borrow strength from other districts that share the same state membership. The Pearson correlation and Spearman’s rank correlation were assessed for the three mortality estimates, and the Moran’s I measure was used to detect spatial clustering of high burden districts for each outcome.

**Results:**

The majority of under-five deaths was disproportionately concentrated in the neonatal period. Across all districts, the predicted probability of neonatal, post-neonatal, and child mortality varied from 6.0 to 63.9 deaths, 3.8 to 47.6 deaths, and 1.7 to 11.8 deaths per 1000 live births, respectively. The overall correlation between district-wide probabilities of mortality for the three mutually exclusive periods was moderate (Pearson correlation = 0.47-0.58, Spearman’s rank correlation = 0.58-0.64). For each outcome, a relatively strong spatial clustering was detected across districts that transcended state boundaries (Moran’s I = 0.61-0.76).

**Conclusions:**

Sufficiently breaking down the under-five mortality to distinct age groups and using the precision-weighted estimations to monitor performances at smaller geographic and administrative units can inform more targeted interventions and foster accountability to improve child survival.

Under-5 mortality (U5M) in India has been consistently falling from 125.9 deaths per 1000 live births in 1990 to 45.2 deaths in 2015 [1]. Yet, India has failed to reach the Millennium Development Goal (MDG) to reduce U5M to 42 deaths per 1000 live births by 2015 (MDG 4) [2]. The declaration of the Sustainable Development Goals (SDG) renewed the global commitment to reduce U5M to 25 per 1000 live births and neonatal mortality (NM) rate to 12 per 1000 live births by 2030. The Indian government has also extended the National Health Mission to 2020 and emphasized a more comprehensive approach to primary health care by expanding its Universal Immunization Program (UIP) and strengthening its Health and Wellness Centers (HWC) among other targets [3].

Ongoing policies and research concerning mortality in early childhood routinely monitor and report indicators of NM, infant mortality (IM), and U5M (**Box 1**). On the other hand, postneonatal mortality (PNM, ie, deaths occurring after the first month but before the first year) and childhood mortality (CM, ie, deaths occurring after the first year but before age five) are rarely assessed separately and instead get summed up in the conventional indicators of IM and U5M. While many underlying risks for mortality are continuous and accumulated over time, there may also be etiological causes that are more acute and concentrated at specific age periods [6-9]. Hence, disaggregating U5M into deaths occurring in the mutually exclusive periods of NM, PNM, and CM gives opportunities to further understand potentially distinctive determinants of mortality that require different interventions [10]. Aggregated indicators like U5M can be less meaningful for the purpose of optimizing resource allocation because policy implications for areas with high burden of PNM may likely differ from those with higher burden of CM. There is a need to explicitly distinguish the differences among the different mortality indicators and revisit the importance of under-utilized indicators like PNM and CM (**Box 1**).

Box 1 **Definitions and use of mortality indicators.**

*Neonatal mortality (NM).* The probability of dying within the first 28 days since birth. SDG targets to reduce NM to as low as 12 per 1000 live births by 2030. NM is routinely monitored and reported. In India, 53% of U5M occur in the neonatal period [4] and the daily risk of NM is known to be roughly thirty times higher than the post-neonatal period [5].

*Postneonatal mortality (PNM).* The probability of dying between the 28 days since birth and the first birthday. PNM is often summed up with NM in estimation for IM, and is rarely reported separately.

*Infant mortality (IM).* The probability of dying between birth and the first birthday. IM is routinely monitored and reported.

*Childhood mortality (CM).* The probability of dying between the first and fifth birthdays. CM is often summed up with IM in estimation for U5M, and is rarely reported separately.

*Under-five mortality (U5M).* The probability of dying between birth and the fifth birthday. SDG targets to reduce U5M to as low as 25 per 1000 live births by 2030. U5M is routinely monitored and reported.

Moreover, given the enormous variation in the geographic, socioeconomic, and health profile within India, it is likely that different parts of the country will experience differential rates of improvement in child survival. Hence, monitoring the status of mortality at the national or state level may conceal important heterogeneity occurring at local levels [11-14]. Instead, evaluating the progress (or its lack thereof) in districts – the lowest administrative unit at which infrastructural, developmental, and other services are planned and where demographic data are consistently provided [15,16] – may allow interventions and programmes to be targeted with greater precision. There are 640 districts in India as per the 2011 Census. In order to produce robust district-wise estimations based on survey data, reliability and sampling variability resulting from the survey must be incorporated. While such precision-weighted estimations based on pooling data and borrowing strength is well-established for small area estimation [17,18], they have not been readily applied in global health research and certainly not in prior studies focused on district level mortality rates in India [11,19-23].[REMOVED HYPERLINK FIELD]

In this study, we derive precision-weighted estimates of NM, PNM, and CM for 640 districts in India using data from the latest nationally representative survey. While national and state-specific NM, PNM, and CM estimates have been reported previously [24], these estimates were not precision-weighted nor broken down by districts. The geographic distribution of mortality across these three disaggregated and mutually exclusive life periods are further visualized in maps to aid identification of spatial clustering of districts with high burden.

## MATERIALS AND METHODS

### Data source and study population

The 4th round of the National Family Health Survey (NFHS-4) from 2015-16 were downloaded from https://www.dhsprogram.com/Data/. The NFHS-4 employed a stratified two-stage sampling frame to select a nationally representative sample of households. Using the 2011 Census as the sampling frame, 28 586 primary sampling units (PSUs) were selected with probability proportional to the PSU size. PSUs are equivalent to villages in rural areas and census enumeration blocks in urban areas. In each PSU, a complete household listing was conducted and a fixed proportion of households were selected using systematic sampling [24]. From these households, data on birth and death of all children born within the past 5 years from the survey year were collected. We specifically used Children’s Data (KR) containing one record for every child of interviewed women, born in the five years preceding the survey. The survey provides complete information on age and sex, and age of death (for those who were not alive at the time of the survey) for a total of 259 627 children nested within 640 districts and 36 states/union territories. The age of death was reported by the mother, and thus there is a potential for reporting bias dependent on factors such as the selective omission of births that did not survive, displacement of birth dates, misreporting of the child’s age at death. 52% (N = 135 102) of the children were boys and the average age among 244 508 alive children was 29.7 months (standard deviation [SD] = 17.2).

### Outcomes

In recognition that U5M occurring at different age periods is driven by distinct etiology and hence is more informative when disaggregated [6-9,25], we assessed three mutually exclusive mortality outcomes of NM, PNM, and CM (**Box 1**).

### Analysis

The final analytic sample included 259 627 children at level-1 (*i*) nested within 640 districts at level-2 (*j*) and 36 states at level-3 (*k*). For each of the three mortality outcomes *Y*, a random effects logit model was specified as: logit(*π_ijk_*) = *β_0_* + *v_k_* + *u_jk_*. A random effects model, also known as multilevel model, mixed model, or a variance components model, provides a technically robust and efficient framework to account for the complex survey design and produce precision-weighted estimates for predictions at higher level entities [18,26].

For interpretation, *β_0_* represents the median log odds of mortality across all India; *v_k_* represents the random effect associated with each state *k* (ie, state-specific residual representing a differential from the national median); and *u_jk_* represents the random effect associated with each district *j* (ie, district-specific residual representing a differential from the state median). Assuming a normal distribution with a mean of 0, this model estimates between-state variation as *v_k_* *~* *N* (0,*σ_u_^2^*) and between-district variation as* u_j_* *~* *N* (0,*σ_u_^2^*).

In this model, district-specific predictions can be made by ‘shrunken’ higher level residuals that take into account the ratio of the between-state (and between-district) variance to the total variance, which includes the within-state (and within-district) sampling variance attributable to the sample size of districts within states (and children within districts) [18,26]. Hence, more shrinkage occurs (ie, district-specific means pulled towards the state-specific means) if there are fewer children within districts, and consequently higher sampling variances, and/or when the estimated variance of the districts is small [18,26].

We report the predicted estimates in probability of death multiplied by 1000 for ease of comparability with other literature reporting rates in number of deaths per 1000 live births. We further classified districts into quintiles according to their district-specific mortality risks for each outcome and mapped them using ArcGIS (Esri, Redlands, California, USA). The low mortality quintiles were represented by shades of blue whereas high mortality quintiles were represented by shades of red. Grey represented the third middle quintile. The Pearson correlation and Spearman’s rank correlation were assessed for the three mortality estimates. Lastly, Moran’s I measure of spatial autocorrelation was estimated to assess the degree of clustering among districts with high burden of mortality [27].

Multilevel modeling was performed in the MLwiN 3.03 software program [28] via Markov Chain Monte Carlo (MCMC) methods using Gibbs sampler with non-informative priors, a burn-in of 500 cycles, and monitoring of 5000 iterations of chains [29]. The chains of the loading estimates for all parameters were checked for convergence [29]. We called MLwiN from within Stata (StataCorp, College Station, TX, USA) using the runmlwin command [30].

### Ethics statement

The study was reviewed by Harvard T.H. Chan School of Public Health Institutional Review Board and was considered exempt from full review because the study was based on an anonymous public use data set with no identifiable information on the study participants.

## RESULTS

**Figure 1:**
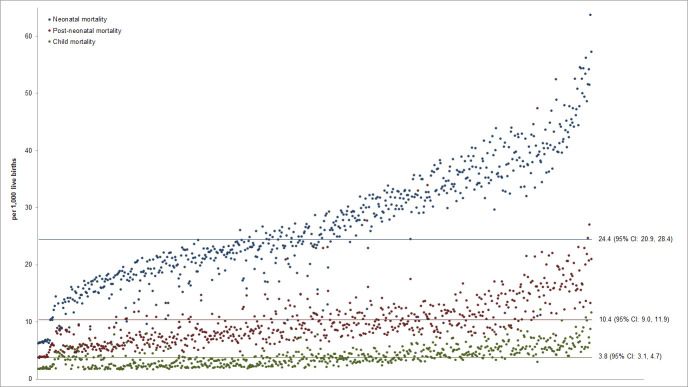
Distribution of precision-weighted probability of neonatal, post-neonatal, and child mortality across 640 districts in India, NFHS 4. The precision-weighted estimates for each district are reported in Table S1 of the **Online Supplementary Document**.

The overall mean probability of NM, PNM, and CM in India, based on our random effects models, were 24.4 deaths per 1000 live births (95% CI = 20.9, 28.4), 10.4 (95% CI = 9.0, 11.9), and 3.8 (95% CI = 3.1, 4.7), respectively (**Figure 1**). 73.6% of the districts (N = 471) had a NM probability that was significantly different from the nationwide mean (*P* < 0.05). Similarly, the precision-weighted PNM and CM for 74.1% (N = 474) and 64.1% (N = 410) of the districts significantly differed from the nationwide means (*P* < 0.05). The predicted NM, PNM, and CM for each district are presented in the Table S1 in the **Online Supplementary Document**. Based on the sum of our precision-weighted NM, PNM, and CM, so far only 75 districts have realized the SDG3 goal of reducing the total U5M to less than 25 deaths per 1000 births (Table S1 in the **Online Supplementary Document**). Estimates stratified by child’s sex (boys vs girls) and place of residence (urban vs rural) are presented in Table S2 in the **Online Supplementary Document**.

### Neonatal mortality

Across all districts, the predicted probability of NM ranged from 6.0 to 63.9 deaths per 1000 live births (**Table 1**). When examined by states, the largest inter-district variation was found within Uttar Pradesh with 71 districts and probability of NM ranging from 33.0 to 63.9 deaths per 1000 live births, followed by Madhya Pradesh with district-wise NM ranging from 25.5 to 44.4 deaths per 1000 live births. The smallest inter-district variation was found within Andaman and Nicobar Islands where the probability of NM was about 10 deaths per 1000 live births across all 3 districts. The quintile cut-points for district NM were 17.7, 22.7, 29.1, and 36.1 deaths per 1000 live births (**Table 2**). Within Uttar Pradesh and Chhattisgarh, two states known for the highest NM rates, 91.6% (N = 65) and 100% (N = 18) of the districts had the highest quintile of NM, respectively, with Gonda (63.9; 95% CI = 45.3 to 89.3), Sitapur (57.0; 95% CI = 39.6 to 81.5), and Hardoi (55.9; 95% CI = 38.9 to 79.6) in Uttar Pradesh and Dakshin Bastar Dantewada (54.3; 95% CI = 36.1 to 80.8) and Uttar Bastar Kanker (52.4; 95% CI = 34.7 to 78.3) in Chhattisgarh being the worst-performing districts. In other states with NM higher than the national mean, there was a greater distribution of districts across the quintiles. For instance, in Jharkhand, 12.5% (N = 3) of the districts fell in the fifth quintile, 58.3% (N = 14) in the fourth quintile, 25.0% (N = 6) in the third quintile, and 4.2% (N = 1) in the second quintile. A total of 41 districts, mainly concentrated in Andaman and Nicobar Islands, Arunachal Pradesh, Kerala, and Puducherry, have met the SDG3 goal of reducing NM to 12 deaths per 1000 live births (Table S1 in the **Online Supplementary Document**).

**Table 1:** Number of live births and deaths, and the range of precision-weighted probability of neonatal, post-neonatal, and child mortality (deaths per 1,000 live births) across districts by 36 states and union territories, NFHS 4

State/UTs	Number of districts	Number live births	Neonatal mortality		Post-neonatal mortality		Child mortality	
**# deaths**	**Lowest, highest probability range**	**# Deaths**	**Lowest, highest probability range**	**# deaths**	**Lowest, highest probability range**
Andaman and Nicobar Islands	3	644	4	10.2, 10.4	2	5.5, 6.1	0	2.1, 2.2
Andhra Pradesh	13	3128	78	19.5, 29.5	27	6.7, 10.8	6	2.1, 2.7
Arunachal Pradesh	16	4966	45	8.2, 13.2	50	7.7, 13.1	32	4.0, 7.7
Assam	27	10309	336	24.0, 41.9	135	8.4, 21.4	47	3.2, 6.1
Bihar	38	25437	941	28.4, 48.1	265	7.7, 16.7	116	3.0, 6.8
Chandigarh	1	194	6	25.3	1	7.5	0	2.6
Chhattisgarh	18	9283	398	36.3, 54.3	92	7.1, 17.6	49	3.3, 8.9
Dadra and Nagar Haveli	1	322	3	13.8	7	14.7	1	3.0
Daman and Diu	2	407	9	19.0, 22.4	3	7.4, 8.6	0	2.3, 2.4
Goa	2	416	6	15.3, 16.5	0	5.2, 5.3	0	2.3, 2.4
Gujarat	26	7730	202	20.7, 33.7	61	6.1, 11.4	31	2.9, 5.6
Haryana	21	7882	179	17.2, 32.4	81	6.8, 14.9	32	2.6, 8.5
Himachal Pradesh	12	2929	71	19.0, 30.4	26	6.8, 11.8	6	2.1, 2.8
Jammu And Kashmir	22	8245	192	18.3, 24.9	75	6.4, 11.7	28	2.6, 3.9
Jharkhand	24	12204	386	22.8, 39.2	117	6.7, 15.5	67	3.5, 8.8
Karnataka	30	7789	161	17.0, 26.8	65	6.3, 13.9	20	2.2, 3.3
Kerala	14	2462	11	6.0, 6.8	6	3.8, 5.1	2	1.7, 2.0
Lakshadweep	1	308	7	21.2	1	6.3	0	2.4
Madhya Pradesh	50	24611	874	25.5, 44.4	326	8.9, 22.5	161	3.7, 11.8
Maharashtra	35	9401	175	15.7, 22.2	56	5.1, 8.5	16	1.7, 2.6
Manipur	9	5636	94	14.1, 18.3	31	4.8, 7.0	13	1.9, 3.7
Meghalaya	7	4409	77	12.9, 21.3	56	8.5, 15.5	23	3.5, 7.1
Mizoram	8	4905	55	10.4, 13.2	158	21.1, 47.6	18	2.7, 4.6
Nagaland	11	4607	81	12.4, 22.6	62	9.9, 16.0	24	3.6, 7.6
Delhi	9	1580	27	15.3, 19.3	21	8.9, 14.6	8	3.3, 7.0
Odisha	30	11106	345	22.7, 38.4	121	7.5, 21.5	45	2.9, 5.6
Puducherry	4	1081	10	10.5, 11.7	7	6.0, 8.9	3	2.5, 3.8
Punjab	20	5216	120	19.3, 26.4	42	6.3, 12.6	10	1.9, 2.7
Rajasthan	33	16832	499	23.2, 39.0	180	6.1, 17.7	79	3.1, 8.2
Sikkim	4	1005	20	16.9, 26.8	9	7.2, 11.6	1	2.2, 2.3
Tamil Nadu	32	7922	116	12.5, 17.8	42	4.6, 8.4	36	3.3, 6.1
Tripura	4	1330	18	13.0, 16.5	18	8.8, 14.7	3	2.4, 2.9
Uttar Pradesh	71	41751	1827	33.0, 63.9	730	10.0, 26.9	273	4.0, 11.5
Uttarakhand	13	5825	160	22.4, 31.2	68	8.4, 14.1	17	2.3, 3.2
West Bengal	19	5328	125	20.3, 26.6	28	4.6, 7.1	12	2.0, 3.5
Telangana	10	2427	57	18.3, 27.1	18	6.1, 9.7	3	1.9, 2.2

**Table 2:** Distribution (number (%)) of districts by quintiles of precision-weighted probability of neonatal, post-neonatal, and child mortality by 36 states and union territories, NFHS 4

State	Neonatal mortality					Post-neonatal mortality					Child mortality				
	**Q1**	**Q2**	**Q3**	**Q4**	**Q5**	**Q1**	**Q2**	**Q3**	**Q4**	**Q5**	**Q1**	**Q2**	**Q3**	**Q4**	**Q5**
Andaman and Nicobar Islands	3 (100%)					3 (100%)					3 (100%)				
Andhra Pradesh		4 (30.8%)	8 (61.5%)	1 (7.7%)			8 (61.5%)	3 (23.1%)	2 (15.4%)		8 (61.5%)	5 (38.5%)			
Arunachal Pradesh	16 (100%)						5 (31.3%)	6 (37.5%)	4 (25.0%)	1 (6.3%)			1 (6.3%)	7 (43.8%)	8 (50.0%)
Assam			7 (25.9%)	15 (55.6%)	5 (18.5%)			6 (22.2%)	14 (51.9%)	7 (25.9%)		3 (11.1%)	11 (40.7%)	9 (33.3%)	4 (14.8%)
Bihar			1 (2.6%)	21 (55.3%)	16 (42.1%)		5 (13.2%)	18 (47.4%)	12 (31.6%)	3 (7.9%)		4 (10.5%)	16 (42.1%)	13 (34.2%)	5 (13.2%)
Chandigarh			1 (100%)				1 (100%)					1 (100%)			
Chhattisgarh					18 (100%)		9 (50.0%)	1 (5.6%)	7 (38.9%)	1 (5.6%)			2 (11.1%)	12 (66.7%)	4 (22.2%)
Dadra and Nagar Haveli	1 (100%)									1 (100%)		1 (100%)			
Daman and Diu		1 (50.0%)	1 (50.0%)				1 (50.0%)	1 (50.0%)				2 (100%)			
Goa	2 (100%)					2 (100%)					1 (50.0%)	1 (50.0%)			
Gujarat		7 (26.9%)	14 (53.9%)	5 (19.2%)		8 (30.8%)	13 (50.0%)	2 (7.7%)	3 (11.5%)			9 (34.6%)	12 (46.2%)	3 (11.5%)	2 (7.7%)
Haryana	3 (14.3%)	12 (57.1%)	5 (23.8%)	1 (4.8%)			7 (33.3%)	7 (33.3%)	6 (28.6%)	1 (4.8%)		15 (71.4%)	4 (19.1%)	1 (4.8%)	1 (4.8%)
Himachal Pradesh		6 (50.0%)	5 (41.7%)	1 (8.3%)			6 (50.0%)	5 (41.7%)	1 (8.3%)		7 (58.3%)	5 (41.7%)			
Jammu And Kashmir		9 (40.9%)	13 (59.1%)			2 (9.1%)	8 (36.4%)	8 (36.4%)	4 (18.2%)			16 (72.7%)	6 (27.3%)		
Jharkhand		1 (4.2%)	6 (25.0%)	14 (58.3%)	3 (12.5%)	1 (4.2%)	5 (20.8%)	13 (54.2%)	4 (16.7%)	1 (4.2%)			3 (12.5%)	14 (58.3%)	7 (29.2%)
Karnataka	2 (6.7%)	25 (83.3%)	3 (10.0%)			2 (6.7%)	21 (70.0%)	6 (20.0%)		1 (3.3%)	9 (30.0%)	20 (66.7%)	1 (3.3%)		
Kerala	14 (100%)					14 (100%)					14 (100%)				
Lakshadweep		1 (100%)				1 (100%)					1 (100%)				
Madhya Pradesh			2 (4.0%)	32 (64.0%)	16 (32.0%)			9 (18.0%)	18 (36.0%)	23 (46.0%)			1 (2.0%)	16 (32.0%)	33 (66.0%)
Maharashtra	17 (48.6%)	18 (51.4%)				32 (91.4%)	2 (5.7%)	1 (2.9%)			34 (97.1%)	1 (2.9%)			
Manipur	6 (66.7%)	3 (33.3%)				8 (88.9%)	1 (11.1%)				6 (66.7%)	2 (22.2%)	1 (11.1%)		
Meghalaya	3 (42.9%)	4 (57.1%)						1 (14.3%)	4 (57.1%)	2 (28.6%)			3 (42.9%)	3 (42.9%)	1 (14.3%)
Mizoram	8 (100%)									8 (100%)		4 (50.0%)	3 (37.5%)	1 (12.5%)	
Nagaland	6 (54.6%)	4 (36.4%)	1 (9.1%)					2 (18.2%)	5 (45.5%)	4 (36.4%)			4 (36.4%)	5 (45.5%)	2 (18.2%)
Delhi	7 (77.8%)	2 (22.2%)						2 (22.2%)	5 (55.6%)	2 (22.2%)			7 (77.8%)	1 (11.1%)	1 (11.1%)
Odisha			11 (36.7%)	15 (50.0%)	4 (13.3%)		6 (20.0%)	10 (33.3%)	11 (36.7%)	3 (10.0%)		11 (36.7%)	13 (43.3%)	4 (13.3%)	2 (6.7%)
Puducherry	4 (100%)					2 (50.0%)	1 (25.0%)	1 (25.0%)				3 (75.0%)	1 (25.0%)		
Punjab		11 (55.0%)	9 (45.0%)			1 (5.0%)	15 (75.0%)	3 (15.0%)	1 (5.0%)		16 (80.0%)	4 (20.0%)			
Rajasthan			17 (51.5%)	15 (45.5%)	1 (3.0%)	1 (3.0%)	3 (9.1%)	14 (42.4%)	11 (33.3%)	4 (12.1%)		1 (3.0%)	14 (42.4%)	14 (42.4%)	4 (12.1%)
Sikkim	1 (25.0%)	2 (50.0%)	1 (25.0%)				3 (75.0%)		1 (25.0%)		3 (75.0%)	1 (25.0%)			
Tamil Nadu	31 (96.9%)	1 (3.1%)				31 (96.9%)	1 (3.1%)					1 (3.1%)	21 (65.6%)	6 (18.8%)	4 (12.5%)
Tripura	4 (100%)							1 (25.0%)	2 (50.0%)	1 (25.0%)		4 (100%)			
Uttar Pradesh				6 (8.5%)	65 (91.6%)			1 (1.4%)	8 (11.3%)	62 (87.3%)			2 (2.8%)	19 (26.8%)	50 (70.4%)
Uttarakhand		2 (15.4%)	9 (69.2%)	2 (15.4%)				5 (38.5%)	5 (38.5%)	3 (23.1%)	1 (7.7%)	11 (84.6%)	1 (7.7%)		
West Bengal		11 (57.9%)	8 (42.1%)			18 (94.7%)	1 (5.3%)				15 (79.0%)	3 (15.8%)	1 (5.3%)		
Telangana		4 (40.0%)	6 (60.0%)			2 (20.0%)	6 (60.0%)	2 (20.0%)			10 (100%)				

*The quintile cut-points were 17.7, 22.7, 29.1, and 36.1 deaths per 1000 live births for neonatal mortality; 6.7, 8.3, 10.1, and 12.9 deaths per 1000 live births for post-neonatal mortality; and 2.3, 3.3, 4.0, and 5.2 deaths per 1000 live births for child mortality.

### Post-neonatal mortality

The predicted probability of PNM ranged from 3.8 to 47.6 deaths per 1000 live births across all districts in India (**Table 1**), and the largest variation was found in Mizoram (range: 21.1-47.6) followed by Uttar Pradesh (range: 10.0-26.9). The quintile cut-points for district PNM were 6.7, 8.3, 10.1, and 12.9 deaths per 1000 live births (**Table 2**). While all 8 districts within Mizoram had the lowest NM quintile, they all fell in the highest quintile for PNM. In fact, the top four worst performing districts in India in terms of PNM were located in Mizoram: Saiha (47.6; 95% CI = 29.4 to 76.2), Lunglei (33.5; 95% CI = 19.8 to 56.2), Kolasib (33.0; 95% CI = 19.9 to 54.4), and Lawngtlai (27.5; 95% CI = 15.4 to 48.8). Within Uttar Pradesh, 87.3% (N = 62) of the districts fell in the highest quintile, with Farrukhabad (26.9; 95% CI = 16.7 to 43.0), Kanshiram Nagar (24.4; 95% CI = 15.2 to 38.9) and Kannauj (23.1; 95% CI = 14.2 to 37.3) having a notably high PNM, and the remaining 11.3% (N = 8) and 1.4% (N = 1) districts fell in the fourth and third quintile, respectively. In states known for low PNM rates (ie, Kerala, Lakshadweep, Andaman & Nicobar Islands), all districts had the lowest quintile. In Rajasthan, a state with PNM close to the national average, the districts were distributed across all quintiles from the highest to the lowest by 12.1% (N = 4), 33.3% (N = 11), 42.4% (N = 14), 9.1% (N = 3), and 3.0% (N = 1).

### Child mortality

Across all districts, the precision-weighted probability of CM ranged from 1.7 to 11.8 deaths per 1000 live births (**Table 1**). In Madhya Pradesh and Uttar Pradesh, the CM ranged from 3.7 to 11.8 deaths and 4.0 to 11.5 deaths, respectively. The variability was also large in Haryana (range: 2.6-8.5). The difference between the lowest and the highest probability of CM was less than 1 death per 1000 live births in districts within Daman and Diu, Goa, Andaman and Nicobar Islands, Sikkim, Kerala, Telangana, Tripura, Andhra Pradesh, Himachal Pradesh, Punjab, Maharashtra, Uttarakhand. Districts were classified into quintiles based on the cut-points of 2.3, 3.3, 4.0, and 5.2 deaths per 1000 live births (**Table 2**). In Uttar Pradesh and Madhya Pradesh, almost 70% of the districts ranked within the highest quintile. In Andaman and Nicobar Islands, Maharashtra, Punjab, Sikkim, West Bengal and Telangana, 70%-100% of the districts fell within the lowest quintile.

### Mapping the geographical distribution of mortality

**Figure 2:**
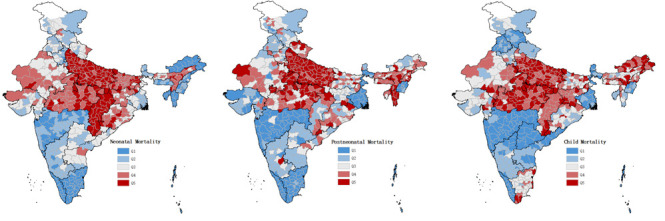
Maps illustrating the precision-weighted probability of neonatal, post-neonatal, and child mortality (in quintiles) across 640 districts in India, NFHS 4. The quintile cutpoints were 17.7, 22.7, 29.1, and 36.1 deaths per 1000 live births for neonatal mortality; 6.7, 8.3, 10.1, and 12.9 deaths per 1000 live births for post-neonatal mortality; and 2.3, 3.3, 4.0, and 5.2 deaths per 1000 live births for child mortality. State index map provided in Figure S1 of the **Online Supplementary Document**.

**Figure 2** illustrates that while some districts have consistently high (or low) probability of mortality across all age groups, other districts show inconsistency. In Chhattisgarh, all districts had the highest quintile of NM and the majority (67%) fell in the fourth quintile of CM, but 50% were doing fairly well in terms of PNM (second quintile). In Tamil Nadu and Arunachal Pradesh, almost all of the districts had the lowest level of probability for NM, but had higher probabilities for PNM and CM. The overall correlation between district-wide probabilities of NM, PNM, and CM were moderate (Pearson correlation = 0.47-0.58, Spearman’s rank correlation = 0.58-0.64) (**Table 3**). In addition, a relatively strong spatial clustering was detected across districts that transcended state boundaries (Moran’s I = 0.61-0.76), with large patches of spatially contiguous districts from the states of Uttar Pradesh, Bihar, Madhya Pradesh, and Chhattisgarh with high burden of mortality (Figure S2 in the **Online Supplementary Document**).

**Table 3:** Correlation statistics for the precision-weighted probability of neonatal, post-neonatal, and child mortality across 640 districts in India, NFHS 4

	Neonatal mortality	Post-neonatal mortality	Child mortality
**Pearson correlation:**			
Neonatal mortality	1		
Post-neonatal mortality	0.475*	1	
Child mortality	0.581*	0.514*	1
**Spearman's rank correlation:**			
Neonatal mortality	1		
Post-neonatal mortality	0.595*	1	
Child mortality	0.581*	0.638*	1
**Moran' s I bi-variate spatial correlation:**			
Neonatal mortality	0.764*		
Post-neonatal mortality	0.414*	0.675*	
Child mortality	0.478*	0.421*	0.614*

*P < 0.0001.

## DISCUSSION

We present four salient findings from the precision-weighted estimates of NM, PNM, and CM for all 640 districts in India. First, we found the majority of U5M was concentrated in the neonatal period followed by the post-neonatal age group and a substantially lower probability of CM. Aggregating deaths from these three distinct age groups into the conventional indicators of IM or U5M masks the greater investment needed in health care quality to improve child survival for distinct age groups. Second, we revealed substantial variability in the probability of mortality across districts that are overlooked in summaries based on national and state-wide averages. The precision-weighted estimations accounting for sampling variability could thus be used to monitor and identify performances at smaller geographic and administrative units to motivate accountability and targeted interventions. Third, the overall correlation between district-wide probabilities of NM, PNM, and CM was moderate, meaning that districts that had high probability of NM did not necessarily have high PNM or CM, and vice versa. Fourth, strong regional spatial patterning in mortality estimates was detected suggesting opportunities for districts from different states to collaborate to achieve the common goals of reducing NM, PNM, and CM. Taken together, we found that many districts have distinct age-specific mortality challenges, indicating that state-level mortality patterns are too non-specific to make effective targeted policy changes.

It is important to note that while the NFHS applied the synthetic cohort life table approach to estimate mortality rates based on the number of deaths over the number of births in a given time period [24], we have estimated the probability of death per 1000 live births. Compared to the mortality rates reported by the NFHS (ie, 29.5 for NM, 11.3 for PNM, and 9.4 for CM) [24], our probability estimates were more conservative due to precision-weighting, such that districts with smaller sample sizes and unreliable estimates were down-weighted more towards the overall mean [18,26], and the largest difference was seen for CM. The required sample size needed to achieve unbiased estimates from the synthetic cohort approach ranges from at least 500 [31] to thousands of observations [32]. Similarly, we present more reliable and appropriately conservative estimates compared to a recent study [23] that has also used the synthetic cohort probability approach to estimate district-wide NM. However, we acknowledge that a future validation study should be performed comparing the estimates of different estimation methods by, for instance, quantifying their coefficients of variance. Nevertheless, many of the districts that were identified to have the highest NM in their study [23] aligned with our findings (Gonda, Sitapur, Budaun, Dakshin Bastar Dantewada, Kaushambi, Shahjahanpur), which were also consistent with the priority districts identified in the Ministry of Health and Family Welfare’s annual report on health [25].

While we provide additional stratifications by child’s sex and place of residence (urban vs rural) **(**Table S2 in the **Online Supplementary Document****)** as resources for health planning, our study still lacks stratified estimates by socioeconomic status and smaller geographic areas within districts. In India, NM was shown to be greater for boys while PNM and CM were higher for girls, and the difference has increased over time for the former while the opposite was found for the latter [33]. A negative wealth gradient in IM and U5M has been well documented elsewhere [13,14,33]. While substantial variation in mortality is expected within districts [34], it is not possible to obtain village level estimates in the absence of complete registration of births and deaths in India. Lastly, information on children’s birth date, survival status, and age of death if a child died were self-reported by mothers, so there exists the potential for underreporting bias in particular. However, the DHS is known for its high response rates, national coverage, high quality interviewer training, standardized data collection procedures across countries that are consistent over time [35].

Despite these potential limitations, our findings have important implications to renew the policy discussion in India for more efficient prioritization and resource allocation to reduce U5M. Historically, decentralized planning in India through the *panchayat* system allowed to create health action plans, train health professionals, deliver resources, and monitor facilities either with or without state involvement. While different districts had varying levels of success, a case study showed that district-led health planning with state-level oversight was able to achieve the majority of its stated goals, such as providing antenatal care kits to midwives and establishing Mother and Child Protection antenatal service camps with proper staffing and facility maintenance [36]. For instance, different components of Integrated Child Development Services (ICDS) may be emphasized more depending on the share of NM, PNM, and CM within each district. In India, 78% of neonatal deaths were attributed to prematurity, neonatal infections, and birth asphyxia [37]. Previously, birth asphyxia and neonatal infection rates were found to be the highest in central India, in states such as Madhya Pradesh and Chhattisgarh, and prematurity and low birthweight were found to be the highest in West India, in states such as Rajasthan and Gujarat. Given our district-level estimates, areas with the highest probability of NM may be targeted for improvement in delivery care to ensure that all births occur in health centres with comprehensive facilities, such as Special New Born Care Units (SNCUs), Newborn Stabilization Units (NBSUs), and Newborn Care Corners (NBCCs), to provide sterile and proper neonatal care [25]. Investments in improved diet and nutrition of mothers, such as with iron tablet supplementation, may also be further emphasized in these areas [38].

Districts that do not suffer from high probability of NM may suffer from high PNM or CM. Since pneumonia and diarrhea are common causes of deaths after the first month but before the first year [37], districts with higher burden of PNM may prioritize introduction of vaccines as well as education on sanitation practices (eg, hand-washing and safe drinking water) for prevention of diarrhea and access to oral rehydration solution (ORS)-zinc tablet treatments [25,39]. To reduce CM, our estimates suggest that nutrition interventions [40] and UIP should be intensified in not only districts in Central India, but also select districts in Tamil Nadu, Arunachal Pradesh, Assam, Meghalaya, and Nagaland, which we have identified as among the highest mortality quintile in CM. The accredited social health activities (ASHA) program should continue to grow in the worst-performing districts via their bi-annual vitamin A supplements and bi-weekly iron supplements program to combat poor nutrition [25].

## CONCLUSION

By disaggregating U5M into NM, PNM, and CM, we highlight distinct distributions of age-specific mortality across 640 districts in India. Our precision-weighted estimates may serve as evidence to assist district administration develop appropriate interventions and programmes to prioritize the most precarious age groups within their district. To reduce U5M as a whole, focusing more attention on neonatal period may be most effective and efficient. Efforts to improve nutritional status of the population should adopt a life cycle perspective since interventions targeting adolescent population can go a long way in improving the nutritional status of the reproductive age group as well as birth outcomes. Further etiological studies on the causative factors of mortality in these three periods of early childhood are needed to develop more specific treatments and prevention programmes.

## Additional material

Online Supplementary Document
