# Supplementary material for: Precision-weighted estimates of neonatal, post-neonatal and child mortality for 640 districts in India, National Family Health Survey 2016
Source: J Glob Health. 2020 Aug 11;10(2):020405. doi: 10.7189/jogh.10.020405 (PMC7568918; doi:10.7189/jogh.10.020405)

Precision-Weighted Estimates of Neonatal, Post-Neonatal and Child Mortality for 640 Districts in India, National Family Health Survey 2016

Online Supplementary Documents

Table S1. District-specific data including the precision-weighted probability of neonatal mortality (NM), post-neonatal mortality (PNM), and child mortality (CM), NFHS 4

State name	District Name	Raw Number				Precision-weighted probabilities (per 1,000 live births)									SDG (1=achieved already)	
		Live births	NM	PNM	CM	NM	95 CI		PNM	95 CI		CM	95 CI		NM<12	Under -5<25
Andaman And Nicobar Islands	Nicobars	247	2	1	0	10.4	4.9	21.8	5.9	2.4	14.6	2.2	0.7	6.4	1	1
Andaman And Nicobar Islands	North & Middle Andaman	200	1	0	0	10.2	4.8	21.6	5.5	2.1	14.2	2.2	0.7	6.5	1	1
Andaman And Nicobar Islands	South Andaman	197	1	1	0	10.2	4.8	21.4	6.1	2.5	15.2	2.1	0.7	6.2	1	1
Andhra Pradesh	Srikakulam	240	11	1	0	28.6	17.3	46.9	7.6	3.6	15.6	2.1	0.8	5.6	0	0
Andhra Pradesh	Vizianagaram	254	4	2	0	22	13.2	36.5	8.2	4	16.8	2.1	0.8	5.4	0	0
Andhra Pradesh	Visakhapatnam	187	4	2	0	23.4	13.9	39.2	8.6	4.1	18	2.1	0.8	5.6	0	0
Andhra Pradesh	East Godavari	225	5	5	0	23.4	14.2	38.4	10.8	5.2	22.3	2.1	0.8	5.5	0	0
Andhra Pradesh	West Godavari	181	4	1	0	23.5	14.1	38.8	7.9	3.8	16.5	2.2	0.8	5.8	0	0
Andhra Pradesh	Krishna	200	1	2	1	20.5	12.3	34	8.4	4	17.3	2.5	0.9	6.4	0	0
Andhra Pradesh	Guntur	193	3	4	0	22.4	13.6	36.6	10.2	4.8	21.3	2.2	0.8	5.7	0	0
Andhra Pradesh	Prakasam	281	13	4	1	29.5	18.1	47.9	9.4	4.5	19.5	2.4	0.9	6	0	0
Andhra Pradesh	Sri Potti Sriramulu Nellore	256	8	0	0	25.1	15.2	41.2	6.7	3.2	13.9	2.1	0.8	5.6	0	0
Andhra Pradesh	Y.S.R.	273	12	1	0	28.5	17.4	46.4	7.3	3.6	14.7	2.1	0.8	5.3	0	0
Andhra Pradesh	Kurnool	366	8	3	2	23.2	14.3	37.4	8.2	4.1	16.5	2.7	1	7.1	0	0
Andhra Pradesh	Anantapur	242	5	2	1	22.8	13.9	37.3	8.1	4	16.7	2.5	0.9	6.7	0	0
Andhra Pradesh	Chittoor	230	0	0	1	19.5	11.4	33	7	3.4	14.3	2.5	1	6.4	0	0
Arunachal Pradesh	Tawang	244	1	1	2	8.9	5.1	15.3	8	4	16.1	5.7	2.5	13	1	1
Arunachal Pradesh	West Kameng	274	1	2	0	8.8	5.1	15.2	8.7	4.4	17.2	4.3	1.8	10	1	1
Arunachal Pradesh	East Kameng	429	7	9	3	10.6	6.2	18	13.1	6.9	24.9	5.6	2.6	12.2	1	0
Arunachal Pradesh	Papumpare	631	5	5	1	9.1	5.3	15.4	8.6	4.6	16.2	4	1.7	9.1	1	1
Arunachal Pradesh	Upper Subansiri	346	0	2	1	8.2	4.7	14.1	8.3	4.1	16.4	4.7	2	10.7	1	1
Arunachal Pradesh	West Siang	213	0	1	2	8.6	5	14.8	8.4	4.2	16.6	5.8	2.5	13.6	1	1
Arunachal Pradesh	East Siang	203	0	1	0	8.7	5	15	8.3	4.1	16.8	4.6	1.9	10.9	1	1
Arunachal Pradesh	Upper Siang	169	0	0	0	8.8	5	15.3	7.9	3.8	16.3	4.5	1.9	10.9	1	1
Arunachal Pradesh	Changlang	304	3	3	1	9.4	5.5	16.1	9.1	4.6	17.9	4.7	2	11.1	1	1
Arunachal Pradesh	Tirap	319	4	5	2	9.8	5.8	16.7	10.8	5.5	21.1	5.4	2.4	12.2	1	0
Arunachal Pradesh	Lower Subansiri	207	1	0	1	9.1	5.2	16	7.7	3.9	15.4	5.1	2.1	12	1	1
Arunachal Pradesh	Kurung Kumey	447	3	4	6	8.9	5.3	15	9	4.7	17.2	7.5	3.4	16.2	1	0
Arunachal Pradesh	Dibang Valley	222	0	2	3	8.5	4.9	14.8	8.9	4.4	18.1	6.5	2.9	14.7	1	1
Arunachal Pradesh	Lower Dibang Valley	295	3	5	5	9.5	5.5	16.3	11.2	5.6	22.2	7.7	3.4	17.4	1	0
Arunachal Pradesh	Lohit	397	13	6	2	13.2	7.7	22.4	11	5.7	21	5.1	2.3	11.4	0	0
Arunachal Pradesh	Anjaw	266	4	4	3	9.8	5.6	16.9	10.5	5.4	20.4	6.3	2.8	14.2	1	0
Assam	Kokrajhar	345	11	3	2	31.5	20.5	48	10.7	5.7	19.8	4.3	1.9	9.5	0	0
Assam	Dhubri	463	8	5	1	25.9	16.7	39.9	11.4	6.2	20.7	3.6	1.6	7.9	0	0
Assam	Goalpara	457	12	5	2	29.1	19.2	43.9	11.4	6.4	20.2	4.1	1.8	9.1	0	0
Assam	Barpeta	440	12	6	0	29.7	19.4	45.1	12.3	6.7	22.3	3.2	1.4	7.3	0	0
Assam	Morigaon	483	27	16	2	41.9	27.9	62.5	21.4	12.4	36.7	4	1.8	9	0	0
Assam	Nagaon	430	21	5	1	38.1	25.5	56.6	11.5	6.4	20.9	3.6	1.6	8	0	0
Assam	Sonitpur	294	8	4	0	30	19.1	46.9	12	6.4	22.4	3.4	1.5	7.6	0	0
Assam	Lakhimpur	361	9	4	5	29.2	18.9	45.1	11.4	6.1	21.3	6.1	2.8	13.3	0	0
Assam	Dhemaji	432	19	2	1	36.2	24.2	54	9.4	5.1	17.1	3.6	1.6	8.1	0	0
Assam	Tinsukia	362	12	4	4	31.9	20.8	48.7	11.5	6.2	21.1	5.5	2.5	11.9	0	0
Assam	Dibrugarh	318	2	2	2	24	15.1	38.1	10.2	5.5	18.9	4.4	1.9	9.9	0	0
Assam	Sivasagar	312	11	7	1	32.5	21.1	49.7	15.1	8.1	27.9	3.9	1.7	8.9	0	0
Assam	Jorhat	294	8	1	1	29.9	19.1	46.4	9.6	5	18.2	3.8	1.7	8.9	0	0
Assam	Golaghat	319	16	3	1	37.4	24.4	56.9	11	5.9	20.5	3.8	1.7	8.5	0	0
Assam	Karbi Anglong	414	17	8	4	35	23.2	52.4	14.6	8.1	26	5.3	2.4	11.4	0	0
Assam	Dima Hasao	418	14	4	4	32.2	21	49	10.9	6	20	5.1	2.3	11.3	0	0

Assam	Cachar	376	16	10	1	35.1	23	53.3	17.1	9.6	30.3	3.7	1.6	8.3	0	0
Assam	Karimganj	514	23	13	3	37.2	24.5	56.1	17.6	9.9	31.1	4.4	2	9.6	0	0
Assam	Hailakandi	432	18	6	2	35.1	23.1	53.1	12.4	6.8	22.3	4.1	1.8	9.1	0	0
Assam	Bongaigaon	379	5	2	2	25.2	16.1	39.1	9.5	5.1	17.9	4.2	1.8	9.5	0	0
Assam	Chirang	387	9	3	3	28.5	18.6	43.5	10.5	5.6	19.5	4.8	2.2	10.5	0	0
Assam	Kamrup	341	6	0	2	27.1	17.3	42.2	8.4	4.4	16	4.3	1.9	9.5	0	0
Assam	Kamrup Metropolitan	227	8	1	2	32.3	20.6	50.2	10.1	5.3	19.4	4.5	1.9	10.7	0	0
Assam	Nalbari	329	12	7	0	32.9	21.3	50.4	14.9	8.1	27.2	3.3	1.4	7.6	0	0
Assam	Baksa	388	7	6	1	26.9	17.4	41.2	13	7	23.9	3.7	1.7	8.2	0	0
Assam	Darrang	431	14	3	0	31.5	20.7	47.7	9.8	5.1	18.6	3.2	1.4	7.2	0	0
Assam	Udalguri	363	11	5	0	30.9	20	47.2	12.1	6.6	22.2	3.3	1.4	7.8	0	0
Bihar	Pashchim Champaran	602	27	5	7	40.5	27.7	58.9	9.2	5.1	16.5	6.5	3.1	13.4	0	0
Bihar	Purba Champaran	714	20	17	1	31.7	21.4	46.7	16.7	10	27.9	3.2	1.5	6.8	0	0
Bihar	Sheohar	689	30	11	5	40.1	27.7	57.7	12.3	7.1	21.4	5.1	2.5	10.3	0	0
Bihar	Sitamarhi	668	30	6	8	40.6	27.8	58.9	9.3	5.2	16.5	6.8	3.4	13.5	0	0
Bihar	Madhubani	631	20	8	5	33.6	22.8	49.3	10.8	6.1	19.1	5.2	2.6	10.5	0	0
Bihar	Supaul	787	22	6	2	31.6	21.6	46.1	8.7	4.9	15.5	3.6	1.7	7.5	0	0
Bihar	Araria	719	35	5	8	42.9	29.7	61.6	8.6	4.9	15.1	6.5	3.3	12.9	0	0
Bihar	Kishanganj	692	25	5	1	36.1	24.7	52.3	8.5	4.7	15.2	3.2	1.5	6.9	0	0
Bihar	Purnia	743	42	11	8	48.1	33.6	68.5	12.1	7.1	20.7	6.5	3.3	12.9	0	0
Bihar	Katihar	618	30	4	2	42.5	29.4	61	8.5	4.8	15.1	3.8	1.8	7.9	0	0
Bihar	Madhepura	793	29	5	5	36.3	24.7	53	8.3	4.8	14.4	4.8	2.4	9.8	0	0
Bihar	Saharsa	760	38	13	6	44.2	30.8	63	13.2	7.7	22.6	5.4	2.7	10.8	0	0
Bihar	Darbhanga	662	20	4	3	32.8	22.3	48.1	8.2	4.6	14.7	4.2	2.1	8.5	0	0
Bihar	Muzaffarpur	538	19	2	1	35.6	23.9	52.7	7.7	4.2	14	3.5	1.6	7.5	0	0
Bihar	Gopalganj	572	16	6	3	32.1	21.6	47.4	10.1	5.6	18.1	4.3	2.1	9	0	0
Bihar	Siwan	598	24	5	3	38	25.8	55.7	9.2	5.1	16.5	4.3	2	9.1	0	0
Bihar	Saran	588	16	10	0	31.7	21.4	46.9	12.9	7.3	22.5	3	1.4	6.5	0	0
Bihar	Vaishali	687	22	10	3	34	23	50	11.7	6.8	20.2	4.1	2	8.5	0	0
Bihar	Samastipur	640	17	7	1	31.4	21.1	46.3	10.3	5.8	18	3.3	1.6	7	0	0
Bihar	Begusarai	698	21	6	2	32.9	22.4	48.1	9.2	5.2	16.3	3.7	1.8	7.6	0	0
Bihar	Khagaria	753	29	7	2	37.5	25.8	54.2	9.5	5.5	16.5	3.6	1.8	7.5	0	0
Bihar	Bhagalpur	710	15	12	2	28.4	19.1	41.9	12.9	7.6	22	3.7	1.8	7.6	0	0
Bihar	Banka	618	22	6	2	35.8	24.2	52.6	9.7	5.5	16.9	3.9	1.8	8.3	0	0
Bihar	Munger	525	21	3	1	37.6	25.4	55.3	8.4	4.6	15.1	3.5	1.6	7.5	0	0
Bihar	Lakhisarai	675	23	10	2	34.5	23.6	50.2	12.1	7	20.7	3.7	1.7	7.8	0	0
Bihar	Sheikhpura	722	25	7	5	35.1	24	50.9	9.7	5.5	17.2	4.9	2.4	10.1	0	0
Bihar	Nalanda	567	19	8	2	34.6	23.5	50.5	11.4	6.5	20.1	3.9	1.9	8.3	0	0
Bihar	Patna	1108	32	9	2	31.5	21.9	45.1	8.9	5.2	15	3.2	1.6	6.4	0	0
Bihar	Bhojpur	618	19	3	2	33.3	22.6	48.8	7.8	4.4	13.8	3.8	1.8	7.9	0	0
Bihar	Buxar	639	30	5	3	41.7	28.7	60.4	9.1	5	16.5	4.2	2	8.8	0	0
Bihar	Kaimur (Bhabua)	626	23	8	3	36.4	24.7	53.2	11	6.3	19.3	4.2	2.1	8.7	0	0
Bihar	Rohtas	630	30	5	4	42.1	29	60.8	8.9	4.9	16.2	4.6	2.2	9.6	0	0
Bihar	Aurangabad	521	15	7	2	32.5	21.9	48	11.1	6.2	19.9	4	1.8	8.6	0	0
Bihar	Gaya	800	39	7	4	43.3	30.1	61.9	9.3	5.4	16.1	4.4	2.2	8.8	0	0
Bihar	Nawada	595	17	3	1	32.4	21.9	47.5	7.9	4.3	14.4	3.4	1.6	7.4	0	0
Bihar	Jamui	733	34	4	3	41.9	29	60.1	8	4.4	14.3	4.1	1.9	8.5	0	0
Bihar	Jehanabad	549	26	8	1	41.6	28.5	60.5	11.5	6.5	20.3	3.5	1.6	7.5	0	0
Bihar	Arwal	649	19	7	1	32.5	22.1	47.6	10	5.8	17.4	3.3	1.5	7	0	0
Chandigarh	Chandigarh	194	6	1	0	25.3	11.2	56.2	7.5	2.9	19.2	2.6	0.9	7.9	0	0
Chhattisgarh	Korea (Koriya)	774	34	9	3	42.4	29.1	61.6	10.2	5.6	18.4	4.3	2.1	9	0	0
Chhattisgarh	Surguja	407	15	13	3	39.2	26	58.8	17.6	9.5	32.1	5.3	2.4	11.8	0	0
Chhattisgarh	Jashpur	397	18	5	3	42.9	28.4	64.2	10.3	5.5	19.4	5.2	2.4	11.2	0	0
Chhattisgarh	Raigarh	315	9	5	1	37	24.1	56.4	10.8	5.6	20.8	4.3	1.9	9.6	0	0
Chhattisgarh	Korba	825	44	12	3	48.5	33.4	69.9	11.7	6.6	20.7	4.2	2	8.7	0	0

Chhattisgarh	Janjgir - Champa	327	11	0	1	38.3	25	58.2	7.1	3.6	13.8	4.2	1.9	9.5	0	0
Chhattisgarh	Bilaspur	553	22	3	2	40.2	27	59.7	7.9	4.2	14.9	4.2	1.9	9.2	0	0
Chhattisgarh	Kabirdham	450	16	3	4	38.7	25.5	58.3	8.4	4.4	15.9	5.6	2.6	12.3	0	0
Chhattisgarh	Rajnandgaon	573	18	7	1	36.3	24.3	53.9	10.2	5.6	18.5	3.8	1.7	8.4	0	0
Chhattisgarh	Durg	732	27	3	4	38.6	25.9	57.2	7.2	3.8	13.5	4.9	2.3	10.4	0	0
Chhattisgarh	Raipur	786	36	7	3	43.8	29.9	63.8	9	5	16.3	4.3	2	9.1	0	0
Chhattisgarh	Mahasamund	367	13	2	2	39	25.7	58.6	8.3	4.2	16.2	4.8	2.1	10.8	0	0
Chhattisgarh	Dhamtari	352	14	1	1	40.6	26.4	61.8	7.5	3.9	14.7	4.2	1.8	9.7	0	0
Chhattisgarh	Uttar Bastar Kanker	334	23	1	1	52.4	34.7	78.3	7.8	4	15.3	4.3	1.9	9.8	0	0
Chhattisgarh	Bastar	465	21	6	6	43	28.5	64.3	10.5	5.6	19.7	7.1	3.3	15	0	0
Chhattisgarh	Narayanpur	631	21	4	3	36.9	24.6	55	8	4.3	15	4.6	2.2	9.6	0	0
Chhattisgarh	Dakshin Bastar Dantewada	415	29	8	8	54.3	36.1	80.8	12.6	6.9	23	8.9	4.2	18.6	0	0
Chhattisgarh	Bijapur	580	27	3	0	44.1	29.3	65.9	7.7	4.2	14.4	3.3	1.5	7.3	0	0
Dadra And Nagar Haveli	Dadra & Nagar Haveli	322	3	7	1	13.8	6.1	30.7	14.7	6.1	34.9	3	1	8.7	0	0
Daman And Diu	Diu	226	3	1	0	19	9.1	39.3	7.4	3	18.6	2.3	0.8	6.9	0	0
Daman And Diu	Daman	181	6	2	0	22.4	10.8	46.1	8.6	3.5	21.2	2.4	0.8	7.3	0	0
Goa	North Goa	211	4	0	0	16.5	7.8	34.4	5.3	2.1	13.5	2.4	0.8	7.1	0	1
Goa	South Goa	205	2	0	0	15.3	7.3	32	5.2	2	13.2	2.3	0.8	7	0	1
Gujarat	Kachchh	323	13	1	1	29.1	18.5	45.5	6.5	3.3	12.8	3.4	1.5	8	0	0
Gujarat	Banaskantha	391	20	4	0	33.7	21.7	51.9	8.1	4.1	15.8	2.9	1.2	6.7	0	0
Gujarat	Patan	323	12	2	0	28.5	18.2	44.3	7.1	3.6	13.9	3	1.3	7.2	0	0
Gujarat	Mahesana	273	5	3	2	23.4	14.6	37.2	8.1	4.1	15.7	4.1	1.8	9.6	0	0
Gujarat	Sabarkantha	319	3	1	1	21.2	13.3	33.8	6.6	3.3	13.1	3.5	1.5	7.9	0	0
Gujarat	Gandhinagar	288	8	1	2	25.6	16.4	39.8	6.7	3.3	13.3	4	1.8	9	0	0
Gujarat	Ahmadabad	221	5	0	1	24.5	15.3	39	6.4	3.2	13.1	3.7	1.6	8.5	0	0
Gujarat	Surendranagar	319	3	5	2	20.8	13	33.1	9.3	4.8	18.1	4	1.7	9.1	0	0
Gujarat	Rajkot	239	6	1	2	24.8	15.7	39	7	3.5	13.9	4	1.7	9.6	0	0
Gujarat	Jamnagar	265	8	1	0	26.1	16.6	40.7	6.9	3.4	13.8	3.1	1.3	7.5	0	0
Gujarat	Porbandar	300	4	3	0	22.1	14	34.6	8	4.1	15.3	3.1	1.3	7.3	0	0
Gujarat	Junagadh	272	2	2	1	20.8	13.1	33	7.4	3.7	14.7	3.5	1.5	8	0	0
Gujarat	Amreli	208	2	0	0	21.9	13.7	34.9	6.4	3.1	13	3.1	1.3	7.5	0	0
Gujarat	Bhavnagar	285	8	0	0	25.6	16.1	40.5	6.2	3	12.5	3.1	1.3	7.3	0	0
Gujarat	Anand	291	12	2	1	29.2	18.7	45.2	7.3	3.7	14.5	3.5	1.5	8.2	0	0
Gujarat	Kheda	338	17	5	1	32.7	20.9	50.7	9.1	4.6	17.7	3.4	1.4	8.2	0	0
Gujarat	Panchmahal	347	10	1	0	26.1	16.5	41	6.4	3.3	12.4	3	1.3	6.8	0	0
Gujarat	Dohad	368	15	7	5	29.9	18.9	46.9	10.6	5.4	20.4	5.6	2.5	12.7	0	0
Gujarat	Vadodara	283	5	6	2	23.3	14.7	36.7	10.5	5.3	20.5	4	1.7	9.2	0	0
Gujarat	Narmada	352	6	8	2	22.6	14.4	35.3	11.4	5.9	22.1	3.8	1.7	8.7	0	0
Gujarat	Bharuch	294	9	1	4	26.2	16.7	41	6.7	3.4	13.4	5.2	2.3	11.6	0	0
Gujarat	The Dangs	403	11	4	2	25.8	16.5	40.2	8.2	4.2	15.9	3.8	1.7	8.6	0	0
Gujarat	Navsari	222	4	1	1	23.4	14.6	37.2	7	3.5	13.9	3.6	1.5	8.6	0	0
Gujarat	Valsad	269	9	1	1	26.7	16.9	41.9	6.9	3.4	13.8	3.5	1.5	8	0	0
Gujarat	Surat	283	2	0	0	20.7	12.8	33.1	6.1	3.1	12.1	3.1	1.3	7.2	0	0
Gujarat	Tapi	254	3	1	0	22.1	13.9	35.2	6.9	3.4	14.1	3.1	1.3	7.3	0	0
Haryana	Panchkula	239	3	0	0	19.6	12.1	31.5	7.4	3.7	14.6	2.9	1.2	7	0	0
Haryana	Ambala	318	3	3	0	18.4	11.6	29.2	9	4.6	17.5	2.8	1.2	6.6	0	0
Haryana	Yamunanagar	285	4	0	0	19.6	12.2	31.3	7.1	3.6	13.9	2.9	1.2	6.7	0	0
Haryana	Kurukshetra	292	1	0	1	17.4	10.7	28.1	7.1	3.6	13.8	3.3	1.4	7.6	0	0
Haryana	Kaithal	392	13	6	1	24.5	15.6	38.2	10.8	5.6	20.7	3.2	1.4	7.5	0	0
Haryana	Karnal	348	6	0	1	19.9	12.5	31.6	6.9	3.5	13.5	3.2	1.4	7.5	0	0
Haryana	Panipat	283	3	2	2	18.9	11.8	30.1	8.6	4.4	16.7	3.7	1.6	8.5	0	0
Haryana	Sonipat	313	1	1	1	17.2	10.7	27.6	7.8	4	15.2	3.2	1.4	7.4	0	0
Haryana	Jind	409	7	3	1	19.8	12.4	31.6	8.7	4.5	16.9	3.1	1.4	7.2	0	0
Haryana	Fatehabad	377	10	0	0	22.3	13.9	35.4	6.8	3.5	13.1	2.8	1.2	6.7	0	0
Haryana	Sirsa	473	14	6	0	23.9	15.4	37	10	5.4	18.7	2.6	1.1	6.3	0	0

Haryana	Hisar	336	10	6	2	23.1	14.5	36.7	11.4	6	21.6	3.7	1.6	8.8	0	0
Haryana	Bhiwani	424	16	5	3	26.3	16.7	41.2	9.8	5.2	18.3	3.9	1.7	9.1	0	0
Haryana	Rohtak	367	12	4	1	24.4	15.6	38.1	9.4	5	17.6	3.1	1.3	7.3	0	0
Haryana	Jhajjar	335	9	1	0	22.4	14.2	35.2	7.7	3.9	15	2.7	1.2	6.3	0	0
Haryana	Mahendragarh	319	3	5	1	18.4	11.4	29.6	10.8	5.7	20.6	3.2	1.4	7.4	0	0
Haryana	Rewari	329	7	7	0	21	13.1	33.4	12.5	6.6	23.7	2.8	1.2	6.5	0	0
Haryana	Gurgaon	337	2	3	2	17.6	10.9	28.2	9	4.6	17.3	3.6	1.5	8.5	0	0
Haryana	Mewat	895	39	18	14	32.4	21.4	48.6	14.9	8.7	25.6	8.5	4	18	0	0
Haryana	Faridabad	337	7	3	1	20.9	13.1	33.1	9	4.6	17.5	3.2	1.4	7.4	0	0
Haryana	Palwal	474	9	8	1	20.4	12.9	32.1	11.7	6.3	21.8	3.1	1.3	7.2	0	0
Himachal Pradesh	Chamba	321	16	7	2	30.4	18.5	49.6	11.8	5.9	23.5	2.8	1.1	7.2	0	0
Himachal Pradesh	Kangra	214	6	1	0	23.9	14.3	39.5	7.8	3.7	16.5	2.2	0.9	5.8	0	0
Himachal Pradesh	Lahul And Spiti	172	3	1	0	22.1	13.1	36.8	8.1	3.9	17	2.2	0.8	6	0	0
Himachal Pradesh	Kullu	248	4	2	1	21.4	12.9	35.3	8.4	4	17.5	2.5	1	6.5	0	0
Himachal Pradesh	Mandi	263	5	2	1	22.2	13.3	36.7	8.1	3.9	16.9	2.5	1	6.5	0	0
Himachal Pradesh	Hamirpur	235	6	2	0	23.3	14.2	38.1	8.3	4	17.2	2.1	0.8	5.6	0	0
Himachal Pradesh	Una	247	4	2	1	21.4	13	35.2	8.2	4	16.8	2.6	1	6.7	0	0
Himachal Pradesh	Bilaspur	258	1	0	1	19	11.4	31.6	6.8	3.3	14	2.5	1	6.4	0	0
Himachal Pradesh	Solan	247	7	1	0	24	14.4	39.7	7.5	3.6	15.5	2.2	0.9	5.8	0	0
Himachal Pradesh	Sirmaur	305	11	3	0	26.1	15.8	42.9	8.5	4.2	17.5	2.1	0.8	5.5	0	0
Himachal Pradesh	Shimla	233	3	3	0	21	12.6	34.8	9.3	4.5	19.1	2.2	0.8	5.6	0	0
Himachal Pradesh	Kinnaur	186	5	2	0	23.2	13.7	38.9	8.7	4.2	18	2.2	0.9	5.9	0	0
Jammu And Kashmir	Kupwara	487	11	3	2	22.4	14.4	34.6	7.9	4.2	14.8	3.2	1.5	7.3	0	0
Jammu And Kashmir	Badgam	355	6	5	0	20.9	13.2	32.8	10	5.2	19.2	2.6	1.1	6.2	0	0
Jammu And Kashmir	Leh	285	4	2	1	20.5	12.8	32.6	8.2	4.1	16.2	3.1	1.3	7.3	0	0
Jammu And Kashmir	Kargil	404	11	4	2	23.9	15.2	37.4	8.8	4.6	16.8	3.3	1.4	7.6	0	0
Jammu And Kashmir	Punch	580	11	4	2	21	13.6	32.5	7.9	4.3	14.7	3.1	1.4	7.2	0	0
Jammu And Kashmir	Rajouri	455	11	3	1	23	14.7	35.7	7.8	4.1	15.1	2.9	1.3	6.7	0	0
Jammu And Kashmir	Kathua	334	10	1	1	24.2	15.2	38.3	7.3	3.7	14.4	3.1	1.4	7.1	0	0
Jammu And Kashmir	Baramula	300	5	6	0	21.3	13.4	33.7	11.4	5.9	21.8	2.7	1.1	6.4	0	0
Jammu And Kashmir	Bandipore	387	11	7	3	24.2	15.1	38.4	11.4	6	21.5	3.9	1.7	9	0	0
Jammu And Kashmir	Srinagar	242	6	2	1	22.8	14.3	36.2	8.5	4.3	16.7	3.2	1.3	7.7	0	0
Jammu And Kashmir	Ganderbal	426	13	4	2	24.7	15.8	38.6	8.7	4.6	16.7	3.4	1.5	7.7	0	0
Jammu And Kashmir	Pulwama	286	7	4	1	23.1	14.4	36.6	9.5	4.9	18.2	3.2	1.4	7.3	0	0
Jammu And Kashmir	Shupiyan	309	6	1	0	21.8	13.7	34.4	7.3	3.7	14.4	2.7	1.1	6.4	0	0
Jammu And Kashmir	Anantnag	327	9	6	1	23.9	15	37.7	11.1	5.8	21.1	3.1	1.3	7	0	0
Jammu And Kashmir	Kulgam	347	11	7	2	24.9	15.8	39.1	11.7	6.1	22.4	3.5	1.5	8	0	0
Jammu And Kashmir	Doda	403	10	5	1	23.3	14.9	36.3	9.5	5	18	3	1.3	6.7	0	0
Jammu And Kashmir	Ramban	450	13	4	1	24.5	15.8	37.7	8.6	4.6	16.2	2.9	1.3	6.5	0	0
Jammu And Kashmir	Kishtwar	399	11	5	2	23.8	15.3	37	9.7	5.1	18.1	3.5	1.5	8.1	0	0
Jammu And Kashmir	Udhampur	395	11	0	0	23.9	15.3	37.2	6.4	3.3	12.5	2.6	1.1	5.8	0	0
Jammu And Kashmir	Reasi	491	11	1	2	22.5	14.4	35	6.6	3.4	12.7	3.2	1.4	7.3	0	0
Jammu And Kashmir	Jammu	255	2	0	2	19.8	12.4	31.4	7	3.5	13.9	3.6	1.6	8.5	0	0
Jammu And Kashmir	Samba	328	2	1	1	18.3	11.3	29.6	7.2	3.7	14	3.1	1.3	7.3	0	0
Jharkhand	Garhwa	546	27	5	2	39.2	26.2	58.3	9.1	5	16.5	4.5	2.1	9.5	0	0
Jharkhand	Chatra	624	27	5	2	36.6	24.6	54.2	8.6	4.6	15.9	4.2	1.9	9.1	0	0
Jharkhand	Kodarma	506	10	4	1	26.3	17.3	39.8	8.6	4.7	15.6	4	1.8	8.8	0	0
Jharkhand	Giridih	559	19	5	5	32.1	21.1	48.5	8.9	4.8	16.5	6.2	3	12.6	0	0
Jharkhand	Deoghar	558	23	3	3	35.5	23.6	52.9	7.8	4.2	14.3	4.9	2.3	10.5	0	0
Jharkhand	Godda	436	16	4	6	33	21.9	49.4	8.9	4.8	16.4	7.4	3.4	15.7	0	0
Jharkhand	Sahibganj	522	20	9	3	33.7	22.4	50.3	12.2	6.7	21.9	5.1	2.3	10.9	0	0
Jharkhand	Pakur	526	13	8	4	28.1	18.3	42.9	11.4	6.3	20.7	5.6	2.7	11.7	0	0
Jharkhand	Dhanbad	689	24	4	3	32.5	21.7	48.3	7.7	4.2	14.1	4.5	2.1	9.5	0	0
Jharkhand	Bokaro	719	18	7	1	27.9	18.7	41.4	9.2	5.1	16.8	3.7	1.7	7.8	0	0
Jharkhand	Lohardaga	420	6	1	2	24.6	15.9	38	7.2	3.7	13.8	4.7	2.2	10.1	0	0

Jharkhand	Purbi Singhbhum	543	6	1	0	22.8	14.9	34.8	6.7	3.6	12.5	3.5	1.6	7.8	0	0
Jharkhand	Palamu	443	15	5	2	31.8	20.9	48.1	9.6	5.2	17.9	4.6	2.1	10.3	0	0
Jharkhand	Latehar	437	11	5	1	28.5	18.6	43.4	9.6	5.2	17.9	4.2	2	9.1	0	0
Jharkhand	Hazaribagh	474	16	4	2	31.6	20.7	48	8.9	4.7	16.8	4.6	2.1	9.9	0	0
Jharkhand	Ramgarh	708	21	4	4	30	20.1	44.4	7.6	4.1	14.1	5.1	2.4	10.7	0	0
Jharkhand	Dumka	385	20	6	0	38.4	25.2	58	10.9	5.9	20.1	3.7	1.6	8.4	0	0
Jharkhand	Jamtara	515	19	4	3	33.1	22	49.5	8.5	4.4	16.4	5	2.4	10.6	0	0
Jharkhand	Ranchi	580	17	6	2	29.8	19.8	44.8	9.4	5.1	17.4	4.4	2	9.5	0	0
Jharkhand	Khunti	391	4	4	1	23.8	15.2	37.1	9.3	5	17.2	4.3	1.9	9.6	0	0
Jharkhand	Gumla	405	11	1	3	29.2	19	44.6	7.2	3.7	14	5.3	2.5	11.5	0	0
Jharkhand	Simdega	386	16	4	5	34.5	22.6	52.3	9.3	4.9	17.4	6.8	3.2	14.6	0	0
Jharkhand	Pashchimi Singhbhum	450	16	12	8	32.2	21.1	48.9	15.5	8.5	28	8.8	4.2	18.5	0	0
Jharkhand	Saraikela Kharsawan	382	11	6	4	29.8	19.5	45.2	10.7	5.8	19.9	6.1	2.8	13.1	0	0
Karnataka	Belgaum	311	4	2	1	18.5	11.5	29.6	7.6	3.9	14.9	2.6	1.1	6.2	0	0
Karnataka	Bagalkot	329	8	3	1	20.8	13.2	32.7	8.1	4.1	15.8	2.6	1.1	6.1	0	0
Karnataka	Bijapur	325	8	3	0	20.9	13.1	33	8.1	4.2	15.8	2.2	0.9	5.2	0	0
Karnataka	Bidar	298	9	2	0	22.3	14.1	35.2	7.6	3.9	14.9	2.2	0.9	5.4	0	0
Karnataka	Raichur	368	14	3	0	24.9	15.7	39.4	8	4.1	15.6	2.2	0.9	5.3	0	0
Karnataka	Koppal	332	5	5	2	18.7	11.6	29.9	9.6	5	18.4	3	1.3	6.9	0	0
Karnataka	Gadag	267	4	2	0	18.8	11.6	30.4	7.8	3.9	15.5	2.3	1	5.5	0	0
Karnataka	Dharwad	283	9	4	0	22.6	14	36.2	9	4.6	17.9	2.3	0.9	5.6	0	0
Karnataka	Uttara Kannada	219	4	1	0	19.5	12.1	31.4	7.3	3.7	14.4	2.3	0.9	5.5	0	0
Karnataka	Haveri	261	2	1	1	17.7	10.9	28.7	7.1	3.6	13.8	2.6	1.1	6.3	0	0
Karnataka	Bellary	348	12	0	3	23.8	14.9	37.9	6.3	3.2	12.3	3.3	1.3	7.9	0	0
Karnataka	Chitradurga	209	4	8	0	19.7	12.1	31.9	13.9	7.1	26.9	2.3	1	5.5	0	0
Karnataka	Davanagere	282	7	4	1	20.8	13	33.1	9.3	4.8	17.9	2.6	1.1	6.4	0	0
Karnataka	Shimoga	220	6	2	0	21.4	13.3	34.1	8.1	4.1	16.1	2.4	1	5.6	0	0
Karnataka	Udupi	198	4	0	0	19.8	12.1	32.2	6.8	3.3	13.6	2.3	1	5.5	0	0
Karnataka	Chikmagalur	183	0	1	0	17	10.3	27.8	7.5	3.8	15	2.3	0.9	5.7	0	0
Karnataka	Tumkur	199	12	4	0	26.8	16.6	43.1	9.8	4.8	19.7	2.3	1	5.5	0	0
Karnataka	Bangalore	226	2	2	1	18	11.2	28.8	8	4.1	15.7	2.7	1.1	6.4	0	0
Karnataka	Mandya	181	4	1	1	20.2	12.5	32.7	7.4	3.7	14.8	2.7	1.1	6.3	0	0
Karnataka	Hassan	200	3	0	1	19.2	11.9	30.8	6.8	3.4	13.6	2.6	1.1	6.1	0	0
Karnataka	Dakshina Kannada	208	2	0	1	18.3	11.2	29.6	6.8	3.4	13.5	2.6	1.1	6.4	0	0
Karnataka	Kodagu	233	3	0	0	18.7	11.7	29.8	6.6	3.4	13.1	2.3	1	5.7	0	0
Karnataka	Mysore	235	3	2	1	18.7	11.4	30.5	8	4.1	15.7	2.7	1.1	6.5	0	0
Karnataka	Chamarajanagar	215	5	4	2	20.4	12.7	32.5	9.7	4.9	18.8	3	1.3	7.2	0	0
Karnataka	Gulbarga	366	5	2	0	18.2	11.5	28.9	7.1	3.7	13.9	2.2	0.9	5.2	0	0
Karnataka	Yadgir	379	5	3	0	18.2	11.2	29.3	7.8	4.1	15.1	2.2	0.9	5.3	0	0
Karnataka	Kolar	258	8	3	1	21.9	13.8	34.6	8.6	4.4	16.8	2.6	1.1	6.3	0	0
Karnataka	Chikkaballapura	211	2	1	2	18.5	11.3	30	7.4	3.6	15.1	3.1	1.3	7.4	0	0
Karnataka	Bangalore Rural	253	5	2	0	20.1	12.5	32.3	7.9	4	15.5	2.3	1	5.5	0	0
Karnataka	Ramanagara	192	2	0	1	18.5	11.4	30	6.8	3.3	13.8	2.7	1.1	6.7	0	0
Kerala	Kasaragod	208	0	2	0	6	3.1	11.5	4.7	2	10.8	1.8	0.6	4.9	1	1
Kerala	Kannur	180	1	0	1	6.5	3.4	12.4	3.9	1.7	9	2	0.7	5.6	1	1
Kerala	Wayanad	235	2	3	0	6.5	3.4	12.6	5.1	2.2	11.8	1.7	0.6	4.7	1	1
Kerala	Kozhikode	175	2	0	0	6.7	3.4	13	3.9	1.7	9.2	1.8	0.6	4.9	1	1
Kerala	Malappuram	263	1	0	0	6.2	3.2	11.9	3.8	1.6	8.8	1.8	0.6	4.8	1	1
Kerala	Palakkad	216	0	0	0	6	3.1	11.5	3.9	1.6	9.2	1.8	0.6	5	1	1
Kerala	Thrissur	144	0	0	0	6.1	3.2	11.7	4	1.7	9.2	1.8	0.7	4.9	1	1
Kerala	Ernakulam	152	0	0	1	6.2	3.2	11.9	3.9	1.7	9.1	2	0.7	5.6	1	1
Kerala	Idukki	129	0	0	0	6.2	3.2	11.9	4	1.7	9.2	1.9	0.7	5.2	1	1
Kerala	Kottayam	161	1	0	0	6.5	3.4	12.5	3.9	1.6	9.1	1.8	0.6	4.9	1	1
Kerala	Alappuzha	140	2	0	0	6.8	3.5	13.1	3.9	1.7	9.2	1.8	0.6	5.1	1	1
Kerala	Pathanamthitta	132	0	0	0	6.1	3.2	11.8	4	1.7	9.3	1.7	0.6	4.8	1	1

Kerala	Kollam	163	1	0	0	6.5	3.3	12.5	3.9	1.7	9.1	1.8	0.6	4.9	1	1
Kerala	Thiruvananthapuram	164	1	1	0	6.4	3.3	12.1	4.3	1.8	10.1	1.8	0.6	5	1	1
Lakshadweep	Lakshadweep	308	7	1	0	21.2	9.7	45.5	6.3	2.5	15.9	2.4	0.8	7.1	0	0
Madhya Pradesh	Sheopur	501	14	9	5	31.7	21.3	46.9	14.8	8.4	25.9	7.1	3.5	14.6	0	0
Madhya Pradesh	Morena	452	14	10	4	33.1	22.2	49.2	16.2	9.3	28	6.6	3.1	13.8	0	0
Madhya Pradesh	Bhind	450	18	4	2	36.8	24.8	54.5	11.1	6.1	19.9	5.4	2.6	11.4	0	0
Madhya Pradesh	Gwalior	851	40	19	6	41.8	28.9	59.9	18	11	29.2	6.3	3.3	12.2	0	0
Madhya Pradesh	Datia	419	17	7	5	36.9	24.6	55.2	13.9	7.8	24.5	7.7	3.8	15.5	0	0
Madhya Pradesh	Shivpuri	475	18	8	2	36.1	24.5	53	14.3	8.2	24.7	5.3	2.5	10.9	0	0
Madhya Pradesh	Tikamgarh	421	14	6	2	34.2	23	50.7	13.1	7.4	23	5.5	2.6	11.7	0	0
Madhya Pradesh	Chhatarpur	459	15	12	5	33.9	22.7	50.3	18.2	10.6	31	7.5	3.8	14.8	0	0
Madhya Pradesh	Panna	405	24	6	2	44.4	30	65.2	13.2	7.5	23.3	5.5	2.6	11.4	0	0
Madhya Pradesh	Sagar	442	21	2	2	39.9	26.9	58.8	9.6	5.2	18	5.3	2.5	11.3	0	0
Madhya Pradesh	Damoh	388	13	8	1	34.1	22.6	51.3	15.3	8.6	27	5	2.3	10.7	0	0
Madhya Pradesh	Satna	364	12	4	1	33.8	22.2	51.2	11.9	6.6	21.5	5.1	2.4	10.8	0	0
Madhya Pradesh	Rewa	460	26	11	5	44.3	30.2	64.6	17.1	9.8	29.7	7.3	3.6	14.6	0	0
Madhya Pradesh	Umaria	406	15	6	5	35.4	23.7	52.7	13.1	7.3	23.7	7.6	3.7	15.7	0	0
Madhya Pradesh	Neemuch	376	7	6	1	28.9	18.7	44.2	13.7	7.7	24.2	5	2.3	10.5	0	0
Madhya Pradesh	Mandsaur	412	14	7	0	34.1	22.8	50.9	14.1	7.8	25.5	4.3	2	9.2	0	0
Madhya Pradesh	Ratlam	424	12	2	1	32	21	48.6	9.7	5.4	17.4	4.9	2.3	10.2	0	0
Madhya Pradesh	Ujjain	945	28	7	4	31.8	21.9	46	9.8	5.8	16.4	5	2.6	9.7	0	0
Madhya Pradesh	Shajapur	410	12	4	3	32.4	21.4	48.7	11.4	6.4	20.4	6.1	3	12.6	0	0
Madhya Pradesh	Dewas	456	24	5	2	42.2	28.4	62.3	11.8	6.6	21	5.4	2.5	11.3	0	0
Madhya Pradesh	Dhar	505	26	4	5	42.7	29.1	62.4	10.5	5.8	19	7.2	3.6	14.3	0	0
Madhya Pradesh	Indore	771	13	5	0	25.5	17.2	37.6	9.5	5.4	16.6	3.7	1.7	7.9	0	0
Madhya Pradesh	Khargone (West Nimar)	448	17	2	3	36.2	24.3	53.7	9.5	5.2	17.3	6	2.9	12.5	0	0
Madhya Pradesh	Barwani	706	27	4	3	36.4	24.9	52.9	9.2	5.2	16.2	5.2	2.6	10.4	0	0
Madhya Pradesh	Rajgarh	432	16	8	2	35.9	24	53.4	14.7	8.3	25.7	5.4	2.6	11.3	0	0
Madhya Pradesh	Vidisha	469	16	16	2	34.4	23.1	50.9	22.5	13.4	37.6	5.3	2.6	11	0	0
Madhya Pradesh	Bhopal	361	13	2	3	35	23.3	52.2	10.3	5.6	18.8	6.6	3.2	13.6	0	0
Madhya Pradesh	Sehore	436	11	7	3	31	20.6	46.5	13.8	7.8	24.1	6	2.9	12.4	0	0
Madhya Pradesh	Raisen	491	15	4	3	32.7	22	48.4	10.6	5.9	19	5.8	2.9	11.8	0	0
Madhya Pradesh	Betul	369	8	5	3	30.2	19.8	45.9	12.6	6.9	22.9	6.3	3	13	0	0
Madhya Pradesh	Harda	438	10	5	6	29.9	19.9	44.6	12	6.8	21.3	8.3	4.1	16.5	0	0
Madhya Pradesh	Hoshangabad	775	23	4	5	31.8	21.9	46.2	9	5.1	16	6	3.1	11.9	0	0
Madhya Pradesh	Katni	349	17	11	1	40	26.5	59.9	19.5	11	34.3	5	2.3	10.6	0	0
Madhya Pradesh	Jabalpur	663	29	3	4	39.4	27.1	57	8.9	4.9	16.1	5.8	2.9	11.7	0	0
Madhya Pradesh	Narsimhapur	333	13	1	1	36.3	24.2	54.2	9.7	5.2	17.8	5.1	2.4	11	0	0
Madhya Pradesh	Dindori	385	15	6	1	36.5	24.5	53.9	13.3	7.5	23.7	5	2.4	10.5	0	0
Madhya Pradesh	Mandla	340	14	2	3	36.7	24.2	55.2	10.4	5.7	19	6.5	3.1	13.5	0	0
Madhya Pradesh	Chhindwara	373	19	9	1	40.5	27.2	59.9	16.6	9.4	29.1	5	2.4	10.5	0	0
Madhya Pradesh	Seoni	348	11	6	2	33.2	21.8	50.3	14.1	7.8	25.5	5.7	2.7	11.8	0	0
Madhya Pradesh	Balaghat	392	18	3	1	38.9	25.7	58.6	10.8	5.9	19.5	5	2.3	10.5	0	0
Madhya Pradesh	Guna	558	21	8	4	35.8	24.4	52.2	13.2	7.7	22.6	6.1	3	12.5	0	0
Madhya Pradesh	Ashoknagar	469	11	5	3	30.1	20	44.9	11.6	6.5	20.6	6	2.9	12.3	0	0
Madhya Pradesh	Shahdol	320	10	8	5	33.3	21.8	50.5	16.5	9.3	29.2	8.2	4	17	0	0
Madhya Pradesh	Anuppur	387	12	4	2	33.2	22	49.7	11.7	6.4	21.2	5.6	2.6	11.8	0	0
Madhya Pradesh	Sidhi	525	22	5	11	38.1	25.8	55.9	11.3	6.3	20.1	11.8	6.2	22.3	0	0
Madhya Pradesh	Singrauli	585	14	13	7	29.4	19.6	43.7	17.2	10.1	29.2	8	4	16.1	0	0
Madhya Pradesh	Jhabua	594	19	6	7	33.2	22.4	48.8	11.2	6.4	19.7	8	4	15.7	0	0
Madhya Pradesh	Alirajpur	838	30	10	7	35.2	24.5	50.3	12.1	7.2	20.2	7	3.7	13.1	0	0
Madhya Pradesh	Khandwa (East Nimar)	484	12	4	0	30.5	20.2	45.7	10.8	6.1	19.1	4.2	2	8.8	0	0
Madhya Pradesh	Burhanpur	951	34	13	5	35.3	24.5	50.5	13.1	7.8	21.9	5.5	2.9	10.7	0	0
Maharashtra	Nandurbar	331	10	1	1	21	13.1	33.4	5.3	2.7	10.4	2	0.8	4.9	0	0
Maharashtra	Dhule	299	3	2	1	16.6	10.4	26.6	6	3	11.8	2	0.8	4.9	0	1

Maharashtra	Jalgaon	263	9	0	1	21	13	33.6	5.1	2.5	10.3	2	0.8	4.8	0	0
Maharashtra	Buldana	288	3	1	1	16.6	10.3	26.4	5.5	2.8	10.8	2	0.8	4.9	0	1
Maharashtra	Akola	323	9	2	0	20.2	12.5	32.4	5.9	3	11.6	1.7	0.7	4.1	0	0
Maharashtra	Washim	306	4	3	0	17.1	10.6	27.2	6.5	3.3	13	1.7	0.7	4.1	0	0
Maharashtra	Amravati	232	3	1	0	17.3	10.7	27.9	5.6	2.8	11.3	1.7	0.7	4.2	0	1
Maharashtra	Wardha	192	4	1	0	18.3	11.3	29.8	5.7	2.8	11.5	1.7	0.7	4.3	0	0
Maharashtra	Nagpur	285	2	1	1	15.9	9.9	25.3	5.4	2.7	10.9	2	0.8	4.9	0	1
Maharashtra	Bhandara	210	9	1	0	21.9	13.6	35.1	5.7	2.8	11.4	1.7	0.7	4.2	0	0
Maharashtra	Gondiya	263	3	2	0	17	10.6	27.1	6.2	3.1	12.3	1.7	0.7	4.1	0	1
Maharashtra	Gadchiroli	217	6	0	0	19.6	12.1	31.6	5.2	2.6	10.4	1.8	0.7	4.4	0	0
Maharashtra	Chandrapur	221	3	1	1	17.5	10.7	28.6	5.6	2.8	11.3	2	0.8	5	0	0
Maharashtra	Yavatmal	280	5	1	2	18.1	11.3	28.7	5.4	2.6	11	2.3	1	5.7	0	0
Maharashtra	Nanded	420	12	3	0	21.1	13.3	33.2	6.1	3.1	11.9	1.7	0.7	3.9	0	0
Maharashtra	Hingoli	325	12	2	1	22.2	13.9	35.1	6	3	11.8	2	0.8	4.9	0	0
Maharashtra	Parbhani	359	11	2	1	21.2	13.4	33.4	5.8	2.9	11.4	1.9	0.8	4.8	0	0
Maharashtra	Jalna	366	8	2	0	18.9	11.8	30.1	5.8	2.9	11.5	1.7	0.7	4.1	0	0
Maharashtra	Aurangabad	318	2	6	1	15.8	9.8	25.3	8.5	4.2	17.1	2	0.8	4.8	0	0
Maharashtra	Nashik	347	4	3	3	16.7	10.5	26.6	6.3	3.3	12.1	2.6	1.1	6.3	0	0
Maharashtra	Thane	226	3	1	0	17.4	10.8	28	5.7	2.8	11.4	1.7	0.7	4.2	0	1
Maharashtra	Mumbai Suburban	172	2	3	0	17.1	10.4	28.1	7.2	3.5	14.6	1.7	0.7	4.3	0	0
Maharashtra	Mumbai	163	3	1	0	17.9	11.1	28.8	5.8	2.8	11.9	1.8	0.7	4.5	0	0
Maharashtra	Raigarh	221	7	2	0	20.4	12.7	32.5	6.2	3.1	12.4	1.7	0.7	4.2	0	0
Maharashtra	Pune	232	4	0	1	18	11.2	28.8	5.2	2.5	10.6	2	0.8	4.9	0	0
Maharashtra	Ahmadnagar	273	2	1	0	16.2	10	26	5.5	2.8	11.1	1.7	0.7	4.1	0	1
Maharashtra	Bid	322	7	2	0	18.8	11.9	29.7	5.9	3.1	11.6	1.7	0.7	4.2	0	0
Maharashtra	Latur	368	5	1	0	17	10.6	27.1	5.2	2.6	10.4	1.7	0.7	4.1	0	1
Maharashtra	Osmanabad	264	5	0	1	18.2	11.1	29.8	5.1	2.5	10.3	2	0.8	4.8	0	0
Maharashtra	Solapur	311	3	2	0	16.5	10.3	26.2	5.9	3	11.6	1.7	0.7	4.2	0	1
Maharashtra	Satara	219	3	2	0	17.5	10.9	28.1	6.3	3.1	12.8	1.7	0.7	4.2	0	0
Maharashtra	Ratnagiri	186	3	0	0	18	11	29.4	5.2	2.5	10.6	1.8	0.7	4.4	0	0
Maharashtra	Sindhudurg	154	3	1	0	18.3	11.4	29.3	6	2.9	12.3	1.8	0.7	4.5	0	0
Maharashtra	Kolhapur	204	0	3	0	15.7	9.7	25.2	7	3.5	14	1.7	0.7	4.2	0	1
Maharashtra	Sangli	241	3	2	0	17.1	10.5	27.7	6.2	3.1	12.4	1.7	0.7	4.2	0	0
Manipur	Senapati (Excluding 3 Sub-Divisions)	525	11	4	1	17.8	11.1	28.4	6.3	3.1	12.6	2.3	1	5.5	0	0
Manipur	Tamenglong	562	11	2	1	17.4	10.8	28.1	5.3	2.6	10.9	2.3	0.9	5.5	0	0
Manipur	Churachandpur	432	10	4	1	18.3	11	30.1	6.6	3.3	13.2	2.4	1	5.7	0	0
Manipur	Bishnupur	852	13	4	6	16.1	10.2	25.5	5.4	2.7	10.7	3.7	1.6	8.3	0	0
Manipur	Thoubal	862	17	3	1	17.8	11.2	28.3	5	2.5	9.9	2.1	0.9	5	0	1
Manipur	Imphal West	729	7	2	0	14.1	8.6	22.9	4.8	2.4	9.8	1.9	0.8	4.5	0	1
Manipur	Imphal East	771	11	5	1	15.7	9.8	25.1	6	3.1	11.9	2.1	0.9	5	0	1
Manipur	Ukhrul	442	9	2	1	17.5	10.6	28.9	5.6	2.7	11.4	2.4	1	5.9	0	0
Manipur	Chandel	461	5	5	1	15	9.1	24.6	7	3.5	13.9	2.3	1	5.6	0	1
Meghalaya	West Garo Hills	392	13	1	1	21.3	12.9	35.1	8.5	4.3	16.9	3.9	1.6	9.2	0	0
Meghalaya	East Garo Hills	564	1	6	4	12.9	7.7	21.7	11	5.7	21.2	5.1	2.2	11.6	0	0
Meghalaya	South Garo Hills	374	2	8	6	14.7	8.7	24.8	14.6	7.7	27.8	7.1	3.1	16.2	0	0
Meghalaya	West Khasi Hills	679	15	6	1	18.8	11.5	30.7	10.1	5.3	19.2	3.5	1.5	8	0	0
Meghalaya	Ribhoi	648	15	13	3	19.1	11.6	31.2	15.5	8.3	28.8	4.4	1.8	10.3	0	0
Meghalaya	East Khasi Hills	964	12	11	3	15.1	9.2	24.6	11.1	6	20.4	3.8	1.7	8.7	0	0
Meghalaya	Jaintia Hills	788	19	11	5	19.9	12.2	32.3	12.5	6.6	23.6	4.9	2.2	11	0	0
Mizoram	Mamit	403	2	7	4	10.4	6.1	17.6	21.1	11.8	37.4	4.6	1.9	10.7	1	0
Mizoram	Kolasib	760	7	28	3	10.9	6.4	18.4	33	19.9	54.4	3.4	1.4	8	1	0
Mizoram	Aizawl	342	4	7	1	11.6	6.8	19.8	23.2	12.6	42.4	3.2	1.3	7.7	1	0
Mizoram	Champhai	858	14	19	1	13.2	7.9	21.7	22.9	13.6	38.4	2.7	1.1	6.2	0	0
Mizoram	Serchhip	697	9	15	3	11.9	7.1	20	22.7	13.2	38.8	3.6	1.6	8.2	1	0

Mizoram	Lunglei	568	4	22	3	10.6	6.4	17.7	33.5	19.8	56.2	3.7	1.6	8.8	1	0
Mizoram	Lawngtlai	394	6	12	1	12.2	7.2	20.7	27.5	15.4	48.8	3.2	1.4	7.6	0	0
Mizoram	Saiha	883	9	48	2	11.1	6.6	18.6	47.6	29.4	76.2	3	1.3	6.8	1	0
Nagaland	Mon	576	0	5	2	12.4	7.5	20.4	10.5	5.5	20	4	1.8	9.2	0	0
Nagaland	Mokokchung	158	5	1	0	19	11.3	31.6	10.8	5.2	22.2	3.9	1.5	9.8	0	0
Nagaland	Zunheboto	268	4	1	0	16.7	10.1	27.6	9.9	4.9	19.7	3.6	1.5	8.7	0	0
Nagaland	Wokha	247	6	2	2	18.3	11	30.3	10.7	5.5	20.9	4.8	2	11.7	0	0
Nagaland	Dimapur	728	17	10	2	19.5	12.1	31.1	12.7	6.9	23.3	3.7	1.6	8.6	0	0
Nagaland	Phek	402	4	8	7	15.6	9.6	25.5	14.6	7.6	27.9	7.6	3.3	17.7	0	0
Nagaland	Tuensang	454	7	8	2	16.7	10.3	27	14	7.4	26.4	4.2	1.8	9.9	0	0
Nagaland	Longleng	342	4	2	1	15.9	9.5	26.7	10	5.1	19.6	4	1.7	9.5	0	0
Nagaland	Kiphire	442	16	10	2	22.6	13.8	36.7	16	8.5	29.9	4.3	1.8	9.9	0	0
Nagaland	Kohima	541	9	7	2	17.1	10.4	27.9	11.9	6.4	22.2	4	1.7	9.3	0	0
Nagaland	Peren	449	9	8	4	17.9	10.9	29.1	13.9	7.5	25.8	5.4	2.3	12.4	0	0
Delhi	North West	155	3	2	0	17.2	9.6	30.6	11.2	5.2	23.7	3.4	1.3	8.8	0	0
Delhi	North	217	7	1	0	19.3	10.9	33.9	9.7	4.6	20.3	3.3	1.3	8.6	0	0
Delhi	North East	258	4	6	1	16.8	9.5	29.6	14	6.8	28.5	3.7	1.4	9.6	0	0
Delhi	East	165	1	5	0	15.8	9	27.7	14.6	6.9	30.6	3.4	1.3	8.9	0	0
Delhi	New Delhi	119	1	1	0	16.3	9.2	28.9	10.6	4.9	23	3.5	1.4	9.1	0	0
Delhi	Central	140	3	3	0	17.7	10	31.2	12.5	6	26	3.4	1.3	9	0	0
Delhi	West	148	2	1	0	16.6	9.3	29.5	10.4	4.9	22.2	3.5	1.3	9.2	0	0
Delhi	South West	211	1	0	2	15.3	8.6	26.9	8.9	4.2	18.8	4.5	1.8	11.4	0	0
Delhi	South	167	5	2	5	18.6	10.5	32.6	11.2	5.2	23.9	7	2.7	18.2	0	0
Odisha	Bargarh	312	7	5	0	27.7	17.9	42.7	11.6	6.2	21.8	3	1.3	6.8	0	0
Odisha	Jharsuguda	545	16	5	2	29.7	19.7	44.4	9.6	5.2	17.6	3.5	1.6	7.7	0	0
Odisha	Sambalpur	285	10	0	1	31.3	20.1	48.4	7.8	4	15	3.5	1.5	7.9	0	0
Odisha	Debagarh	350	10	6	0	29.5	19.1	45.2	12	6.4	22.2	3	1.3	6.8	0	0
Odisha	Sundargarh	578	11	3	4	25.1	16.3	38.4	8.1	4.5	14.8	4.4	2	9.4	0	0
Odisha	Kendujhar	391	17	2	1	34.8	22.8	52.8	8.5	4.4	16.3	3.3	1.5	7.5	0	0
Odisha	Mayurbhanj	337	3	7	3	23.6	15	37	13.3	7.1	24.7	4.5	2	10.1	0	0
Odisha	Baleshwar	316	7	0	1	27.6	17.7	42.8	7.5	4	14.2	3.5	1.5	7.9	0	0
Odisha	Bhadrak	339	9	2	1	28.8	18.3	44.9	8.9	4.6	17.1	3.4	1.5	7.8	0	0
Odisha	Kendrapara	357	5	6	0	25	15.9	39.2	11.8	6.3	22	3	1.3	6.9	0	0
Odisha	Jagatsinghapur	261	8	3	0	30.1	19.4	46.6	10.2	5.3	19.5	3.1	1.3	7.2	0	0
Odisha	Cuttack	271	4	3	0	25.8	16.3	40.5	10.3	5.4	19.4	3.1	1.3	7.1	0	0
Odisha	Jajapur	330	9	3	0	29.1	18.7	45	9.8	5.1	18.7	3	1.3	6.8	0	0
Odisha	Dhenkanal	323	12	1	0	32.2	20.7	49.8	8.2	4.3	15.7	3	1.3	6.8	0	0
Odisha	Anugul	361	19	1	2	38.1	25	57.7	7.9	4.1	15	3.8	1.7	8.5	0	0
Odisha	Nayagarh	299	12	1	1	33	21.3	50.9	8.4	4.3	16.4	3.5	1.6	8	0	0
Odisha	Khordha	533	6	3	3	22.7	14.6	34.9	8.4	4.5	15.4	4.2	1.9	9.1	0	0
Odisha	Puri	320	12	2	0	32.3	21.2	48.8	9	4.7	17	3	1.3	6.9	0	0
Odisha	Ganjam	289	5	5	0	26.7	17.1	41.2	11.6	6.3	21.5	3	1.3	7	0	0
Odisha	Gajapati	363	14	3	2	32.4	21.1	49.3	9.3	4.9	17.6	3.9	1.7	8.6	0	0
Odisha	Kandhamal	427	20	6	2	36.5	23.9	55.4	11.3	6.2	20.4	3.7	1.7	8.3	0	0
Odisha	Baudh	368	16	6	5	34.7	22.9	52.3	11.9	6.3	22.1	5.5	2.5	12.1	0	0
Odisha	Subarnapur	322	7	5	0	27.3	17.7	41.9	11.4	6.2	21	3	1.3	6.8	0	0
Odisha	Balangir	344	12	2	1	31.6	20.5	48.4	8.8	4.6	16.6	3.3	1.5	7.5	0	0
Odisha	Nuapada	424	15	1	0	31.8	21	47.8	7.7	4	14.6	2.9	1.3	6.6	0	0
Odisha	Kalahandi	317	9	6	1	29.4	18.8	45.7	12.4	6.7	22.9	3.5	1.5	7.9	0	0
Odisha	Rayagada	371	20	8	4	38.4	25.4	57.5	13.6	7.4	24.9	5	2.3	10.8	0	0
Odisha	Nabarangapur	497	23	6	4	36.8	24.3	55.4	10.7	5.9	19.3	4.6	2.2	9.7	0	0
Odisha	Koraput	375	10	2	1	28.9	18.7	44.4	8.6	4.6	15.8	3.3	1.5	7.4	0	0
Odisha	Malkangiri	501	17	18	6	31.5	20.8	47.3	21.5	12.3	37.3	5.6	2.6	12.1	0	0
Puducherry	Yanam	281	3	3	3	11.2	5.7	21.9	8	3.5	18.1	3.8	1.4	10.1	1	1
Puducherry	Puducherry	258	2	4	0	10.8	5.4	21.4	8.9	4	19.7	2.6	1	6.9	1	1

Puducherry	Mahe	270	1	0	0	10.5	5.3	20.7	6	2.6	13.6	2.5	0.9	6.8	1	1
Puducherry	Karaikal	272	4	0	0	11.7	5.9	23.4	6.1	2.7	14	2.5	0.9	6.9	1	1
Punjab	Gurdaspur	252	6	2	0	22.7	13.9	36.9	7.6	3.8	15.2	2	0.8	4.9	0	0
Punjab	Kapurthala	221	5	1	2	22.5	13.9	36.1	7.1	3.4	14.5	2.7	1	6.9	0	0
Punjab	Jalandhar	230	5	3	0	22.1	13.6	35.7	8.6	4.3	17.2	2	0.8	5.1	0	0
Punjab	Hoshiarpur	224	4	2	0	21.6	13.2	35.2	7.8	3.8	15.6	2	0.8	4.9	0	0
Punjab	Sangrur	222	6	1	1	23.2	14.5	37	7.1	3.5	14.5	2.3	0.9	5.8	0	0
Punjab	Fatehgarh Sahib	227	5	3	1	22.2	13.6	36.2	8.5	4.3	16.6	2.3	0.9	6	0	0
Punjab	Ludhiana	251	7	2	1	23.3	14.4	37.6	7.7	3.8	15.5	2.3	0.9	5.7	0	0
Punjab	Moga	298	10	3	2	25	15.5	39.8	8.2	4.2	15.8	2.6	1.1	6.3	0	0
Punjab	Firozpur	275	8	1	1	23.8	14.7	38.4	7	3.5	14	2.3	0.9	5.6	0	0
Punjab	Muktsar	265	5	8	1	21.8	13.4	35.1	12.6	6.4	24.5	2.3	0.9	5.8	0	0
Punjab	Faridkot	290	6	1	0	22	13.6	35.3	6.8	3.4	13.5	1.9	0.8	4.9	0	0
Punjab	Bathinda	241	4	2	0	21.2	13	34.3	7.8	3.9	15.5	2	0.8	5	0	0
Punjab	Mansa	292	7	2	0	22.7	14.1	36.2	7.4	3.6	15	1.9	0.8	4.8	0	0
Punjab	Patiala	295	4	4	0	20.5	12.7	33	9	4.6	17.6	1.9	0.8	4.8	0	0
Punjab	Amritsar	304	5	2	0	20.8	13	33.2	7.4	3.7	14.6	2	0.8	4.9	0	0
Punjab	Tarn Taran	309	8	2	1	23.1	14.4	37	7.3	3.6	14.7	2.2	0.9	5.7	0	0
Punjab	Rupnagar	243	6	1	0	22.9	14.1	36.8	7.1	3.5	14.4	2	0.8	5	0	0
Punjab	Sahibzada Ajit Singh Nagar	265	7	1	0	23	14.1	37.3	6.9	3.5	13.9	2	0.8	5	0	0
Punjab	Shahid Bhagat Singh Nagar	274	2	0	0	19.3	11.9	31.2	6.3	3.1	12.7	1.9	0.8	4.8	0	0
Punjab	Barnala	238	10	1	0	26.4	16.3	42.5	7.1	3.5	14.3	2	0.8	5	0	0
Rajasthan	Ganganagar	391	13	1	2	30.4	20	46	7.8	4.2	14.8	4.4	2	9.6	0	0
Rajasthan	Hanumangarh	341	10	0	1	29.2	19.1	44.3	7.4	3.8	14.3	4	1.8	8.9	0	0
Rajasthan	Bikaner	971	26	9	5	27.5	18.7	40.3	9.4	5.5	16	4.5	2.2	9.3	0	0
Rajasthan	Churu	439	9	4	1	25.9	16.9	39.5	9.5	5.2	17.3	3.7	1.7	8.3	0	0
Rajasthan	Jhunjhunun	385	10	7	0	27.8	18.1	42.3	12.6	7	22.7	3.4	1.5	7.7	0	0
Rajasthan	Alwar	471	12	4	4	27.4	18.1	41.3	9.3	5.1	17	5.3	2.5	11.2	0	0
Rajasthan	Bharatpur	594	17	5	5	28.8	19.3	42.6	9.3	5.2	16.8	5.5	2.6	11.6	0	0
Rajasthan	Dhaulpur	588	13	11	2	26	17.2	39	13.6	7.7	23.9	3.9	1.9	8.2	0	0
Rajasthan	Karauli	510	20	10	3	33.2	22.1	49.7	13.7	7.8	23.7	4.6	2.2	9.5	0	0
Rajasthan	Sawai Madhopur	513	20	9	2	33.3	22.2	49.7	12.8	7.2	22.6	4.1	1.9	8.7	0	0
Rajasthan	Dausa	441	7	13	3	24.2	15.7	37.2	17.7	10.1	30.7	4.8	2.3	10.2	0	0
Rajasthan	Jaipur	848	21	11	4	26.5	17.8	39.3	11.2	6.6	19.1	4.3	2.1	8.8	0	0
Rajasthan	Sikar	435	14	1	1	30.3	19.8	45.9	7.7	4.1	14.2	3.8	1.7	8.5	0	0
Rajasthan	Nagaur	384	8	3	1	26.5	17.3	40.3	9.3	5	17.4	3.9	1.8	8.6	0	0
Rajasthan	Jodhpur	874	26	10	1	29.2	19.7	43.1	10.6	6.2	18.3	3.1	1.5	6.6	0	0
Rajasthan	Jaisalmer	585	21	10	3	31.9	21.4	47.3	12.8	7.3	22.3	4.4	2.1	9.4	0	0
Rajasthan	Barmer	588	21	8	2	31.8	21.3	47.3	11.5	6.6	19.9	3.8	1.8	8.4	0	0
Rajasthan	Jalor	549	28	4	1	39	26.2	57.6	8.9	4.9	16.2	3.6	1.7	7.8	0	0
Rajasthan	Sirohi	486	22	7	4	36	24.1	53.3	11.6	6.5	20.8	5.3	2.4	11.4	0	0
Rajasthan	Pali	402	13	5	1	30.4	19.8	46.3	10.6	5.7	19.7	3.8	1.7	8.4	0	0
Rajasthan	Ajmer	844	18	1	2	24.9	16.7	37.1	6.1	3.3	11.2	3.5	1.7	7.3	0	0
Rajasthan	Tonk	403	12	2	2	29.1	19	44.3	8.5	4.5	16	4.5	2	9.7	0	0
Rajasthan	Bundi	366	9	5	1	27.4	17.7	42.1	11	5.9	20.2	3.8	1.7	8.5	0	0
Rajasthan	Bhilwara	345	16	2	1	34.9	22.8	53	8.8	4.7	16.5	3.9	1.8	8.5	0	0
Rajasthan	Rajsamand	442	15	8	3	30.6	20.3	45.9	12.8	7	23.1	4.7	2.1	10.2	0	0
Rajasthan	Dungarpur	504	13	3	1	27.6	18.2	41.7	8.6	4.7	15.7	3.6	1.7	7.8	0	0
Rajasthan	Banswara	479	11	4	3	26.7	17.5	40.4	9.4	5.2	17	4.7	2.3	9.8	0	0
Rajasthan	Chittaurgarh	288	12	2	1	32.6	21.3	49.6	9.1	4.8	17.1	4.1	1.8	9.1	0	0
Rajasthan	Kota	683	11	5	4	23.2	15.3	34.9	8.8	4.8	16	4.7	2.2	9.9	0	0
Rajasthan	Baran	417	11	2	0	27.9	18.4	42.1	8.4	4.5	15.6	3.4	1.5	7.6	0	0
Rajasthan	Jhalawar	341	14	4	3	32.5	21.3	49.3	10.5	5.6	19.6	5.1	2.3	11	0	0
Rajasthan	Udaipur	452	13	5	8	29.1	19.1	44	10.3	5.6	18.9	8.2	3.9	17	0	0
Rajasthan	Pratapgarh	473	13	5	4	28.2	18.4	42.8	10.2	5.7	17.9	5.3	2.5	11.3	0	0

Sikkim	North District	211	2	1	0	17.9	9.6	33	7.9	3.4	18.1	2.2	0.8	6.2	0	0
Sikkim	West District	200	12	5	0	26.8	14.4	49.4	11.6	5.1	26.1	2.2	0.8	6.3	0	0
Sikkim	South District	175	2	0	0	18.5	10	34.1	7.2	3.2	16.5	2.2	0.8	6.2	0	0
Sikkim	East District	419	4	3	1	16.9	9.3	30.7	8	3.6	17.8	2.3	0.8	6.6	0	0
Tamil Nadu	Thiruvallur	220	4	2	1	14.9	9.1	24.3	5.8	2.8	11.9	4	1.7	9.1	0	1
Tamil Nadu	Chennai	236	0	0	0	12.6	7.6	20.6	4.7	2.3	9.7	3.5	1.5	8	0	1
Tamil Nadu	Kancheepuram	232	1	0	4	13.1	7.9	21.6	4.7	2.3	9.6	6	2.6	13.8	0	1
Tamil Nadu	Vellore	292	4	2	2	14.5	8.9	23.5	5.6	2.7	11.6	4.3	1.9	10	0	1
Tamil Nadu	Tiruvannamalai	245	5	0	0	15.4	9.3	25.5	4.7	2.3	9.4	3.5	1.5	8.1	0	1
Tamil Nadu	Viluppuram	303	3	2	1	13.8	8.5	22.4	5.6	2.8	11.3	3.8	1.7	8.6	0	1
Tamil Nadu	Salem	286	5	0	1	15	9.1	24.4	4.6	2.3	9.4	3.8	1.7	8.7	0	1
Tamil Nadu	Namakkal	232	1	0	1	13.1	8	21.3	4.7	2.3	9.6	4	1.7	9.1	0	1
Tamil Nadu	Erode	257	0	2	1	12.5	7.5	20.6	5.8	2.9	11.4	3.9	1.7	8.9	0	1
Tamil Nadu	The Nilgiris	242	1	0	0	13.1	8	21.5	4.8	2.3	10	3.4	1.5	8	0	1
Tamil Nadu	Dindigul	268	4	2	0	14.6	8.9	23.8	5.8	2.9	11.6	3.4	1.5	7.8	0	1
Tamil Nadu	Karur	289	2	2	0	13.4	8.3	21.6	5.6	2.8	11.4	3.3	1.4	7.8	0	1
Tamil Nadu	Tiruchirappalli	244	5	6	2	15.4	9.4	25.2	8.4	4.1	17.2	4.6	2	10.4	0	0
Tamil Nadu	Perambalur	216	4	2	1	15.1	9.2	24.7	5.9	3	11.8	4	1.7	9.3	0	0
Tamil Nadu	Ariyalur	202	4	0	1	15	9.1	24.5	4.8	2.3	9.9	4.1	1.8	9.6	0	1
Tamil Nadu	Cuddalore	285	5	2	1	15	9.1	24.8	5.5	2.7	11.2	3.8	1.7	8.8	0	1
Tamil Nadu	Nagapattinam	238	4	0	4	14.8	9.1	24.1	4.7	2.3	9.6	5.9	2.6	13.4	0	0
Tamil Nadu	Thiruvarur	252	6	2	0	16	9.8	26.1	5.7	2.8	11.6	3.4	1.5	7.9	0	0
Tamil Nadu	Thanjavur	255	6	2	0	16	9.8	26.1	5.7	2.9	11.3	3.4	1.5	8	0	0
Tamil Nadu	Pudukkottai	290	3	0	1	13.9	8.6	22.6	4.6	2.2	9.5	3.9	1.7	8.9	0	1
Tamil Nadu	Sivaganga	264	4	2	0	14.4	8.9	23.5	5.7	2.9	11.2	3.4	1.5	8	0	1
Tamil Nadu	Madurai	223	4	2	0	15	9	24.8	5.8	2.8	11.8	3.5	1.5	8.2	0	1
Tamil Nadu	Theni	195	2	2	0	14	8.5	23	6	3	12.1	3.5	1.5	8.1	0	1
Tamil Nadu	Virudhunagar	191	5	0	1	15.9	9.7	26	4.8	2.3	9.9	4.1	1.8	9.4	0	1
Tamil Nadu	Ramanathapuram	244	2	1	1	13.6	8.3	22.2	5.3	2.6	10.8	4	1.7	9	0	1
Tamil Nadu	Thoothukkudi	215	1	0	3	13.4	8.2	21.8	4.8	2.3	9.9	5.1	2.2	12	0	1
Tamil Nadu	Tirunelveli	190	6	2	4	16.5	10.1	26.9	5.9	2.8	12.2	6.1	2.6	14.2	0	0
Tamil Nadu	Kanniyakumari	260	2	0	3	13.5	8.2	22.1	4.7	2.3	9.5	5.1	2.3	11.5	0	1
Tamil Nadu	Dharmapuri	252	3	2	0	14.1	8.7	23	5.7	2.8	11.7	3.5	1.5	7.9	0	1
Tamil Nadu	Krishnagiri	309	10	3	2	17.8	10.9	28.8	6.1	3.1	11.9	4.4	1.9	10.1	0	0
Tamil Nadu	Coimbatore	259	3	1	0	14.1	8.5	23.1	5.2	2.6	10.1	3.4	1.5	8	0	1
Tamil Nadu	Tiruppur	236	7	1	1	16.8	10.2	27.6	5.3	2.6	10.8	4	1.7	9	0	0
Tripura	West Tripura	469	4	6	2	13	7	24.2	11.4	5.3	24.4	2.9	1	7.9	0	0
Tripura	South Tripura	216	2	0	0	13.8	7.3	26.2	8.8	3.9	19.6	2.5	0.9	7.1	0	0
Tripura	Dhalai	290	3	4	0	13.7	7.2	25.9	11.6	5.4	24.5	2.4	0.8	6.7	0	0
Tripura	North Tripura	355	9	8	1	16.5	8.8	30.8	14.7	7	31	2.7	1	7.4	0	0
Uttar Pradesh	Saharanpur	1041	35	26	4	36.6	25.9	51.5	22	14.4	33.4	4.9	2.5	9.4	0	0
Uttar Pradesh	Muzaffarnagar	564	26	4	0	44.2	30.2	64.1	11.7	6.9	20	4	1.9	8.5	0	0
Uttar Pradesh	Bijnor	496	20	8	1	41.4	28.2	60.3	16	9.2	27.7	4.7	2.3	9.8	0	0
Uttar Pradesh	Moradabad	1042	52	18	7	47.1	33.7	65.5	16.7	10.6	26.1	6.2	3.3	11.6	0	0
Uttar Pradesh	Rampur	535	18	10	2	38.1	26	55.3	17.3	10.3	28.8	5.1	2.4	10.4	0	0
Uttar Pradesh	Jyotiba Phule Nagar	543	24	9	5	43.2	29.8	62.4	16.3	9.8	27.1	7	3.5	13.8	0	0
Uttar Pradesh	Meerut	1350	54	20	4	40.6	29.3	56	15.2	9.8	23.5	4.3	2.2	8.4	0	0
Uttar Pradesh	Baghpat	450	20	7	1	43.3	29.3	63.5	15.7	9.1	26.9	4.9	2.3	10.3	0	0
Uttar Pradesh	Ghaziabad	980	32	24	5	36.3	25.3	51.6	21.6	14	33.4	5.5	2.8	10.5	0	0
Uttar Pradesh	Gautam Buddha Nagar	1131	43	24	7	39.3	28	55	19.2	12.4	29.8	6.1	3.2	11.3	0	0
Uttar Pradesh	Bulandshahr	564	21	8	3	39.5	27.1	57.4	14.9	8.7	25.3	5.7	2.9	11.4	0	0
Uttar Pradesh	Aligarh	1112	59	29	5	49.7	35.6	69.1	23	15.2	34.8	5.2	2.7	9.9	0	0
Uttar Pradesh	Mahamaya Nagar	525	24	13	3	44.3	30.5	64	20.2	12.4	33	5.8	2.8	11.7	0	0
Uttar Pradesh	Mathura	573	35	10	3	52.7	36.8	75.1	16.5	9.8	27.5	5.5	2.7	11.3	0	0
Uttar Pradesh	Agra	1082	43	25	7	40.7	29	56.8	20.7	13.6	31.4	6	3.3	11.2	0	0

Uttar Pradesh	Firozabad	1038	52	17	10	47.4	34.1	65.5	16.1	10.2	25.4	7.7	4.2	14.2	0	0
Uttar Pradesh	Mainpuri	458	26	8	6	48.9	33.8	70.3	16.6	9.6	28.4	8.2	4.1	16.5	0	0
Uttar Pradesh	Budaun	637	40	7	5	53.9	37.7	76.4	13.4	8	22.5	6.4	3.2	12.7	0	0
Uttar Pradesh	Bareilly	934	30	21	7	36.2	25.2	51.7	20.1	12.8	31.4	6.5	3.4	12.4	0	0
Uttar Pradesh	Pilibhit	506	19	8	6	39.8	27	58.2	15.9	9.5	26.7	7.9	4	15.3	0	0
Uttar Pradesh	Shahjahanpur	546	34	13	3	52.8	36.4	76.1	19.9	12.2	32.5	5.7	2.8	11.6	0	0
Uttar Pradesh	Kheri	550	25	9	3	43.9	30.1	63.7	15.9	9.4	26.7	5.7	2.9	11.3	0	0
Uttar Pradesh	Sitapur	493	35	13	10	57	39.6	81.5	21.2	12.8	35.1	11.5	6	21.7	0	0
Uttar Pradesh	Hardoi	471	33	5	9	55.9	38.9	79.6	13.7	7.9	23.7	10.7	5.4	21	0	0
Uttar Pradesh	Unnao	439	17	7	0	40.6	27.3	60.1	15.9	9.2	27.5	4.4	2	9.4	0	0
Uttar Pradesh	Lucknow	515	18	7	1	38.6	26.3	56.4	14.5	8.3	24.9	4.7	2.3	9.7	0	0
Uttar Pradesh	Rae Bareli	387	15	9	1	40.6	27.4	59.7	19	11.3	32	4.9	2.3	10.5	0	0
Uttar Pradesh	Farrukhabad	554	33	20	4	51.6	36.1	73.2	26.9	16.7	43	6.3	3.2	12.5	0	0
Uttar Pradesh	Kannauj	537	25	16	4	44.6	30.5	64.7	23.1	14.2	37.3	6.4	3.1	12.8	0	0
Uttar Pradesh	Etawah	478	14	7	2	36.2	24.4	53.3	15.5	9	26.5	5.3	2.5	11.1	0	0
Uttar Pradesh	Auraiya	402	12	3	2	37.5	25.1	55.7	12.6	6.9	22.9	5.6	2.7	11.7	0	0
Uttar Pradesh	Kanpur Dehat	395	15	5	4	40.5	27.4	59.6	14.6	8.2	25.8	7	3.5	14.1	0	0
Uttar Pradesh	Kanpur Nagar	561	13	4	1	33	22.3	48.5	12	6.9	20.7	4.6	2.2	9.6	0	0
Uttar Pradesh	Jalaun	294	17	4	1	48.1	32.5	70.6	15.2	8.4	27.2	5.4	2.5	11.4	0	0
Uttar Pradesh	Jhansi	604	21	9	3	38.3	26.3	55.5	15.3	9.2	25.3	5.5	2.7	11.3	0	0
Uttar Pradesh	Lalitpur	345	12	6	7	39.4	26.3	58.6	16.6	9.4	29.3	10.1	5	20.5	0	0
Uttar Pradesh	Hamirpur	312	10	4	0	38.4	25.4	57.7	14.9	8.3	26.6	4.6	2.2	9.7	0	0
Uttar Pradesh	Mahoba	364	11	6	1	37.5	24.9	56.1	16.4	9.5	28.3	5.2	2.5	11	0	0
Uttar Pradesh	Banda	304	11	5	3	40.6	26.6	61.6	16.1	9	28.4	6.7	3.2	14.3	0	0
Uttar Pradesh	Chitrakoot	538	22	12	3	41.4	28.3	60.1	19	11.4	31.6	5.7	2.8	11.8	0	0
Uttar Pradesh	Fatehpur	312	9	2	2	37.5	24.7	56.5	12.9	7	23.8	6	2.8	12.6	0	0
Uttar Pradesh	Pratapgarh	405	13	7	4	37.9	25.4	56.2	16.6	9.5	28.8	6.9	3.4	14.1	0	0
Uttar Pradesh	Kaushambi	505	33	13	5	54.4	37.8	77.6	21	12.5	34.8	7.2	3.6	14.5	0	0
Uttar Pradesh	Allahabad	488	14	9	5	35.8	24	53	17.1	10	29	7.2	3.6	14.6	0	0
Uttar Pradesh	Bara Banki	427	9	12	2	33.6	22.3	50.3	21.8	12.9	36.4	5.6	2.7	11.6	0	0
Uttar Pradesh	Faizabad	483	24	10	2	45.8	31.3	66.5	18.4	10.8	30.9	5.4	2.6	11	0	0
Uttar Pradesh	Ambedkar Nagar	505	26	3	2	46.9	32.4	67.6	11.6	6.6	20.2	5.2	2.5	10.9	0	0
Uttar Pradesh	Sultanpur	445	24	10	5	47.9	32.8	69.4	18.8	11	31.8	7.6	3.7	15.5	0	0
Uttar Pradesh	Bahraich	665	42	10	2	54.4	38.3	76.7	15.2	9.1	25.4	4.8	2.3	9.9	0	0
Uttar Pradesh	Shrawasti	716	38	19	11	48.7	34.4	68.5	21.9	13.7	34.9	10.3	5.6	18.9	0	0
Uttar Pradesh	Balrampur	663	31	13	6	45	31.2	64.3	17.9	11	29	7.1	3.6	14.1	0	0
Uttar Pradesh	Gonda	639	50	7	8	63.9	45.3	89.3	13.3	7.8	22.7	8.5	4.4	16.3	0	0
Uttar Pradesh	Siddharth Nagar	678	33	10	9	45.9	31.9	65.5	15.1	9.1	25.1	8.9	4.7	16.9	0	0
Uttar Pradesh	Basti	584	19	7	7	37.5	25.5	54.6	13.9	8.2	23.5	8.1	4.1	15.8	0	0
Uttar Pradesh	Sant Kabir Nagar	610	23	8	6	39.7	27.4	57.3	14.6	8.7	24.2	7.3	3.8	14	0	0
Uttar Pradesh	Mahrajganj	591	36	5	3	52.6	36.6	74.9	12.3	7.1	21.2	5.5	2.7	11	0	0
Uttar Pradesh	Gorakhpur	471	17	7	4	39.5	26.7	57.9	15.4	9	26.3	6.8	3.3	13.8	0	0
Uttar Pradesh	Kushinagar	615	22	10	4	38.8	26.6	56.2	16	9.6	26.8	6.2	3.1	12.1	0	0
Uttar Pradesh	Deoria	491	16	4	4	37.5	25.4	55.1	12.3	7.1	21.3	6.6	3.2	13.3	0	0
Uttar Pradesh	Azamgarh	477	17	5	0	39.2	26.5	57.6	13.7	8	23.5	4.2	2	8.9	0	0
Uttar Pradesh	Mau	527	21	8	5	41.1	28	59.9	15.5	9.2	26.2	7.1	3.6	13.9	0	0
Uttar Pradesh	Ballia	531	15	7	3	35.6	23.9	52.6	14.7	8.6	24.8	5.8	2.8	11.8	0	0
Uttar Pradesh	Jaunpur	528	23	13	0	43	29.6	62.1	20.5	12.4	33.6	4	1.9	8.3	0	0
Uttar Pradesh	Ghazipur	495	19	9	4	40.4	27.6	58.8	16.9	10	28.4	6.5	3.3	13	0	0
Uttar Pradesh	Chandauli	582	15	8	0	34	23.1	49.8	15	8.8	25.3	4	1.9	8.2	0	0
Uttar Pradesh	Varanasi	864	29	6	2	36.9	25.9	52.3	11	6.5	18.4	4.4	2.2	8.7	0	0
Uttar Pradesh	Sant Ravidas Nagar (Bhadohi)	643	30	10	3	44.8	31.1	64.2	15.9	9.5	26.4	5.5	2.7	10.9	0	0
Uttar Pradesh	Mirzapur	510	30	8	5	50.5	34.9	72.5	15.8	9.2	26.9	7	3.6	13.8	0	0
Uttar Pradesh	Sonbhadra	493	21	1	3	42.2	28.9	61.3	10	5.5	18	6	2.9	12.2	0	0
Uttar Pradesh	Etah	546	26	10	1	45	31	64.9	17	10	28.7	4.6	2.2	9.6	0	0

Uttar Pradesh	Kanshiram Nagar	612	36	19	3	51.6	36.2	73.1	24.4	15.2	38.9	5.4	2.7	10.9	0	0
Uttarakhand	Uttarkashi	308	10	7	0	27.9	17.6	44	13.9	7.2	26.5	2.4	1	6.1	0	0
Uttarakhand	Chamoli	300	7	7	1	25.1	15.8	39.6	14.1	7.4	26.8	2.7	1.1	6.6	0	0
Uttarakhand	Rudraprayag	269	3	2	0	22.4	13.9	35.8	9.8	5	19.3	2.5	1	6	0	0
Uttarakhand	Tehri Garhwal	355	6	5	2	23.2	14.6	36.8	11.6	6.1	22.2	3.1	1.3	7.5	0	0
Uttarakhand	Dehradun	547	15	3	1	26.4	16.9	40.9	8.7	4.6	16.4	2.5	1.1	5.7	0	0
Uttarakhand	Garhwal	266	3	1	0	22.5	13.8	36.5	9.1	4.6	17.9	2.4	1	5.9	0	0
Uttarakhand	Pithoragarh	274	11	5	1	29.9	18.5	47.7	12.6	6.5	23.9	2.8	1.1	6.9	0	0
Uttarakhand	Bageshwar	348	7	1	0	24	15	38.1	8.4	4.3	16.6	2.3	1	5.7	0	0
Uttarakhand	Almora	319	9	1	0	26.5	16.6	42	8.6	4.4	16.7	2.4	1	5.8	0	0
Uttarakhand	Champawat	348	12	4	1	28.3	17.7	45.2	10.8	5.6	21	2.7	1.1	6.6	0	0
Uttarakhand	Nainital	687	17	7	3	25.5	16.4	39.3	10.3	5.7	18.7	3.1	1.3	7.1	0	0
Uttarakhand	Udham Singh Nagar	813	25	9	4	28.3	18.4	43.2	10.7	5.9	19.4	3.2	1.4	7.3	0	0
Uttarakhand	Hardwar	991	35	16	4	31.2	20.5	47.1	13.5	7.7	23.7	3.1	1.3	7	0	0
West Bengal	Darjiling	204	4	0	0	22	13.5	35.6	4.9	2.3	10.2	2.2	0.9	5.4	0	0
West Bengal	Jalpaiguri	246	3	0	2	20.4	12.6	32.9	4.8	2.3	10	2.9	1.1	7.1	0	0
West Bengal	Koch Bihar	260	8	4	0	24.1	14.9	38.7	7.1	3.5	14.3	2.1	0.9	5.2	0	0
West Bengal	Uttar Dinajpur	459	16	2	1	26.6	17	41.4	5.3	2.7	10.6	2.3	0.9	5.6	0	0
West Bengal	Dakshin Dinajpur	230	9	2	0	25.6	15.9	40.9	6	2.9	12.3	2.1	0.8	5.5	0	0
West Bengal	Maldah	386	9	2	4	22.4	14.1	35.3	5.5	2.6	11.3	3.5	1.4	8.6	0	0
West Bengal	Murshidabad	374	9	1	1	22.7	14.2	36.1	5	2.4	10.3	2.3	0.9	5.8	0	0
West Bengal	Birbhum	270	6	2	0	22.3	13.8	35.9	5.8	2.9	11.9	2.1	0.9	5.3	0	0
West Bengal	Barddhaman	252	3	3	0	20.3	12.5	32.8	6.5	3.2	13.2	2.1	0.8	5.3	0	0
West Bengal	Nadia	255	4	3	0	20.9	12.8	33.9	6.5	3.2	13.2	2.1	0.8	5.2	0	0
West Bengal	North Twenty Four Parganas	234	4	1	0	21.4	13.3	34.3	5.4	2.6	10.9	2.1	0.8	5.3	0	0
West Bengal	Hugli	205	3	1	0	21	13	33.8	5.5	2.7	11.3	2.2	0.9	5.4	0	0
West Bengal	Bankura	273	10	0	1	25.5	16	40.6	4.8	2.3	9.8	2.4	1	6	0	0
West Bengal	Puruliya	386	11	1	0	24.2	15.2	38.2	5	2.4	10.1	2	0.8	4.9	0	0
West Bengal	Haora	247	5	1	0	22	13.5	35.5	5.3	2.6	11	2.1	0.9	5.2	0	0
West Bengal	Kolkata	174	2	1	0	20.8	12.7	33.9	5.6	2.7	11.6	2.2	0.9	5.4	0	0
West Bengal	South Twenty Four Parganas	341	8	0	3	22.8	14.2	36.5	4.6	2.2	9.6	3.1	1.2	7.6	0	0
West Bengal	Paschim Medinipur	258	7	1	0	23.9	14.9	38.1	5.4	2.6	11	2.1	0.8	5.4	0	0
West Bengal	Purba Medinipur	274	4	3	0	20.5	12.6	33.1	6.3	3.1	12.9	2.1	0.8	5.3	0	0
Telangana	Adilabad	247	6	2	0	22.5	13.4	37.4	7.4	3.5	15.7	1.9	0.7	5.2	0	0
Telangana	Nizamabad	254	11	2	1	27.1	16.2	45.1	7.3	3.5	15.2	2.2	0.8	5.9	0	0
Telangana	Karimnagar	201	7	1	0	24.7	14.7	41.3	6.9	3.3	14.5	1.9	0.7	5.4	0	0
Telangana	Medak	244	9	5	0	25.5	15.3	42.3	9.7	4.6	20.4	1.9	0.7	5	0	0
Telangana	Hyderabad	280	1	0	1	18.3	10.8	30.9	6.1	2.8	13.2	2.2	0.8	5.9	0	0
Telangana	Rangareddy	236	4	1	0	21.3	12.6	35.8	6.9	3.2	14.6	1.9	0.7	5.2	0	0
Telangana	Mahbubnagar	301	7	5	0	22.4	13.5	36.9	9.3	4.5	19.3	1.9	0.7	5.1	0	0
Telangana	Nalgonda	243	4	0	0	21.1	12.6	35	6.2	2.9	13.2	1.9	0.7	5.2	0	0
Telangana	Warangal	192	3	1	0	21.4	12.8	35.5	6.9	3.3	14.4	1.9	0.7	5.4	0	0
Telangana	Khammam	229	5	1	1	22.2	13.3	36.9	6.7	3.2	13.9	2.2	0.8	5.8	0	0

Table S2. District-specific data including the precision-weighted probability of neonatal mortality (NM), post-neonatal mortality (PNM), and child mortality (CM) (per 1,000 live births), stratified by child's sex and place of residence, NFHS 4

State_Name	District_Name	Stratified by child's sex						Stratified by place of residence					
		Boys			Girls			Urban			Rural		
		NM	PNM	CM	NM	PNM	CM	NM	PNM	CM	NM	PNM	CM
Andaman And Nicobar Islands	Nicobars	11.03	7.43	2.91	14.22	6.04	2.55	.	.	.	11.82	6.67	2.34
Andaman And Nicobar Islands	North & Middle Andaman	11.42	6.65	2.91	13.48	6.10	2.58	12.70	5.63	2.63	11.69	6.32	2.40
Andaman And Nicobar Islands	South Andaman	10.90	6.60	2.85	13.93	6.96	2.63	11.71	4.78	2.65	12.33	7.34	2.53
Andhra Pradesh	Srikakulam	32.96	7.15	2.52	18.76	7.96	2.61	14.10	5.49	2.66	32.24	8.58	2.21
Andhra Pradesh	Vizianagaram	27.73	7.97	2.51	16.84	8.02	2.61	14.03	5.33	2.68	25.27	9.16	2.22
Andhra Pradesh	Visakhapatnam	31.73	8.40	2.51	16.04	8.23	2.65	13.54	4.84	2.65	28.08	10.07	2.32
Andhra Pradesh	East Godavari	28.36	10.38	2.54	17.45	9.19	2.66	14.72	5.09	2.65	26.02	12.13	2.21
Andhra Pradesh	West Godavari	29.97	7.54	2.54	16.70	8.44	2.67	14.16	5.43	2.69	26.33	8.91	2.28
Andhra Pradesh	Krishna	27.31	7.21	2.92	15.97	9.58	2.67	13.99	4.95	2.66	23.69	10.29	2.65
Andhra Pradesh	Guntur	28.14	11.71	2.48	16.83	7.43	2.70	14.48	7.79	2.62	25.75	10.45	2.29
Andhra Pradesh	Prakasam	35.56	8.76	2.44	17.92	8.82	3.04	14.74	8.87	2.68	32.53	8.94	2.49
Andhra Pradesh	Sri Potti Sriramulu Nellore	33.05	7.40	2.51	16.79	7.16	2.62	14.28	5.11	2.65	28.84	7.89	2.25
Andhra Pradesh	Y.S.R.	35.33	7.84	2.47	17.64	7.11	2.66	15.72	5.86	2.62	31.81	8.26	2.31
Andhra Pradesh	Kurnool	28.95	8.48	2.42	17.12	7.88	3.33	13.96	4.85	2.72	25.78	9.47	2.45
Andhra Pradesh	Anantapur	30.63	7.25	2.53	16.40	8.97	3.03	14.48	4.92	2.64	26.21	9.75	2.62
Andhra Pradesh	Chittoor	26.58	7.36	2.88	15.90	7.19	2.66	13.69	5.06	2.73	22.49	8.08	2.25
Arunachal Pradesh	Tawang	9.08	10.22	5.55	10.80	6.79	3.90	8.53	5.85	3.11	9.82	9.06	5.78
Arunachal Pradesh	West Kameng	9.38	10.97	5.00	10.36	6.95	3.40	8.63	5.82	3.12	9.71	9.48	4.40
Arunachal Pradesh	East Kameng	9.94	13.77	5.42	11.30	9.90	4.25	8.32	10.79	3.10	11.89	12.15	5.90
Arunachal Pradesh	Papumpare	9.14	9.72	4.99	10.70	7.53	3.12	8.99	6.69	3.02	9.56	9.21	4.56
Arunachal Pradesh	Upper Subansiri	8.79	9.36	5.46	10.19	7.40	3.41	8.61	5.77	3.15	9.06	9.11	4.77
Arunachal Pradesh	West Siang	9.10	10.27	5.76	10.39	7.03	3.85	8.67	6.05	3.16	9.52	9.05	5.83
Arunachal Pradesh	East Siang	9.21	10.55	5.12	10.40	7.01	3.42	8.47	5.91	3.10	9.62	9.29	4.56
Arunachal Pradesh	Upper Siang	9.39	9.55	5.25	10.42	7.13	3.51	8.73	6.00	3.08	9.62	8.59	4.68
Arunachal Pradesh	Changlang	8.92	9.72	5.00	11.36	8.45	3.81	8.62	6.10	3.09	10.25	10.02	4.86
Arunachal Pradesh	Tirap	9.85	13.08	6.30	10.95	7.67	3.44	9.01	5.92	3.10	10.32	11.49	5.42
Arunachal Pradesh	Lower Subansiri	9.81	9.25	5.12	10.37	7.11	3.99	8.74	6.09	3.07	9.87	8.35	5.23
Arunachal Pradesh	Kurung Kumey	9.23	10.27	8.68	10.55	7.84	3.70	8.72	6.02	3.11	9.71	9.71	7.47
Arunachal Pradesh	Dibang Valley	9.24	10.40	6.79	10.31	7.58	3.87	8.61	7.83	3.29	9.56	9.03	5.27
Arunachal Pradesh	Lower Dibang Valley	9.69	13.24	8.06	10.65	7.58	3.85	8.92	5.73	3.21	10.06	12.21	6.32
Arunachal Pradesh	Lohit	11.45	11.39	5.44	12.64	9.16	3.77	8.50	7.05	3.19	15.20	11.19	4.69
Arunachal Pradesh	Anjaw	10.34	9.95	7.47	10.60	9.64	3.38	8.77	6.11	3.17	11.07	11.09	5.51
Assam	Kokrajhar	37.67	10.61	3.64	24.74	11.13	4.70	18.18	7.13	2.87	32.54	11.34	4.43
Assam	Dhubri	33.88	10.84	3.50	22.40	11.28	4.02	18.91	12.29	2.93	26.51	10.30	3.69
Assam	Goalpara	34.25	10.90	3.49	25.51	11.27	4.51	17.90	6.94	2.96	30.72	11.85	4.14
Assam	Barpeta	33.22	11.13	3.48	26.03	12.81	3.53	18.25	7.09	2.94	30.73	13.01	3.33
Assam	Morigaon	48.36	16.45	3.94	27.54	20.38	3.91	18.75	12.18	2.92	43.65	20.09	4.12
Assam	Nagaon	36.35	13.84	3.56	31.47	9.36	4.08	18.08	9.05	2.90	40.77	11.52	3.73
Assam	Sonitpur	37.05	12.59	3.63	24.35	11.20	3.69	18.32	7.18	2.94	31.21	12.52	3.53
Assam	Lakhimpur	36.42	11.77	4.60	23.76	10.75	5.19	18.28	7.01	2.91	30.40	12.02	6.05
Assam	Dhemaji	43.93	10.22	3.54	25.69	9.45	4.00	18.10	7.20	2.91	37.97	9.86	3.82
Assam	Tinsukia	36.43	12.88	4.57	26.04	9.78	4.59	19.19	8.42	2.92	32.33	11.51	5.58
Assam	Dibrugarh	29.83	12.21	4.18	23.36	8.88	4.18	17.92	8.66	2.97	25.51	10.19	4.01
Assam	Sivasagar	36.26	15.14	4.14	26.33	12.08	3.64	18.15	7.22	2.92	33.84	15.57	4.02
Assam	Jorhat	37.06	11.06	3.55	23.89	9.13	4.22	17.66	6.97	2.90	32.10	10.41	4.07
Assam	Golaghat	42.32	12.23	4.12	27.28	9.78	3.62	18.10	7.04	2.93	39.45	11.52	3.91
Assam	Karbi Anglong	42.11	13.71	5.76	24.95	13.16	3.63	19.74	8.68	2.96	35.03	14.47	4.87
Assam	Dima Hasao	36.57	12.65	3.95	26.20	9.30	5.10	21.59	6.31	2.89	30.59	12.27	5.59
Assam	Cachar	35.81	17.35	3.60	29.08	13.11	4.09	18.07	7.03	2.92	36.85	17.93	3.94

Assam	Karimganj	38.09	18.62	4.96	30.14	13.45	3.52	19.01	9.48	2.95	37.71	17.32	4.00
Assam	Hailakandi	38.50	12.47	4.50	27.43	11.35	3.48	20.20	7.26	2.92	34.79	12.90	4.15
Assam	Bongaigaon	32.00	10.45	4.58	22.99	9.66	3.64	17.99	7.15	2.92	26.51	10.34	4.34
Assam	Chirang	31.46	13.26	4.66	25.95	8.55	4.01	18.27	7.26	2.93	29.57	10.84	4.76
Assam	Kamrup	34.31	9.83	4.67	23.19	8.68	3.63	18.05	6.94	2.99	28.21	9.05	3.91
Assam	Kamrup Metropolitan	35.05	11.32	3.68	26.63	9.49	4.86	19.88	5.61	2.96	33.81	12.69	4.57
Assam	Nalbari	38.58	13.37	3.68	25.57	13.66	3.61	18.15	7.01	2.90	34.52	15.59	3.49
Assam	Baksa	30.76	15.73	4.02	25.02	9.64	3.65	18.27	7.35	2.90	27.52	13.38	3.80
Assam	Darrang	38.40	11.33	3.53	24.58	9.31	3.55	18.30	7.17	2.91	33.12	10.74	3.32
Assam	Udalguri	34.73	11.99	3.57	26.05	12.07	3.63	18.44	7.15	2.92	32.05	12.71	3.42
Bihar	Pashchim Champaran	46.32	10.05	3.42	31.40	8.34	8.00	25.74	5.48	2.65	42.74	9.64	6.75
Bihar	Purba Champaran	35.20	11.40	3.25	30.25	17.99	4.00	27.42	5.20	2.60	31.78	17.63	3.52
Bihar	Sheohar	47.43	11.47	4.21	30.27	11.55	5.13	27.56	7.47	2.60	40.57	12.23	5.25
Bihar	Sitamarhi	45.86	9.17	4.63	32.14	9.80	6.25	25.48	5.31	2.70	43.03	9.92	6.46
Bihar	Madhubani	37.66	7.56	2.96	30.94	13.90	7.07	30.04	7.34	2.60	32.64	10.83	5.54
Bihar	Supaul	35.46	7.71	2.88	29.76	10.04	4.92	27.97	6.92	2.61	31.60	8.78	3.87
Bihar	Araria	50.04	8.04	5.29	31.83	9.49	5.51	25.77	5.48	2.60	44.19	8.98	6.71
Bihar	Kishanganj	40.89	9.84	2.97	30.51	7.93	4.50	31.44	5.22	2.60	34.17	9.28	3.63
Bihar	Purnia	48.67	7.20	5.20	36.81	16.62	5.57	31.48	5.26	2.66	46.93	12.83	6.29
Bihar	Katihar	51.88	9.10	3.35	29.40	8.30	4.64	26.92	5.44	2.62	43.31	9.07	4.05
Bihar	Madhepura	42.61	8.32	3.24	29.39	8.45	6.09	26.12	5.45	2.64	37.19	8.53	4.99
Bihar	Saharsa	48.82	9.40	4.60	33.59	15.39	4.90	25.69	5.50	2.63	45.91	13.92	5.61
Bihar	Darbhanga	36.51	8.23	3.69	30.45	8.78	4.61	28.30	5.16	2.61	33.07	8.86	4.45
Bihar	Muzaffarpur	37.44	7.95	3.01	32.49	8.51	4.78	25.75	5.68	2.63	37.18	8.23	3.83
Bihar	Gopalganj	40.38	9.56	4.30	27.23	10.03	4.09	28.77	5.65	2.64	31.85	10.49	4.66
Bihar	Siwan	44.10	8.34	3.04	30.71	10.06	5.63	28.59	5.56	2.60	37.51	9.79	4.56
Bihar	Saran	38.07	14.46	2.97	28.51	9.19	4.15	25.88	5.54	2.65	32.88	13.41	3.26
Bihar	Vaishali	39.32	10.56	3.27	29.57	11.67	5.11	27.35	7.19	2.61	34.42	11.52	4.40
Bihar	Samastipur	33.08	11.15	2.95	31.30	8.76	4.50	27.57	5.73	2.62	31.51	10.66	3.58
Bihar	Begusarai	39.70	8.20	3.72	28.32	10.38	4.00	28.59	6.13	2.59	32.89	9.67	4.12
Bihar	Khagaria	44.34	10.40	2.87	29.98	8.58	4.94	27.79	5.70	2.60	37.53	9.91	3.83
Bihar	Bhagalpur	35.73	12.39	3.26	26.01	11.46	4.47	26.02	7.76	2.60	29.35	12.77	4.13
Bihar	Banka	37.09	6.69	3.38	32.72	13.39	4.63	31.05	5.47	2.61	34.15	10.01	4.02
Bihar	Munger	39.02	7.83	3.04	33.38	9.38	4.76	24.60	6.08	2.59	42.42	8.75	4.02
Bihar	Lakhisarai	40.40	9.01	2.93	29.99	13.61	5.09	25.39	6.39	2.59	37.18	12.39	4.03
Bihar	Sheikhpura	36.72	9.70	2.96	32.82	9.23	6.63	28.14	6.29	2.58	35.57	10.09	5.39
Bihar	Nalanda	34.36	12.31	3.43	34.08	9.37	4.67	26.74	5.06	2.65	35.74	12.36	3.82
Bihar	Patna	38.78	9.71	3.08	27.19	8.22	4.09	23.33	5.81	2.61	37.14	9.98	3.55
Bihar	Bhojpur	36.77	8.28	3.73	30.99	8.23	4.16	24.57	5.09	2.60	36.00	8.77	4.16
Bihar	Buxar	48.81	9.26	3.80	31.28	8.96	4.52	29.51	5.51	2.61	40.82	9.46	4.44
Bihar	Kaimur (Bhabua)	43.67	7.50	2.97	29.11	13.93	5.80	29.69	5.58	2.62	35.35	11.41	4.40
Bihar	Rohtas	48.26	10.03	3.79	31.56	8.32	5.17	30.62	6.51	2.63	41.31	9.17	5.16
Bihar	Aurangabad	34.53	11.46	3.42	31.83	9.33	4.77	28.79	7.29	2.69	32.13	10.82	3.77
Bihar	Gaya	49.69	10.16	3.26	32.52	8.40	5.38	26.75	6.60	2.60	45.30	9.48	4.69
Bihar	Nawada	33.33	9.47	3.03	32.12	7.35	4.61	25.19	5.33	2.59	34.25	8.58	3.70
Bihar	Jamui	43.75	9.40	3.73	34.17	7.24	4.43	29.38	5.33	2.69	41.44	8.40	3.88
Bihar	Jehanabad	45.55	9.39	3.43	32.80	12.73	4.21	28.37	6.99	2.61	41.67	11.54	3.77
Bihar	Arwal	35.08	9.01	3.33	31.12	10.75	4.06	25.52	5.40	2.60	33.78	10.77	3.60
Chandigarh	Chandigarh	21.04	8.98	3.11	24.72	6.74	2.84	24.02	5.93	2.62	21.35	9.80	3.30
Chhattisgarh	Korea (Koriya)	45.23	9.77	4.40	38.19	9.69	4.11	37.90	5.60	2.73	43.34	12.09	4.98
Chhattisgarh	Surguja	43.75	11.05	4.92	35.39	18.09	4.58	37.28	12.23	2.72	39.21	15.52	5.74
Chhattisgarh	Jashpur	48.14	10.77	4.85	36.00	8.48	4.62	36.71	5.54	2.67	43.26	10.94	5.76
Chhattisgarh	Raigarh	41.90	9.18	3.91	34.24	11.09	4.71	33.85	5.52	2.71	38.98	12.06	4.90
Chhattisgarh	Korba	51.15	8.16	3.46	40.38	14.13	5.09	41.21	9.06	2.66	48.94	11.73	5.34
Chhattisgarh	Janjgir - Champa	36.82	7.28	4.35	39.09	7.94	4.16	37.90	5.30	2.72	37.63	8.03	4.81

Chhattisgarh	Bilaspur	43.04	7.50	4.20	37.17	8.74	4.40	37.64	4.90	2.71	40.87	9.09	4.88
Chhattisgarh	Kabirdham	42.72	8.57	4.27	35.34	8.46	5.67	37.20	5.41	2.80	38.64	9.34	5.63
Chhattisgarh	Rajnandgaon	46.33	11.07	4.14	30.78	8.73	3.81	39.97	4.77	2.71	34.38	11.76	4.32
Chhattisgarh	Durg	45.44	6.69	4.28	33.67	8.34	4.74	34.47	4.79	2.71	41.46	8.78	5.73
Chhattisgarh	Raipur	51.55	8.14	3.93	35.20	9.63	4.62	34.11	4.69	2.81	50.16	11.28	4.38
Chhattisgarh	Mahasamund	44.97	7.18	4.99	33.96	9.84	4.00	34.48	5.55	2.69	41.05	9.05	5.23
Chhattisgarh	Dhamtari	41.97	7.38	3.85	37.66	8.43	4.57	35.76	5.39	2.69	41.58	8.73	4.74
Chhattisgarh	Uttar Bastar Kanker	51.64	7.14	4.34	41.94	8.85	4.11	39.28	5.49	2.77	51.93	8.68	4.78
Chhattisgarh	Bastar	45.11	11.95	4.85	38.10	8.33	6.30	36.31	5.37	2.82	43.75	11.62	6.92
Chhattisgarh	Narayanpur	44.96	7.65	5.06	32.15	8.69	3.92	35.40	6.69	2.70	37.71	8.42	5.09
Chhattisgarh	Dakshin Bastar Dantewada	57.63	13.43	6.17	40.33	9.47	6.56	38.12	7.05	2.71	54.71	12.85	9.55
Chhattisgarh	Bijapur	52.55	7.44	3.66	33.94	8.68	3.87	36.57	5.32	2.72	44.86	8.59	3.73
Dadra And Nagar Haveli	Dadra & Nagar Haveli	17.50	16.41	2.97	13.90	9.36	3.78	10.85	8.64	2.62	18.09	15.84	3.80
Daman And Diu	Diu	21.24	8.88	3.02	18.55	6.79	2.66	20.88	4.76	2.62	17.50	10.04	2.89
Daman And Diu	Daman	23.10	9.04	2.97	19.61	7.73	2.73	24.56	5.95	2.64	17.09	11.02	2.94
Goa	North Goa	23.17	6.43	2.94	13.28	5.81	2.71	17.38	4.24	2.60	16.74	7.06	2.87
Goa	South Goa	20.53	6.53	2.97	13.77	5.92	2.71	17.22	4.34	2.62	15.20	7.09	2.95
Gujarat	Kachchh	32.21	5.88	4.03	22.14	8.07	2.80	28.54	3.64	2.38	25.81	8.37	4.76
Gujarat	Banaskantha	37.45	8.13	3.54	22.97	7.78	2.81	23.38	3.11	2.45	34.93	10.17	3.77
Gujarat	Patan	33.03	7.32	3.47	21.20	7.24	2.86	25.72	3.03	2.39	27.05	9.27	3.91
Gujarat	Mahesana	25.99	8.44	4.15	21.68	7.53	3.30	23.47	3.17	2.37	23.68	10.35	5.18
Gujarat	Sabarkantha	26.57	6.52	4.04	19.03	7.29	2.87	24.63	3.08	2.37	20.41	8.35	4.39
Gujarat	Gandhinagar	29.66	6.66	3.64	21.23	7.21	3.72	25.12	2.83	2.40	24.53	9.32	5.51
Gujarat	Ahmadabad	28.95	6.10	3.63	20.45	7.63	3.36	22.91	2.88	2.37	25.93	9.19	5.07
Gujarat	Surendranagar	26.04	7.50	4.03	19.77	10.11	3.26	21.96	3.08	2.37	22.16	11.81	5.05
Gujarat	Rajkot	26.47	6.69	4.19	22.37	7.48	3.29	24.69	3.86	2.51	24.34	8.74	4.35
Gujarat	Jamnagar	30.12	6.04	3.60	21.39	8.14	2.86	23.89	3.67	2.36	26.19	8.86	4.29
Gujarat	Porbandar	25.37	6.49	3.52	20.47	9.23	2.91	22.67	3.86	2.37	22.52	9.79	4.17
Gujarat	Junagadh	24.81	6.75	3.60	20.21	8.18	3.23	21.29	2.87	2.38	22.33	10.28	4.76
Gujarat	Amreli	25.93	6.11	3.69	20.38	7.63	2.98	22.17	3.07	2.38	23.41	8.58	4.21
Gujarat	Bhavnagar	30.43	5.89	3.55	20.68	7.40	2.83	24.71	2.93	2.36	25.08	8.26	4.20
Gujarat	Anand	32.79	5.91	4.03	21.87	9.35	2.91	22.04	3.05	2.40	32.61	10.01	4.75
Gujarat	Kheda	35.46	5.84	3.51	22.92	12.29	3.19	24.61	3.95	2.44	33.41	11.21	3.99
Gujarat	Panchmahal	25.33	5.82	3.53	24.01	8.03	2.85	22.85	3.25	2.37	26.90	8.05	3.82
Gujarat	Dohad	34.07	7.27	5.82	21.75	11.95	3.23	23.17	3.33	2.39	30.19	12.56	6.62
Gujarat	Vadodara	27.77	8.72	4.15	20.54	9.98	3.29	24.63	3.76	2.39	22.50	13.46	5.48
Gujarat	Narmada	25.81	9.19	4.00	20.73	10.92	3.23	25.38	3.30	2.40	21.49	13.64	4.78
Gujarat	Bharuch	29.30	6.64	5.28	21.76	7.22	3.21	23.22	2.91	2.39	27.36	9.31	7.06
Gujarat	The Dangs	27.70	6.45	4.50	22.49	9.59	2.77	24.37	3.35	2.39	25.57	9.92	4.69
Gujarat	Navsari	26.99	6.77	4.13	21.15	7.55	2.93	23.32	2.94	2.39	24.21	9.59	4.99
Gujarat	Valsad	31.44	6.05	4.15	21.08	8.03	2.79	28.14	2.99	2.38	23.45	9.39	4.82
Gujarat	Surat	24.26	5.78	3.52	20.30	7.46	2.89	20.98	2.70	2.35	23.34	9.24	4.42
Gujarat	Tapi	26.58	5.94	3.68	20.22	8.33	2.90	23.11	3.20	2.41	22.66	8.65	4.02
Haryana	Panchkula	20.42	6.71	2.68	20.47	9.31	4.14	16.57	6.32	2.51	22.31	8.01	3.64
Haryana	Ambala	19.36	8.10	2.61	20.06	10.21	4.16	17.42	11.20	2.53	20.03	7.87	3.68
Haryana	Yamunanagar	20.51	6.42	2.62	20.26	9.21	4.21	17.69	6.18	2.51	21.24	8.05	3.61
Haryana	Kurukshetra	19.92	6.62	2.59	18.72	9.16	4.74	17.91	6.83	2.49	18.23	7.65	4.00
Haryana	Kaithal	24.70	7.13	2.62	21.71	14.25	4.51	17.58	8.87	2.52	26.52	10.60	3.79
Haryana	Karnal	21.19	6.38	3.01	20.42	8.85	4.10	19.08	6.08	2.48	20.16	7.74	4.07
Haryana	Panipat	21.15	7.28	3.05	19.44	10.42	4.76	16.80	9.36	2.54	21.22	8.04	4.12
Haryana	Sonipat	19.17	6.70	2.99	19.27	10.13	4.07	16.74	7.60	2.49	19.09	7.89	4.11
Haryana	Jind	20.27	6.34	2.61	20.90	11.53	4.51	16.74	6.84	2.52	21.82	9.06	3.83
Haryana	Fatehabad	22.64	6.47	2.61	21.24	8.69	4.04	17.15	7.18	2.52	24.51	7.10	3.42
Haryana	Sirsa	25.32	9.25	2.53	20.96	10.34	3.97	17.93	6.50	2.48	25.36	11.07	3.33
Haryana	Hisar	22.90	11.08	2.58	21.59	10.14	5.35	19.21	12.05	2.47	23.72	10.02	4.62

Haryana	Bhiwani	26.45	8.81	2.53	22.16	10.23	5.86	18.72	13.91	2.57	27.30	8.18	4.24
Haryana	Rohtak	26.61	6.60	2.54	19.95	13.04	4.62	21.23	11.01	2.53	22.52	8.41	3.63
Haryana	Jhajjar	24.48	7.20	2.60	19.93	9.20	4.25	17.99	6.65	2.49	24.13	8.07	3.46
Haryana	Mahendragarh	20.48	7.99	2.59	19.31	12.71	4.82	17.27	9.69	2.48	19.69	10.14	3.89
Haryana	Rewari	23.87	8.88	2.61	19.24	14.04	4.19	17.45	6.70	2.51	22.71	13.31	3.51
Haryana	Gurgaon	19.91	6.51	2.98	18.99	12.16	4.73	16.49	9.34	2.57	19.82	8.60	4.08
Haryana	Mewat	28.18	13.75	4.19	27.52	12.69	8.74	20.93	8.30	2.48	32.44	14.77	9.53
Haryana	Faridabad	22.95	7.18	2.96	19.69	11.30	4.15	18.90	8.12	2.49	21.38	9.13	4.31
Haryana	Palwal	21.72	7.64	2.89	20.19	14.82	3.95	17.93	10.37	2.52	21.20	11.00	3.69
Himachal Pradesh	Chamba	34.80	13.89	3.29	19.43	7.91	2.49	17.12	7.94	2.63	31.36	11.10	2.95
Himachal Pradesh	Kangra	29.35	8.53	2.87	17.84	7.25	2.29	17.16	6.21	2.65	24.49	7.96	2.32
Himachal Pradesh	Lahul And Spiti	27.84	7.70	2.97	17.51	8.49	2.22	.	.	.	22.40	8.34	2.41
Himachal Pradesh	Kullu	24.56	8.46	3.34	18.52	8.00	2.20	17.11	6.10	2.62	22.07	8.63	2.60
Himachal Pradesh	Mandi	24.69	7.43	2.90	19.15	8.97	2.57	17.94	5.81	2.68	21.97	8.50	2.63
Himachal Pradesh	Hamirpur	26.99	7.50	2.85	18.75	9.15	2.20	16.90	5.99	2.66	23.87	8.67	2.30
Himachal Pradesh	Una	26.75	7.42	3.26	17.33	8.98	2.27	17.19	6.20	2.66	22.39	8.58	2.63
Himachal Pradesh	Bilaspur	24.46	7.51	3.32	16.97	7.17	2.23	16.98	5.93	2.66	19.57	7.16	2.60
Himachal Pradesh	Solan	28.01	7.55	2.91	18.26	8.06	2.15	16.82	5.66	2.67	25.04	8.03	2.34
Himachal Pradesh	Sirmaur	30.15	8.13	2.86	19.01	8.88	2.21	18.71	5.91	2.66	25.68	9.01	2.27
Himachal Pradesh	Shimla	27.61	10.94	2.93	16.87	7.08	2.21	17.04	5.74	2.65	21.78	9.58	2.32
Himachal Pradesh	Kinnaur	27.42	9.70	2.93	18.36	7.48	2.22	.	.	.	24.10	9.09	2.39
Jammu And Kashmir	Kupwara	24.55	8.13	2.91	20.30	7.51	3.74	26.55	5.30	2.78	21.54	8.24	3.44
Jammu And Kashmir	Badgam	20.96	11.20	2.98	21.27	7.74	2.94	26.74	4.78	2.74	19.75	10.84	2.80
Jammu And Kashmir	Leh	20.29	9.15	3.37	21.35	7.14	3.04	23.25	4.44	2.78	21.46	9.78	3.04
Jammu And Kashmir	Kargil	27.01	9.31	3.68	19.83	7.86	2.94	26.45	5.14	2.75	23.44	9.57	3.62
Jammu And Kashmir	Punch	22.61	8.71	3.18	20.62	7.21	3.23	24.83	4.97	2.83	21.32	8.56	2.89
Jammu And Kashmir	Rajouri	22.49	9.40	3.23	22.21	6.54	2.84	25.37	5.15	2.79	22.89	8.52	3.04
Jammu And Kashmir	Kathua	24.18	7.97	2.98	22.25	6.99	3.39	25.12	5.15	2.75	24.48	7.82	3.24
Jammu And Kashmir	Baramula	22.94	15.28	2.86	20.17	6.40	3.00	24.87	4.96	2.76	21.14	12.34	2.92
Jammu And Kashmir	Bandipore	25.56	11.96	3.30	21.01	8.66	3.83	28.18	6.44	2.84	22.25	11.31	3.53
Jammu And Kashmir	Srinagar	24.11	10.67	3.40	20.96	6.50	3.06	25.96	6.15	2.80	21.13	8.99	3.19
Jammu And Kashmir	Ganderbal	29.06	8.69	3.26	19.84	8.44	3.31	29.08	4.86	2.78	22.28	9.80	3.52
Jammu And Kashmir	Pulwama	23.44	12.97	3.32	21.44	6.41	3.05	29.54	5.28	2.75	20.32	10.46	3.21
Jammu And Kashmir	Shupiyan	22.58	8.37	2.97	21.09	6.98	2.96	27.05	5.24	2.81	20.55	7.99	2.80
Jammu And Kashmir	Anantnag	24.57	11.07	2.95	21.43	8.99	3.38	30.09	12.47	2.73	20.37	8.99	3.28
Jammu And Kashmir	Kulgam	27.80	13.70	3.84	20.57	7.93	2.95	26.43	4.85	2.71	23.81	12.88	3.67
Jammu And Kashmir	Doda	23.91	9.69	2.92	21.27	8.50	3.35	26.76	5.23	2.79	22.09	10.23	3.07
Jammu And Kashmir	Ramban	24.75	9.45	3.25	22.27	7.52	2.88	26.98	5.43	2.80	23.15	8.99	3.07
Jammu And Kashmir	Kishtwar	28.00	10.47	2.90	19.47	7.76	3.84	27.14	5.31	2.78	22.69	10.20	3.44
Jammu And Kashmir	Udhampur	22.15	7.83	2.93	23.34	6.03	2.94	26.40	5.11	2.75	23.43	7.01	2.74
Jammu And Kashmir	Reasi	25.60	8.09	3.18	19.65	5.90	3.24	24.93	5.15	2.79	22.57	7.15	3.37
Jammu And Kashmir	Jammu	21.66	8.14	3.00	19.79	6.61	3.86	24.47	4.68	2.76	19.56	7.99	3.91
Jammu And Kashmir	Samba	21.83	8.90	3.32	19.02	6.40	3.01	24.61	5.13	2.78	18.56	8.04	3.24
Jharkhand	Garhwa	34.84	9.92	3.78	34.21	8.18	5.24	29.27	6.00	2.61	37.88	9.51	5.02
Jharkhand	Chatra	41.22	7.72	3.32	27.66	9.70	5.86	24.81	5.89	2.60	38.03	9.13	4.86
Jharkhand	Kodarma	30.87	9.07	3.80	24.87	8.32	4.89	23.30	6.71	2.62	28.45	8.88	4.71
Jharkhand	Giridih	35.83	6.38	3.75	26.54	12.19	7.65	24.95	9.88	2.62	33.23	8.22	6.56
Jharkhand	Deoghar	37.22	6.35	4.18	29.00	10.05	5.23	24.85	5.78	2.58	37.18	8.21	5.62
Jharkhand	Godda	39.12	8.56	3.48	25.85	9.53	9.43	25.49	5.93	2.62	33.92	9.60	7.86
Jharkhand	Sahibganj	33.53	10.86	4.37	29.68	11.29	5.28	27.90	5.70	2.59	32.95	12.78	5.61
Jharkhand	Pakur	34.00	10.99	4.29	24.19	10.12	6.12	25.86	5.69	2.59	28.58	12.02	6.29
Jharkhand	Dhanbad	36.47	8.49	3.63	26.95	7.73	5.67	29.47	6.02	2.56	31.10	8.44	6.01
Jharkhand	Bokaro	32.74	6.79	3.68	24.72	11.89	4.51	23.70	7.24	2.55	30.54	9.65	4.78
Jharkhand	Lohardaga	30.00	7.62	3.50	24.53	7.51	6.12	27.00	5.78	2.62	24.39	7.65	5.28
Jharkhand	Purbi Singhbhum	25.22	6.45	3.31	25.25	8.27	4.79	24.97	4.55	2.59	23.01	8.13	4.47

Jharkhand	Palamu	35.13	9.56	3.46	27.14	9.25	6.19	25.86	5.89	2.57	32.41	10.29	5.27
Jharkhand	Latehar	31.12	9.45	3.86	26.83	9.50	4.89	25.33	5.90	2.58	29.48	10.22	4.65
Jharkhand	Hazaribagh	30.39	9.23	4.33	30.15	8.44	4.83	29.25	7.16	2.59	29.99	8.78	5.23
Jharkhand	Ramgarh	34.99	8.45	4.11	25.62	7.64	5.56	29.35	4.86	2.69	27.87	9.20	5.42
Jharkhand	Dumka	36.40	10.70	3.48	31.52	9.60	4.93	26.79	8.04	2.60	39.10	10.45	4.24
Jharkhand	Jamtara	35.76	8.15	3.79	28.17	9.21	5.97	27.92	7.32	2.61	32.37	8.73	5.63
Jharkhand	Ranchi	33.72	7.30	3.82	26.55	11.50	5.19	24.22	6.47	2.67	33.19	10.34	4.37
Jharkhand	Khunti	26.07	8.90	3.98	25.79	9.56	4.89	27.11	5.85	2.58	23.14	9.92	4.83
Jharkhand	Gumla	31.45	7.07	4.43	27.38	8.53	5.48	25.29	5.96	2.59	29.92	7.81	6.05
Jharkhand	Simdega	34.41	8.86	5.17	29.69	9.60	6.11	26.35	7.81	2.59	34.67	9.24	7.59
Jharkhand	Pashchimi Singhbhum	34.01	12.84	4.98	28.44	13.77	8.32	26.08	5.76	2.58	33.05	16.36	9.58
Jharkhand	Saraikela Kharsawan	34.90	8.85	3.99	25.64	12.14	7.11	23.72	7.01	2.68	32.19	11.04	6.34
Karnataka	Belgaum	22.37	8.51	2.80	16.22	6.63	2.75	17.32	3.79	2.68	19.26	9.54	2.66
Karnataka	Bagalkot	21.21	10.11	2.47	18.52	6.14	3.22	19.39	3.39	2.68	20.68	10.81	2.76
Karnataka	Bijapur	22.78	9.28	2.47	17.68	6.62	2.80	16.55	3.86	2.72	23.23	10.10	2.40
Karnataka	Bidar	26.49	9.56	2.46	16.88	6.12	2.80	17.47	4.97	2.69	23.40	8.66	2.35
Karnataka	Raichur	27.61	10.28	2.43	18.04	5.88	2.72	20.66	3.62	2.74	23.68	9.94	2.30
Karnataka	Koppal	21.99	10.26	2.80	16.67	7.48	3.11	17.14	5.10	2.71	20.16	10.67	3.04
Karnataka	Gadag	22.61	8.52	2.45	16.49	6.80	2.81	17.33	4.52	2.70	20.24	9.39	2.42
Karnataka	Dharwad	23.56	8.47	2.46	18.55	8.50	2.81	20.02	3.61	2.70	21.77	12.25	2.40
Karnataka	Uttara Kannada	20.95	7.91	2.57	17.79	6.95	2.81	18.18	3.69	2.66	19.97	9.35	2.44
Karnataka	Haveri	21.57	7.77	2.90	15.98	6.97	2.84	17.17	4.02	2.70	18.83	8.77	2.70
Karnataka	Bellary	23.58	7.27	2.83	19.37	5.88	3.59	19.41	3.47	2.91	23.56	8.30	2.38
Karnataka	Chitradurga	21.64	17.60	2.55	17.76	7.00	2.81	16.85	3.89	2.71	21.16	16.83	2.43
Karnataka	Davanagere	25.96	10.67	2.51	16.31	6.84	3.20	19.43	3.63	2.68	20.63	12.03	2.71
Karnataka	Shimoga	25.05	7.80	2.50	16.74	7.93	2.81	18.80	4.77	2.69	21.43	9.69	2.49
Karnataka	Udupi	23.14	7.88	2.52	16.78	6.36	2.83	16.74	4.00	2.75	21.74	8.49	2.43
Karnataka	Chikmagalur	20.98	9.28	2.54	16.17	6.08	2.88	17.23	3.80	2.71	18.44	9.42	2.44
Karnataka	Tumkur	26.97	10.01	2.51	19.66	8.05	2.85	18.11	3.97	2.76	28.25	11.86	2.41
Karnataka	Bangalore	20.85	8.68	2.53	16.71	7.08	3.24	15.86	5.27	2.73	21.74	9.69	2.49
Karnataka	Mandya	21.86	9.14	2.94	17.97	6.24	2.94	17.24	3.96	2.73	21.78	9.24	2.85
Karnataka	Hassan	21.57	7.90	2.57	17.11	6.15	3.19	17.81	3.99	2.70	20.26	8.70	2.83
Karnataka	Dakshina Kannada	21.67	7.90	2.51	16.63	6.28	3.22	16.49	3.75	2.69	20.57	8.79	2.70
Karnataka	Kodagu	23.40	8.06	2.53	15.96	6.11	2.80	17.13	4.02	2.73	20.02	8.27	2.35
Karnataka	Mysore	21.87	7.81	2.47	16.59	7.74	3.22	16.25	3.72	2.76	21.43	10.59	2.41
Karnataka	Chamarajanagar	22.40	10.08	2.88	17.65	7.77	3.18	16.84	3.81	2.72	22.18	12.10	3.19
Karnataka	Gulbarga	23.31	8.12	2.44	15.65	6.60	2.79	17.67	5.31	2.69	19.41	8.23	2.41
Karnataka	Yadgir	20.46	9.02	2.46	17.00	6.63	2.77	16.60	3.78	2.67	19.85	9.74	2.32
Karnataka	Kolar	24.60	8.84	2.57	17.54	7.72	3.22	19.08	5.95	2.65	22.02	9.46	2.78
Karnataka	Chikkaballapura	22.15	7.87	2.88	16.22	6.99	3.25	17.78	3.87	2.76	18.95	9.33	2.75
Karnataka	Bangalore Rural	24.09	9.88	2.47	16.37	5.95	2.76	18.85	3.81	2.70	19.99	9.73	2.33
Karnataka	Ramanagara	21.43	7.93	2.86	16.70	6.25	2.84	17.51	3.83	2.72	19.60	8.81	2.41
Kerala	Kasaragod	7.21	5.53	2.59	8.36	5.02	2.08	7.46	6.34	2.62	7.62	4.67	1.87
Kerala	Kannur	7.46	4.90	2.60	8.47	4.56	2.33	7.48	3.92	2.68	8.00	4.76	1.84
Kerala	Wayanad	7.09	5.77	2.57	8.92	5.59	1.99	7.65	4.53	2.64	7.94	5.71	1.75
Kerala	Kozhikode	7.46	5.09	2.59	8.82	4.45	2.07	8.25	3.93	2.57	7.68	4.71	1.84
Kerala	Malappuram	7.44	4.90	2.57	8.35	4.42	2.05	7.67	3.70	2.60	7.54	4.60	1.85
Kerala	Palakkad	7.13	4.97	2.58	8.44	4.41	2.00	7.59	4.18	2.61	7.33	4.47	1.84
Kerala	Thrissur	7.14	5.11	2.64	8.58	4.49	2.11	7.47	4.04	2.63	7.59	4.68	1.86
Kerala	Ernakulam	7.21	5.20	3.03	8.45	4.49	2.07	7.51	4.01	2.71	7.68	4.69	1.90
Kerala	Idukki	7.17	5.21	2.61	8.42	4.47	2.08	7.73	4.43	2.64	7.44	4.56	1.86
Kerala	Kottayam	7.19	5.21	2.66	8.67	4.53	2.07	7.66	4.15	2.61	7.76	4.53	1.84
Kerala	Alappuzha	7.21	5.19	2.57	9.12	4.50	2.08	7.90	4.10	2.61	7.93	4.71	1.88
Kerala	Pathanamthitta	7.24	5.16	2.66	8.42	4.62	2.08	7.76	4.56	2.62	7.45	4.61	1.86
Kerala	Kollam	7.17	5.07	2.60	8.65	4.53	2.07	7.45	3.86	2.62	7.96	4.70	1.87

Kerala	Thiruvananthapuram	7.48	5.74	2.65	8.49	4.43	2.06	7.55	5.28	2.62	7.97	4.64	1.84
Lakshadweep	Lakshadweep	17.45	8.11	2.96	24.15	6.18	2.76	17.09	3.55	2.60	28.40	11.81	3.33
Madhya Pradesh	Sheopur	35.99	15.80	5.88	29.10	12.08	7.01	27.01	13.69	3.41	34.66	14.04	7.82
Madhya Pradesh	Morena	36.62	15.22	5.35	30.08	14.63	6.92	27.56	14.71	3.53	35.87	15.29	6.28
Madhya Pradesh	Bhind	37.81	10.40	4.74	32.22	12.60	6.40	32.33	9.64	3.37	36.12	11.58	6.25
Madhya Pradesh	Gwalior	45.71	14.62	5.80	32.14	18.76	6.30	37.58	11.84	3.39	38.93	19.50	7.40
Madhya Pradesh	Datia	45.55	10.27	4.84	27.82	16.79	9.06	28.58	11.57	3.41	39.02	13.86	8.39
Madhya Pradesh	Shivpuri	42.64	11.29	4.08	28.96	15.77	7.04	28.20	13.75	3.39	38.55	13.36	5.94
Madhya Pradesh	Tikamgarh	32.93	12.67	4.71	33.50	12.43	6.46	32.53	14.02	3.46	32.77	12.12	6.12
Madhya Pradesh	Chhatarpur	35.26	15.86	5.86	30.90	16.31	7.05	29.85	18.69	3.43	34.65	15.77	7.52
Madhya Pradesh	Panna	43.59	12.69	4.79	35.78	12.99	6.56	28.27	9.27	3.41	48.31	13.70	6.05
Madhya Pradesh	Sagar	47.67	10.91	4.15	29.36	9.23	7.24	31.53	7.87	3.46	40.67	10.66	5.58
Madhya Pradesh	Damoh	35.05	14.27	4.25	32.05	13.94	6.59	32.02	17.50	3.42	33.31	13.52	5.53
Madhya Pradesh	Satna	36.86	10.72	4.83	30.54	12.76	5.93	30.61	17.03	3.45	34.06	10.25	5.70
Madhya Pradesh	Rewa	48.43	14.68	5.80	32.53	16.49	7.15	29.37	17.33	3.63	46.92	15.38	6.55
Madhya Pradesh	Umaria	41.47	13.15	5.47	29.09	12.55	7.99	28.78	14.57	3.39	37.13	12.03	8.46
Madhya Pradesh	Neemuch	33.52	15.62	4.25	27.96	10.62	6.53	25.72	15.63	3.47	32.42	12.21	5.17
Madhya Pradesh	Mandsaur	38.78	13.63	4.30	29.70	13.10	5.76	28.66	8.65	3.44	36.36	15.67	4.87
Madhya Pradesh	Ratlam	35.49	10.39	4.72	29.56	10.31	5.72	28.21	9.57	3.38	34.34	10.27	5.74
Madhya Pradesh	Ujjain	40.02	11.47	4.63	26.01	8.78	5.95	33.88	7.34	3.56	28.96	11.44	4.92
Madhya Pradesh	Shajapur	36.89	13.80	4.73	29.41	9.46	7.25	29.10	10.99	3.47	33.61	11.61	6.39
Madhya Pradesh	Dewas	41.15	11.14	4.19	35.43	12.25	7.10	38.20	16.99	3.42	38.19	10.02	6.06
Madhya Pradesh	Dhar	46.43	13.37	5.12	32.07	8.94	7.73	30.01	8.45	3.46	44.30	11.48	7.19
Madhya Pradesh	Indore	32.24	8.71	3.85	24.56	11.71	5.18	24.40	7.54	3.24	31.89	12.30	5.80
Madhya Pradesh	Khargone (West Nimar)	40.02	10.27	5.31	30.36	10.17	6.42	34.05	11.37	3.35	33.90	9.57	6.68
Madhya Pradesh	Barwani	37.48	10.69	3.89	32.98	9.28	7.22	30.14	9.01	3.39	37.02	9.75	5.64
Madhya Pradesh	Rajgarh	43.15	14.00	4.15	27.89	13.47	7.12	29.84	9.12	3.41	36.02	15.58	6.08
Madhya Pradesh	Vidisha	38.55	22.60	4.13	29.63	15.89	7.14	27.91	13.54	3.39	36.56	21.85	5.93
Madhya Pradesh	Bhopal	36.75	9.58	4.83	31.57	11.86	7.38	31.19	6.20	3.65	35.76	13.92	5.94
Madhya Pradesh	Sehore	35.31	13.90	4.75	28.59	12.38	7.12	27.02	20.75	3.36	33.76	11.28	6.68
Madhya Pradesh	Raisen	37.47	11.07	4.17	28.95	10.89	7.77	29.83	7.95	3.47	33.55	11.80	5.98
Madhya Pradesh	Betul	35.42	10.91	4.81	27.90	14.40	7.10	27.18	8.94	3.38	32.70	13.53	7.08
Madhya Pradesh	Harda	35.22	11.19	5.92	27.55	12.48	8.11	28.90	8.49	3.57	30.66	12.93	7.29
Madhya Pradesh	Hoshangabad	36.60	9.71	5.42	28.81	9.29	6.33	28.68	8.55	3.42	33.94	9.77	7.00
Madhya Pradesh	Katni	42.66	20.62	4.35	31.72	14.18	6.47	27.81	9.47	3.41	42.89	20.39	5.63
Madhya Pradesh	Jabalpur	41.44	8.99	4.49	32.39	10.21	7.32	30.01	8.77	3.31	43.08	9.46	7.33
Madhya Pradesh	Narsimhapur	43.19	10.97	4.33	28.50	9.76	6.61	30.19	9.50	3.38	36.95	10.32	5.66
Madhya Pradesh	Dindori	41.30	17.28	4.77	29.56	9.33	5.82	28.56	13.19	3.42	37.47	12.93	5.48
Madhya Pradesh	Mandla	41.77	12.06	6.31	30.21	9.59	5.92	29.77	9.55	3.39	38.31	11.01	7.07
Madhya Pradesh	Chhindwara	45.26	13.18	4.81	31.13	17.13	5.82	30.55	13.65	3.41	42.04	16.09	5.59
Madhya Pradesh	Seoni	32.61	12.06	4.32	33.30	14.34	7.34	31.04	9.58	3.43	33.22	14.55	6.32
Madhya Pradesh	Balaghat	39.29	11.78	4.31	33.50	10.25	6.46	30.52	15.14	3.43	39.40	9.64	5.54
Madhya Pradesh	Guna	41.37	11.80	5.71	29.50	13.61	6.09	32.29	7.82	3.36	35.34	14.62	7.09
Madhya Pradesh	Ashoknagar	35.36	10.08	4.68	27.87	13.41	7.08	31.10	10.45	3.45	29.32	12.01	5.95
Madhya Pradesh	Shahdol	34.01	14.99	7.10	32.05	14.54	6.69	30.92	9.38	3.40	33.29	17.54	8.84
Madhya Pradesh	Anuppur	35.37	11.37	5.34	30.46	11.65	5.82	31.44	8.54	3.50	32.35	12.66	5.68
Madhya Pradesh	Sidhi	38.91	14.16	6.47	32.76	8.96	11.51	29.13	9.25	3.50	39.13	11.65	11.60
Madhya Pradesh	Singrauli	34.16	18.72	6.40	28.22	12.54	7.54	27.37	10.27	3.58	31.84	17.76	7.58
Madhya Pradesh	Jhabua	38.05	13.45	6.33	29.18	9.36	7.19	28.20	9.50	3.44	34.86	11.66	7.76
Madhya Pradesh	Alirajpur	40.96	13.90	4.68	28.98	10.18	8.12	27.97	9.09	3.41	36.35	12.36	7.22
Madhya Pradesh	Khandwa (East Nimar)	37.52	10.00	4.16	27.08	12.21	5.67	27.45	10.74	3.37	32.13	10.75	4.70
Madhya Pradesh	Burhanpur	44.39	13.74	4.98	26.60	11.65	6.14	29.84	13.84	3.42	37.75	11.92	6.02
Maharashtra	Nandurbar	23.95	6.75	2.46	15.10	4.82	1.89	23.68	5.05	2.45	17.30	5.68	2.16
Maharashtra	Dhule	18.79	6.72	2.78	15.13	5.51	1.67	20.16	4.92	2.51	14.89	6.42	1.93
Maharashtra	Jalgaon	23.88	6.03	2.45	15.56	4.98	1.96	21.08	4.57	2.50	18.93	5.74	2.01

Maharashtra	Buldana	20.78	6.76	2.38	14.10	4.91	1.96	18.60	6.67	2.42	16.47	5.44	2.21
Maharashtra	Akola	23.92	6.58	2.46	14.83	5.42	1.73	23.81	5.46	2.45	15.86	6.10	2.00
Maharashtra	Washim	21.46	6.62	2.43	14.08	6.04	1.69	20.27	6.37	2.46	15.63	6.37	1.90
Maharashtra	Amravati	21.59	6.15	2.40	14.00	5.59	1.73	19.51	6.01	2.44	16.26	5.70	1.98
Maharashtra	Wardha	22.89	7.03	2.50	14.39	5.01	1.72	21.10	4.57	2.43	16.22	6.50	2.01
Maharashtra	Nagpur	19.64	6.64	2.76	14.01	4.91	1.73	18.39	4.34	2.43	15.68	6.32	2.31
Maharashtra	Bhandara	25.69	7.05	2.48	15.08	4.97	1.71	18.84	5.09	2.44	21.85	6.00	1.94
Maharashtra	Gondiya	21.06	5.99	2.42	14.12	6.31	1.71	19.78	5.13	2.45	15.73	6.42	1.94
Maharashtra	Gadchiroli	24.26	6.22	2.47	14.10	4.98	1.72	19.07	5.28	2.45	18.89	5.41	1.96
Maharashtra	Chandrapur	22.41	6.11	2.82	13.74	5.57	1.70	18.76	4.76	2.44	17.05	6.26	2.24
Maharashtra	Yavatmal	21.50	6.76	2.74	14.68	4.88	1.93	19.41	4.92	2.45	17.35	5.94	2.52
Maharashtra	Nanded	22.00	7.99	2.32	16.61	4.67	1.69	18.75	7.85	2.43	20.79	5.54	1.87
Maharashtra	Hingoli	25.00	6.61	2.41	15.78	5.41	1.97	20.48	4.91	2.44	21.00	6.37	2.19
Maharashtra	Parbhani	24.81	7.34	2.72	15.13	4.74	1.70	25.02	4.12	2.54	15.79	6.76	1.97
Maharashtra	Jalna	22.61	6.41	2.43	14.78	5.38	1.69	18.30	4.83	2.46	19.21	6.23	1.90
Maharashtra	Aurangabad	18.83	8.41	2.70	14.20	6.75	1.69	17.69	8.53	2.49	15.84	7.32	1.95
Maharashtra	Nashik	21.65	8.18	3.10	13.48	4.78	1.92	18.52	5.45	2.46	16.18	6.53	2.56
Maharashtra	Thane	21.36	6.03	2.45	14.23	5.74	1.73	18.87	4.45	2.42	16.66	6.61	2.04
Maharashtra	Mumbai Suburban	21.74	7.07	2.51	13.98	6.50	1.75	18.30	8.39	2.45	.	.	.
Maharashtra	Mumbai	21.21	6.28	2.51	14.91	5.85	1.71	19.24	5.51	2.41	.	.	.
Maharashtra	Raigarh	22.20	6.85	2.49	15.84	5.63	1.70	19.47	6.02	2.42	19.54	6.34	2.00
Maharashtra	Pune	22.21	6.16	2.78	14.19	5.00	1.68	18.99	4.75	2.46	17.43	5.65	2.30
Maharashtra	Ahmadnagar	19.82	6.08	2.46	14.15	5.67	1.72	18.41	5.00	2.44	16.05	5.97	1.98
Maharashtra	Bid	23.24	6.65	2.47	14.34	5.35	1.72	19.07	6.09	2.45	18.46	5.87	1.95
Maharashtra	Latur	20.69	6.44	2.39	14.37	4.81	1.67	19.24	5.85	2.40	16.21	5.31	1.90
Maharashtra	Osmanabad	22.65	5.88	2.79	14.15	4.88	1.72	18.84	5.09	2.43	18.09	5.41	2.23
Maharashtra	Solapur	20.22	7.32	2.37	14.02	4.83	1.69	18.50	4.25	2.42	15.84	6.81	1.98
Maharashtra	Satara	21.20	6.99	2.48	14.24	5.56	1.74	18.76	6.76	2.45	17.12	6.06	1.96
Maharashtra	Ratnagiri	22.75	6.21	2.54	13.78	5.03	1.71	19.23	5.25	2.45	17.44	5.53	1.97
Maharashtra	Sindhudurg	22.02	6.21	2.51	14.71	5.92	1.74	19.40	5.19	2.49	17.72	6.24	1.96
Maharashtra	Kolhapur	19.45	8.91	2.44	13.96	5.14	1.72	17.74	6.03	2.45	15.90	7.01	2.02
Maharashtra	Sangli	20.35	6.96	2.50	14.63	5.53	1.71	19.39	5.07	2.41	16.27	6.66	1.96
Manipur	Senapati (Excluding 3 Sub-Divisions)	19.00	6.24	2.63	15.41	6.58	2.36	15.54	4.45	2.75	18.14	6.98	2.28
Manipur	Tamenglong	20.36	5.63	3.00	14.24	5.72	2.02	14.95	5.56	2.76	18.19	5.54	2.29
Manipur	Churachandpur	21.23	6.53	3.12	14.48	6.80	2.15	15.44	4.46	2.74	18.54	7.43	2.38
Manipur	Bishnupur	19.01	4.96	3.99	13.72	6.55	2.53	14.85	4.61	2.96	16.71	6.16	2.65
Manipur	Thoubal	17.48	5.52	2.47	16.40	5.21	2.23	15.65	3.85	2.74	18.54	6.04	2.07
Manipur	Imphal West	17.51	5.94	2.55	13.04	4.83	2.00	13.43	3.27	2.66	15.99	6.24	2.10
Manipur	Imphal East	18.89	6.36	2.86	13.48	6.16	2.00	15.41	3.89	2.68	16.10	7.35	2.37
Manipur	Ukhrul	18.76	5.83	3.18	15.10	6.01	2.13	15.01	4.33	2.76	18.13	6.30	2.35
Manipur	Chandel	18.70	5.80	2.70	13.33	8.24	2.37	14.64	5.59	2.78	16.05	7.39	2.32
Meghalaya	West Garo Hills	24.23	9.41	3.60	15.21	8.89	4.18	10.62	6.46	2.67	23.19	9.22	3.83
Meghalaya	East Garo Hills	16.51	11.54	5.67	12.99	9.98	3.54	10.42	8.17	2.68	13.70	10.96	5.56
Meghalaya	South Garo Hills	17.05	15.92	4.73	14.46	10.88	5.98	10.77	7.03	2.63	15.54	15.04	7.45
Meghalaya	West Khasi Hills	22.33	10.76	3.82	14.88	9.58	3.51	10.79	5.92	2.62	20.15	10.87	3.86
Meghalaya	Ribhoi	22.06	16.83	3.97	15.38	11.38	4.33	11.46	6.42	2.60	19.70	16.37	4.72
Meghalaya	East Khasi Hills	19.23	11.00	3.67	13.39	10.95	4.12	9.71	9.55	2.61	17.53	11.02	4.53
Meghalaya	Jaintia Hills	18.63	14.30	4.79	18.04	9.71	4.23	10.81	8.50	2.66	21.16	12.32	5.35
Mizoram	Mamit	11.04	16.57	4.24	11.80	25.50	3.57	12.41	11.73	2.46	10.50	24.95	5.85
Mizoram	Kolasib	10.41	34.93	3.36	12.58	26.83	3.34	12.69	29.35	2.43	10.29	32.93	5.00
Mizoram	Aizawl	11.59	20.32	3.24	12.45	23.93	3.27	12.49	13.67	2.50	11.63	30.89	4.18
Mizoram	Champhai	13.12	20.23	2.97	12.64	24.44	2.95	12.55	20.50	2.42	13.16	23.62	3.86
Mizoram	Serchhip	12.47	21.89	4.29	11.99	22.21	2.67	12.60	16.69	2.50	11.93	25.88	4.55
Mizoram	Lunglei	11.82	27.05	3.52	11.19	34.54	3.59	11.25	28.36	2.45	11.38	33.83	5.36
Mizoram	Lawngtlai	13.07	27.99	3.73	11.91	23.62	2.89	12.37	11.67	2.49	12.18	32.74	4.17

Mizoram	Saiha	13.00	49.04	3.70	10.95	37.18	2.67	12.46	29.64	2.48	10.79	54.50	3.80
Nagaland	Mon	15.66	10.71	3.78	13.24	9.93	4.19	11.82	7.63	2.88	13.66	11.13	4.35
Nagaland	Mokokchung	21.83	10.31	3.75	15.31	10.87	4.17	11.72	7.37	2.80	22.14	11.89	4.38
Nagaland	Zunheboto	19.99	8.52	3.64	14.68	11.63	3.97	11.82	10.18	2.79	19.06	9.70	4.00
Nagaland	Wokha	21.79	8.67	4.88	14.99	12.99	3.93	11.79	7.59	2.84	21.37	11.91	5.31
Nagaland	Dimapur	21.34	11.47	4.19	16.08	12.61	3.55	13.26	8.81	2.84	21.61	13.75	4.10
Nagaland	Phek	18.47	11.07	4.58	14.65	15.52	6.98	12.68	13.20	3.00	16.69	13.49	6.60
Nagaland	Tuensang	17.89	9.77	3.90	15.59	16.00	4.26	13.54	7.68	2.83	17.01	15.09	4.68
Nagaland	Longleng	19.51	8.24	3.54	14.25	12.36	4.49	11.94	10.34	2.87	17.94	9.89	4.45
Nagaland	Kiphire	23.08	14.62	4.54	17.10	13.75	3.86	11.65	13.62	2.84	26.89	14.84	4.93
Nagaland	Kohima	20.63	8.73	3.91	14.37	15.25	4.19	11.34	10.34	2.78	20.69	12.36	4.88
Nagaland	Peren	22.78	14.18	3.99	13.97	11.57	5.46	12.29	7.59	2.90	19.88	15.11	5.24
Delhi	North West	15.87	9.28	3.76	18.95	12.00	3.18	16.98	10.57	3.00	19.32	9.42	3.31
Delhi	North	18.16	9.05	3.61	18.92	10.19	3.13	19.21	7.74	3.01	19.00	9.32	3.42
Delhi	North East	16.76	9.72	3.61	18.03	15.81	3.55	16.39	15.50	3.06	.	.	.
Delhi	East	16.79	9.32	3.64	17.18	17.09	3.16	15.23	16.46	3.01	.	.	.
Delhi	New Delhi	17.22	9.63	3.68	17.39	10.92	3.19	15.79	9.06	3.02	.	.	.
Delhi	Central	16.50	9.63	3.79	19.05	13.68	3.12	17.00	13.05	2.99	.	.	.
Delhi	West	16.57	9.27	3.70	18.10	11.05	3.19	16.20	8.48	2.97	.	.	.
Delhi	South West	16.27	7.96	4.13	17.10	10.50	3.62	15.00	6.45	3.14	19.00	9.40	3.35
Delhi	South	19.32	8.17	6.14	17.64	13.91	3.57	18.20	10.15	3.41	.	.	.
Odisha	Bargarh	26.41	11.55	3.54	30.66	10.04	3.03	19.36	4.64	2.79	29.97	12.60	3.20
Odisha	Jharsuguda	28.71	11.42	3.42	30.53	7.93	3.67	20.94	4.46	2.93	32.73	12.09	3.28
Odisha	Sambalpur	29.70	8.50	3.65	31.45	7.98	3.43	21.05	4.29	2.78	31.91	8.99	3.73
Odisha	Debagarh	29.66	11.71	3.61	29.58	10.67	2.95	19.60	4.80	2.81	31.33	12.81	3.23
Odisha	Sundargarh	27.24	8.33	4.29	27.10	8.70	3.59	20.63	4.37	2.74	26.63	9.74	5.16
Odisha	Kendujhar	30.70	8.85	4.02	34.15	8.67	2.94	20.10	4.65	2.80	36.63	9.46	3.57
Odisha	Mayurbhanj	26.51	13.99	5.15	26.66	9.88	2.99	19.57	4.82	2.85	24.96	14.07	4.60
Odisha	Baleshwar	25.57	8.34	4.05	30.85	7.97	3.01	19.30	4.78	2.78	29.42	8.32	3.69
Odisha	Bhadrak	30.59	8.28	4.11	28.39	9.94	2.95	20.86	4.59	2.80	29.39	9.95	3.66
Odisha	Kendrapara	26.24	12.67	3.55	27.61	9.81	2.91	20.55	6.34	2.83	25.13	11.89	3.15
Odisha	Jagatsinghapur	32.19	9.50	3.64	28.62	10.49	3.03	20.61	4.80	2.79	30.78	11.36	3.31
Odisha	Cuttack	27.02	11.97	3.60	28.13	8.36	2.93	18.86	4.38	2.83	28.82	11.53	3.37
Odisha	Jajapur	27.99	8.02	3.53	30.36	11.18	3.03	19.19	4.55	2.80	31.51	10.70	3.24
Odisha	Dhenkanal	33.39	8.36	3.62	29.10	9.02	2.97	21.19	4.53	2.81	32.65	9.21	3.21
Odisha	Anugul	31.44	8.00	4.06	35.79	8.83	3.43	19.84	4.49	2.88	40.69	9.04	3.63
Odisha	Nayagarh	30.73	9.20	3.56	31.76	8.18	3.44	19.69	4.79	2.81	35.23	9.22	3.65
Odisha	Khordha	22.76	9.15	4.82	27.89	8.31	2.83	18.03	3.71	2.87	25.82	10.55	4.06
Odisha	Puri	33.50	8.52	3.60	29.15	10.10	2.99	20.54	5.93	2.81	33.46	9.36	3.23
Odisha	Ganjam	27.61	14.21	3.62	28.35	8.21	2.94	18.61	4.34	2.78	29.41	13.30	3.36
Odisha	Gajapati	31.99	8.92	3.98	30.85	10.00	3.34	19.45	4.58	2.77	35.04	10.46	3.98
Odisha	Kandhamal	36.63	12.31	4.42	31.03	9.33	3.01	19.98	4.61	2.79	37.97	12.09	3.92
Odisha	Baudh	32.78	12.30	4.44	32.10	9.69	4.25	19.89	4.83	2.82	36.26	12.67	5.64
Odisha	Subarnapur	31.10	10.43	3.56	26.76	11.40	2.97	19.36	4.71	2.80	29.34	12.65	3.23
Odisha	Balangir	33.16	9.36	3.55	29.17	8.73	3.35	20.05	4.46	2.82	33.09	9.85	3.62
Odisha	Nuapada	28.71	8.01	3.54	32.94	8.57	2.90	20.24	6.24	2.80	33.17	7.79	3.09
Odisha	Kalahandi	30.96	11.38	3.55	28.79	11.28	3.39	22.33	8.29	2.81	28.51	11.66	3.69
Odisha	Rayagada	33.92	12.58	3.54	34.46	11.91	4.82	20.02	4.62	2.87	40.45	14.92	4.51
Odisha	Nabarangapur	35.19	9.67	4.37	32.44	10.94	3.69	19.20	6.02	2.85	39.10	10.89	4.76
Odisha	Koraput	28.86	9.93	3.95	29.74	7.83	2.87	19.67	4.52	2.86	30.77	9.69	3.15
Odisha	Malkangiri	33.28	16.48	6.80	28.91	18.87	2.85	20.23	4.61	2.81	32.44	22.66	5.78
Puducherry	Yanam	16.69	7.55	4.07	9.60	8.53	2.95	9.82	6.95	2.96	.	.	.
Puducherry	Puducherry	16.01	8.47	3.16	9.70	8.74	2.63	9.32	5.53	2.74	18.07	11.83	2.73
Puducherry	Mahe	15.53	6.81	3.21	9.57	6.71	2.60	9.17	3.81	2.73	.	.	.
Puducherry	Karaikal	15.82	6.63	3.18	10.26	6.90	2.55	10.14	4.27	2.79	17.92	9.03	2.78

Punjab	Gurdaspur	22.38	6.09	3.00	21.94	9.78	1.56	18.37	4.99	2.36	24.79	9.09	2.56
Punjab	Kapurthala	23.83	6.01	3.99	20.82	9.11	1.55	18.07	4.68	2.32	24.70	8.78	3.48
Punjab	Jalandhar	23.19	6.82	3.01	21.21	10.02	1.54	17.99	7.25	2.34	25.03	9.03	2.65
Punjab	Hoshiarpur	23.03	6.07	3.03	20.62	10.03	1.54	17.54	6.64	2.37	23.91	8.55	2.56
Punjab	Sangrur	24.52	7.03	3.52	21.02	7.69	1.50	21.24	5.16	2.33	22.06	8.24	3.03
Punjab	Fatehgarh Sahib	21.12	5.96	3.05	22.78	11.11	1.75	18.38	7.58	2.38	24.63	8.78	2.63
Punjab	Ludhiana	22.80	6.79	3.40	22.49	8.75	1.55	19.16	4.64	2.32	25.20	9.63	2.97
Punjab	Moga	22.36	7.60	3.87	24.07	8.64	1.54	20.17	4.94	2.33	25.21	9.59	3.23
Punjab	Firozpur	24.66	6.78	3.42	21.43	7.67	1.53	18.35	4.95	2.33	26.03	8.10	2.88
Punjab	Muktsar	23.27	10.60	3.52	20.51	11.20	1.53	18.13	10.39	2.36	23.57	11.69	2.94
Punjab	Faridkot	23.28	6.76	2.99	20.90	7.63	1.53	17.01	4.45	2.34	25.82	8.60	2.59
Punjab	Bathinda	21.92	6.02	3.06	20.84	9.84	1.55	18.89	4.51	2.32	22.60	9.73	2.65
Punjab	Mansa	23.94	5.87	3.05	20.75	9.47	1.52	18.59	4.98	2.38	24.27	8.65	2.49
Punjab	Patiala	21.13	7.51	3.01	20.75	9.52	1.53	17.41	4.48	2.37	23.18	11.17	2.60
Punjab	Amritsar	20.07	6.58	2.95	22.12	8.68	1.54	17.99	5.69	2.34	22.82	8.44	2.61
Punjab	Tarn Taran	22.64	6.72	3.39	21.96	8.44	1.54	19.43	6.82	2.35	23.83	7.87	2.84
Punjab	Rupnagar	21.77	6.84	3.01	22.70	7.89	1.50	18.16	4.88	2.32	24.63	8.39	2.53
Punjab	Sahibzada Ajit Singh Nagar	23.46	6.74	3.04	21.70	7.67	1.54	18.01	4.75	2.32	25.82	8.49	2.64
Punjab	Shahid Bhagat Singh Nagar	21.21	6.04	2.97	19.89	7.98	1.56	17.04	4.52	2.33	21.90	7.98	2.65
Punjab	Barnala	26.10	6.03	3.09	22.42	8.88	1.54	18.98	6.04	2.31	28.78	7.92	2.63
Rajasthan	Ganganagar	29.63	8.75	3.91	29.43	7.97	4.65	21.08	5.18	2.88	32.06	9.02	4.38
Rajasthan	Hanumangarh	32.05	8.23	3.42	26.10	8.02	4.69	19.29	5.40	2.92	32.64	8.58	4.00
Rajasthan	Bikaner	27.75	7.73	4.25	27.86	11.42	4.48	19.81	7.56	3.15	31.52	10.03	3.40
Rajasthan	Churu	27.84	9.57	3.40	26.41	9.65	4.63	21.72	6.47	2.82	26.70	10.39	4.34
Rajasthan	Jhunjhunun	31.04	10.95	3.51	25.98	12.20	4.07	20.10	13.70	2.79	29.91	10.60	3.95
Rajasthan	Alwar	29.89	10.15	3.73	26.52	8.74	5.80	21.12	5.65	2.81	28.53	10.29	5.71
Rajasthan	Bharatpur	30.95	9.78	4.63	26.73	8.87	4.95	20.18	6.76	2.84	30.93	9.77	5.97
Rajasthan	Dhaulpur	28.36	12.73	3.64	25.78	12.03	4.48	18.55	6.86	2.80	28.81	14.23	4.42
Rajasthan	Karauli	33.09	10.29	3.71	29.07	14.65	5.04	19.15	5.66	2.82	36.56	14.78	4.93
Rajasthan	Sawai Madhopur	31.69	13.50	3.73	30.15	10.36	4.52	22.87	6.53	2.83	33.00	13.58	4.66
Rajasthan	Dausa	24.42	17.23	3.43	27.63	12.74	5.73	20.30	5.65	2.83	25.61	18.94	5.16
Rajasthan	Jaipur	28.05	10.54	4.36	26.80	11.41	4.21	20.92	10.41	2.86	28.72	10.69	4.87
Rajasthan	Sikar	30.32	7.89	3.80	28.62	8.59	4.01	18.10	5.11	2.88	35.33	8.72	3.83
Rajasthan	Nagaur	28.42	8.74	3.37	26.04	9.98	4.74	19.92	5.43	2.81	28.86	10.43	4.30
Rajasthan	Jodhpur	30.95	8.57	3.12	27.36	12.22	4.13	19.30	3.89	2.75	34.25	14.03	3.88
Rajasthan	Jaisalmer	31.75	10.54	3.63	29.49	13.15	5.06	21.14	7.57	2.80	32.84	12.89	4.75
Rajasthan	Barmer	28.60	10.69	4.17	31.76	10.72	3.99	19.58	5.83	2.85	34.05	11.88	4.26
Rajasthan	Jalor	41.10	9.86	3.74	29.06	8.23	3.94	21.27	7.58	2.84	40.60	9.07	3.84
Rajasthan	Sirohi	32.54	13.74	4.78	31.90	8.56	4.51	22.35	5.35	2.82	36.64	12.83	5.66
Rajasthan	Pali	33.82	13.09	3.45	26.00	8.00	4.63	21.83	5.44	2.83	30.85	11.98	4.15
Rajasthan	Ajmer	26.30	6.96	3.16	25.97	6.77	4.72	17.47	4.93	2.85	30.07	7.23	3.94
Rajasthan	Tonk	27.59	8.82	3.37	29.53	8.80	5.29	19.23	6.56	2.82	32.89	8.98	4.92
Rajasthan	Bundi	25.98	11.18	3.92	29.10	9.97	4.08	22.51	5.42	2.84	27.47	12.37	4.36
Rajasthan	Bhilwara	34.35	10.20	4.01	29.34	8.19	4.10	18.72	5.41	2.90	39.94	9.97	3.86
Rajasthan	Rajsamand	33.62	11.28	4.85	26.38	11.91	4.18	21.07	7.59	2.81	32.11	12.92	5.20
Rajasthan	Dungarpur	29.42	8.13	3.26	26.65	9.46	4.55	19.90	5.99	2.85	29.52	9.11	3.98
Rajasthan	Banswara	29.62	10.46	4.40	25.87	8.47	4.54	20.56	5.97	2.89	27.32	10.02	4.53
Rajasthan	Chittaurgarh	31.94	10.48	3.55	29.35	8.11	4.82	21.23	5.79	2.84	33.74	10.21	4.45
Rajasthan	Kota	23.68	7.00	4.00	26.10	11.45	4.78	19.93	7.33	2.76	25.11	9.11	5.88
Rajasthan	Baran	28.90	7.69	3.40	27.13	9.83	4.12	20.39	6.89	2.83	29.84	8.59	3.72
Rajasthan	Jhalawar	37.98	9.96	3.93	25.93	10.14	5.46	19.47	7.54	2.81	36.08	10.41	5.36
Rajasthan	Udaipur	29.95	11.80	6.21	27.22	8.51	5.75	20.09	5.60	2.84	30.81	11.12	8.62
Rajasthan	Pratapgarh	29.14	11.10	3.76	27.63	8.69	5.81	19.58	5.80	2.80	30.62	10.87	5.60
Sikkim	North District	23.54	8.31	2.74	13.65	7.88	2.73	12.51	4.85	2.69	20.38	9.41	2.50
Sikkim	West District	32.88	9.35	2.68	14.71	11.29	2.79	12.89	4.94	2.65	29.67	13.30	2.49

Sikkim	South District	25.39	7.53	2.75	13.42	8.14	2.75	13.13	4.74	2.68	20.32	9.04	2.53
Sikkim	East District	22.32	7.32	2.61	13.29	9.38	3.03	11.83	3.71	2.63	20.61	10.86	2.81
Tamil Nadu	Thiruvallur	14.21	6.10	4.55	15.06	5.73	3.15	12.41	7.46	2.87	16.56	5.29	4.77
Tamil Nadu	Chennai	14.37	5.42	4.00	13.06	5.01	3.09	11.03	3.91	2.86	.	.	.
Tamil Nadu	Kancheepuram	14.38	5.39	5.12	13.53	5.12	4.05	12.16	4.29	3.12	14.77	5.36	4.78
Tamil Nadu	Vellore	14.62	6.80	4.48	14.29	4.91	3.49	13.11	6.51	2.91	15.28	5.34	5.38
Tamil Nadu	Tiruvannamalai	16.22	5.34	3.92	14.14	5.14	3.10	12.31	4.91	2.92	17.30	5.07	3.95
Tamil Nadu	Viluppuram	14.04	6.65	4.33	13.96	4.99	3.07	12.84	4.73	2.91	14.57	6.04	4.44
Tamil Nadu	Salem	17.36	5.21	4.44	13.00	5.10	3.09	12.74	4.33	2.96	16.23	5.26	4.09
Tamil Nadu	Namakkal	13.98	5.27	4.00	13.60	5.12	3.62	12.31	4.33	2.91	14.83	5.35	4.88
Tamil Nadu	Erode	13.98	5.89	3.93	13.15	5.71	3.55	11.58	6.68	2.96	14.74	5.35	4.19
Tamil Nadu	The Nilgiris	14.78	5.31	3.90	13.29	5.02	3.16	11.45	4.13	2.92	15.60	5.38	4.24
Tamil Nadu	Dindigul	16.09	5.88	3.95	13.56	5.65	3.13	11.76	5.48	2.89	17.14	5.73	4.04
Tamil Nadu	Karur	13.77	5.99	3.88	13.85	5.65	3.07	12.60	5.29	2.88	14.09	5.83	4.05
Tamil Nadu	Tiruchirappalli	15.50	6.77	3.97	14.44	8.27	4.04	12.87	5.63	2.95	16.82	8.77	4.56
Tamil Nadu	Perambalur	17.17	6.81	3.89	13.34	5.17	3.68	12.84	4.79	2.89	15.94	6.37	4.51
Tamil Nadu	Ariyalur	16.30	5.41	4.60	13.98	5.16	3.14	12.94	4.96	2.89	16.22	5.17	4.63
Tamil Nadu	Cuddalore	17.24	5.23	4.27	12.99	6.39	3.17	13.45	4.28	2.88	15.51	6.33	4.67
Tamil Nadu	Nagapattinam	14.82	5.24	5.82	14.48	5.19	3.55	12.25	4.65	2.99	16.72	5.13	5.76
Tamil Nadu	Thiruvarur	16.17	5.34	3.91	14.56	6.47	3.15	13.59	4.72	2.89	16.49	6.13	3.97
Tamil Nadu	Thanjavur	15.90	5.89	3.85	14.56	5.64	3.15	12.67	7.00	2.89	17.48	5.38	4.15
Tamil Nadu	Pudukkottai	14.63	5.22	4.46	13.97	4.99	3.15	13.19	4.71	2.88	14.27	5.01	4.39
Tamil Nadu	Sivaganga	14.05	5.88	3.88	14.79	5.69	3.19	12.83	7.02	2.90	15.65	5.31	4.20
Tamil Nadu	Madurai	14.88	6.68	3.93	14.59	5.09	3.15	12.25	7.31	2.89	17.01	5.32	4.14
Tamil Nadu	Theni	15.05	6.15	3.98	13.65	5.82	3.17	12.49	4.44	2.91	15.57	6.60	4.26
Tamil Nadu	Virudhunagar	15.75	5.44	4.06	14.49	5.19	3.60	12.45	4.47	2.91	17.84	5.40	4.87
Tamil Nadu	Ramanathapuram	15.07	5.17	4.41	13.34	5.87	3.19	12.84	5.52	2.85	14.57	5.31	4.76
Tamil Nadu	Thoothukkudi	15.21	5.24	5.89	13.23	5.18	3.11	12.28	4.21	2.98	14.91	5.55	5.47
Tamil Nadu	Tirunelveli	18.10	6.09	5.27	13.83	5.84	4.09	13.33	4.55	2.89	17.64	6.54	7.13
Tamil Nadu	Kanniyakumari	14.95	5.24	4.51	13.37	4.96	3.99	12.14	3.93	3.07	15.50	5.45	4.38
Tamil Nadu	Dharmapuri	15.46	6.00	3.89	13.42	5.64	3.12	12.13	4.71	2.88	16.00	6.26	3.98
Tamil Nadu	Krishnagiri	17.73	6.50	4.95	14.82	5.67	3.14	12.66	6.00	2.90	19.72	6.02	5.00
Tamil Nadu	Coimbatore	15.21	5.88	3.90	13.55	5.09	3.08	11.91	4.94	2.85	16.98	5.52	4.31
Tamil Nadu	Tiruppur	17.30	5.48	4.54	14.46	5.71	3.11	13.27	4.22	2.94	17.90	6.02	4.28
Tripura	West Tripura	15.80	12.44	3.22	13.30	8.57	3.14	9.69	7.50	2.75	16.14	12.57	2.85
Tripura	South Tripura	16.01	10.74	2.89	14.03	6.88	2.98	10.21	6.36	2.69	16.55	9.67	2.60
Tripura	Dhalai	15.45	15.34	2.89	14.32	6.67	2.91	10.15	8.54	2.73	16.18	11.57	2.55
Tripura	North Tripura	18.03	17.90	2.79	14.90	8.23	3.25	10.21	6.25	2.70	19.70	16.03	2.81
Uttar Pradesh	Saharanpur	36.53	14.23	4.15	39.25	28.44	6.41	28.67	22.63	2.94	44.52	19.17	6.78
Uttar Pradesh	Muzaffarnagar	44.09	10.10	3.67	41.85	15.86	6.13	33.21	7.67	2.99	49.13	14.16	4.91
Uttar Pradesh	Bijnor	38.31	16.29	4.22	43.36	15.09	6.19	34.01	15.50	3.04	43.11	15.72	5.58
Uttar Pradesh	Moradabad	46.41	17.70	5.01	44.33	14.98	7.07	31.71	12.20	3.12	56.77	18.39	7.27
Uttar Pradesh	Rampur	42.40	11.33	3.78	36.56	23.57	7.72	34.28	25.69	3.04	39.18	13.92	5.97
Uttar Pradesh	Jyotiba Phule Nagar	42.23	12.46	4.31	42.06	19.68	9.24	31.50	12.77	3.01	47.89	16.56	8.00
Uttar Pradesh	Meerut	45.12	11.41	3.46	36.93	19.32	6.48	41.21	12.27	2.90	37.19	16.94	6.58
Uttar Pradesh	Baghpat	38.64	15.37	3.90	45.64	15.67	7.18	37.40	13.22	3.05	42.96	15.96	5.65
Uttar Pradesh	Ghaziabad	43.05	16.33	3.81	33.12	24.81	7.89	28.90	22.94	3.14	42.26	18.65	6.01
Uttar Pradesh	Gautam Buddha Nagar	41.96	15.91	4.91	37.71	21.92	6.91	30.48	11.08	3.07	45.13	22.82	7.30
Uttar Pradesh	Bulandshahr	44.12	14.65	4.69	36.72	15.53	6.84	36.98	8.47	3.05	39.41	17.27	6.62
Uttar Pradesh	Aligarh	50.65	16.87	3.99	44.49	26.81	6.98	39.22	19.45	3.19	52.85	22.82	5.35
Uttar Pradesh	Mahamaya Nagar	46.83	17.73	4.25	39.99	20.30	7.64	31.46	9.10	3.13	47.96	22.58	5.96
Uttar Pradesh	Mathura	59.05	11.08	4.21	39.63	23.05	7.44	34.80	8.14	3.10	56.49	19.82	5.87
Uttar Pradesh	Agra	45.68	12.96	5.07	36.90	27.33	6.85	31.87	19.76	3.01	45.40	19.32	7.53
Uttar Pradesh	Firozabad	46.41	10.52	4.60	44.75	22.86	10.07	40.22	16.53	3.39	48.35	15.06	6.76
Uttar Pradesh	Mainpuri	51.41	13.08	5.48	41.71	20.00	8.70	40.59	9.21	3.05	47.21	18.07	9.17

Uttar Pradesh	Budaun	53.66	11.96	4.67	45.69	16.14	8.03	35.88	11.18	3.05	56.05	13.94	7.33
Uttar Pradesh	Bareilly	39.63	16.46	4.81	35.80	21.58	7.70	34.33	11.84	3.26	37.18	23.60	6.74
Uttar Pradesh	Pilibhit	41.57	15.24	4.91	39.37	16.09	9.38	34.49	11.84	3.19	40.01	16.39	7.19
Uttar Pradesh	Shahjahanpur	63.43	12.56	5.32	37.52	26.58	6.07	36.72	8.80	3.04	53.80	21.91	6.58
Uttar Pradesh	Kheri	40.33	10.32	4.22	45.05	23.09	7.57	33.58	13.16	3.04	45.63	16.39	6.34
Uttar Pradesh	Sitapur	54.29	15.09	5.34	47.45	25.64	13.08	34.43	12.23	3.04	60.22	21.67	12.44
Uttar Pradesh	Hardoi	59.10	12.97	6.79	42.84	15.79	9.84	40.09	9.62	3.06	55.54	14.86	11.45
Uttar Pradesh	Unnao	46.33	12.06	3.88	37.08	20.25	6.32	32.57	9.45	3.04	43.17	17.31	4.90
Uttar Pradesh	Lucknow	41.00	12.73	4.18	38.47	17.63	6.12	35.26	7.64	3.06	39.60	18.37	5.63
Uttar Pradesh	Rae Bareli	45.64	13.93	3.90	37.81	22.81	7.24	34.12	13.31	3.08	42.23	19.06	5.64
Uttar Pradesh	Farrukhabad	56.42	19.30	4.18	40.86	29.52	8.24	44.88	27.76	3.04	46.95	23.57	7.11
Uttar Pradesh	Kannauj	46.17	19.84	4.26	40.71	22.29	8.15	36.81	17.57	3.07	44.90	22.06	7.19
Uttar Pradesh	Etawah	44.27	12.84	4.29	34.09	18.32	7.06	36.51	8.39	3.11	35.40	17.59	5.55
Uttar Pradesh	Auraiya	41.18	11.21	4.40	36.53	16.18	7.15	33.95	9.82	3.05	38.47	14.04	6.36
Uttar Pradesh	Kanpur Dehat	51.90	10.10	4.42	33.57	20.63	8.72	32.60	10.38	3.05	42.90	15.48	7.71
Uttar Pradesh	Kanpur Nagar	40.33	12.47	4.16	32.82	13.14	6.01	32.47	8.80	3.09	34.99	13.79	5.07
Uttar Pradesh	Jalaun	48.63	14.44	3.98	42.01	16.19	7.81	33.54	10.02	3.05	51.00	16.44	5.85
Uttar Pradesh	Jhansi	44.14	9.88	4.12	35.67	22.62	7.39	32.35	9.27	3.11	42.40	18.42	6.45
Uttar Pradesh	Lalitpur	42.47	14.16	5.05	38.66	18.30	11.43	34.25	10.28	3.08	40.56	17.64	10.79
Uttar Pradesh	Hamirpur	40.36	14.64	3.91	38.93	15.46	6.53	32.98	9.76	3.05	41.28	16.41	5.45
Uttar Pradesh	Mahoba	46.58	12.79	4.44	34.21	19.56	6.44	34.46	9.52	3.07	38.44	17.86	5.75
Uttar Pradesh	Banda	46.46	13.43	4.54	35.83	19.01	8.46	33.41	9.64	3.14	42.25	17.58	6.69
Uttar Pradesh	Chitrakoot	43.78	17.94	4.23	39.54	18.53	7.51	35.39	23.24	3.05	42.18	16.72	6.39
Uttar Pradesh	Fatehpur	38.40	12.86	4.56	39.79	14.16	7.46	33.12	12.88	3.03	39.32	13.00	6.65
Uttar Pradesh	Pratapgarh	47.29	14.86	4.84	33.72	17.76	7.99	33.74	11.32	3.09	39.22	17.20	7.28
Uttar Pradesh	Kaushambi	59.75	19.40	5.50	41.38	18.85	7.61	34.00	10.03	3.06	56.65	21.98	7.83
Uttar Pradesh	Allahabad	41.47	10.72	4.85	35.95	23.91	8.53	33.48	11.85	3.14	37.92	17.95	7.28
Uttar Pradesh	Bara Banki	42.53	16.82	4.38	32.78	23.17	6.99	32.49	10.31	3.09	35.60	22.53	6.13
Uttar Pradesh	Faizabad	46.63	15.88	4.23	41.67	19.27	6.85	32.91	12.41	3.04	48.60	18.32	6.05
Uttar Pradesh	Ambedkar Nagar	49.35	11.48	4.19	40.64	13.91	6.93	37.00	12.03	3.07	47.14	12.07	5.83
Uttar Pradesh	Sultanpur	49.98	14.30	4.90	40.96	22.10	8.92	36.19	10.71	3.07	48.03	19.82	8.18
Uttar Pradesh	Bahraich	54.93	11.91	4.59	45.76	19.19	5.81	35.49	12.08	3.03	56.55	15.94	5.44
Uttar Pradesh	Shrawasti	44.25	20.73	5.70	46.55	20.38	11.35	34.73	13.91	3.07	49.78	22.19	10.65
Uttar Pradesh	Balrampur	42.61	16.10	3.65	44.27	18.63	10.81	33.62	10.12	3.06	46.45	18.89	7.57
Uttar Pradesh	Gonda	69.00	10.93	4.73	45.19	16.98	10.63	42.40	13.27	3.05	61.23	13.37	9.32
Uttar Pradesh	Siddharth Nagar	51.40	12.74	4.07	38.76	18.57	12.57	37.41	10.47	3.04	45.33	15.92	9.67
Uttar Pradesh	Basti	40.23	13.12	5.23	37.36	15.57	9.10	32.81	10.63	3.08	38.72	14.57	8.59
Uttar Pradesh	Sant Kabir Nagar	43.59	14.27	5.24	37.70	15.27	8.18	35.91	9.75	3.06	39.81	15.51	7.99
Uttar Pradesh	Mahrajganj	56.71	13.22	3.67	41.19	13.03	8.35	34.33	10.20	3.09	54.26	13.06	6.08
Uttar Pradesh	Gorakhpur	42.89	14.64	4.81	38.05	16.60	7.77	34.30	9.87	3.19	40.81	17.09	5.87
Uttar Pradesh	Kushinagar	45.04	15.48	5.01	35.29	16.48	6.61	32.35	10.54	3.02	40.55	16.88	6.51
Uttar Pradesh	Deoria	42.68	13.25	4.30	36.66	13.32	8.68	31.86	9.71	3.04	40.35	13.57	7.21
Uttar Pradesh	Azamgarh	40.15	12.97	3.80	39.77	14.55	6.26	37.75	10.07	3.06	38.29	14.38	4.79
Uttar Pradesh	Mau	48.45	12.65	4.83	35.82	19.17	8.65	35.44	15.84	3.09	41.45	14.96	7.38
Uttar Pradesh	Ballia	38.25	13.69	4.79	37.08	15.80	6.90	31.70	9.76	3.09	37.61	15.76	6.34
Uttar Pradesh	Jaunpur	46.69	16.11	3.74	38.40	22.27	6.18	38.58	17.02	3.06	41.52	19.44	4.66
Uttar Pradesh	Ghazipur	45.19	18.51	4.88	37.29	14.66	7.68	35.68	10.17	3.12	40.90	18.13	6.38
Uttar Pradesh	Chandauli	38.00	13.33	3.66	35.92	16.93	5.95	30.52	9.10	3.07	37.32	16.29	4.59
Uttar Pradesh	Varanasi	38.88	9.15	3.44	37.70	15.13	6.68	26.48	8.37	3.13	46.27	12.59	4.48
Uttar Pradesh	Sant Ravidas Nagar (Bhadohi)	49.88	15.00	4.59	38.27	16.06	6.51	33.23	9.19	3.02	47.36	17.13	6.00
Uttar Pradesh	Mirzapur	53.32	15.29	5.39	42.14	15.93	7.65	32.19	18.74	3.04	54.25	14.39	7.86
Uttar Pradesh	Sonbhadra	47.72	10.85	3.78	37.65	12.21	8.61	33.13	9.82	3.08	44.86	11.08	6.54
Uttar Pradesh	Etah	50.41	13.43	3.76	37.83	20.17	6.91	32.91	9.86	3.04	47.74	18.44	5.27
Uttar Pradesh	Kanshiram Nagar	55.83	20.39	3.63	40.69	24.11	8.26	35.12	15.95	3.04	54.08	24.11	6.26
Uttarakhand	Uttarkashi	27.49	19.87	2.52	26.09	6.77	3.29	29.57	8.76	2.63	26.27	14.01	2.64

Uttarakhand	Chamoli	23.96	18.37	2.52	26.74	7.34	3.64	30.00	7.89	2.66	23.83	14.65	3.03
Uttarakhand	Rudraprayag	25.02	12.51	2.56	23.65	6.67	3.26	29.51	8.63	2.65	21.08	9.88	2.68
Uttarakhand	Tehri Garhwal	24.77	14.44	2.87	24.29	7.34	3.59	28.98	10.59	2.73	22.28	10.78	2.95
Uttarakhand	Dehradun	28.22	10.50	2.45	24.91	6.96	3.48	30.53	7.11	2.61	23.91	9.22	2.98
Uttarakhand	Garhwal	23.57	11.43	2.54	24.59	6.78	3.25	28.76	7.91	2.65	21.72	9.13	2.71
Uttarakhand	Pithoragarh	30.38	15.25	2.96	25.71	7.69	3.33	28.42	7.96	2.66	29.44	12.84	3.09
Uttarakhand	Bageshwar	22.21	9.65	2.54	27.18	7.38	3.24	29.96	8.62	2.68	22.79	8.51	2.60
Uttarakhand	Almora	27.79	11.00	2.55	25.10	6.61	3.24	33.55	8.12	2.65	22.93	8.83	2.62
Uttarakhand	Champawat	28.47	13.44	2.53	26.08	7.33	3.63	32.12	9.94	2.65	25.59	10.34	2.99
Uttarakhand	Nainital	20.67	13.68	2.72	29.75	6.43	3.83	30.30	12.23	2.70	22.67	8.90	3.27
Uttarakhand	Udham Singh Nagar	26.66	10.37	2.95	27.57	9.78	3.66	33.14	10.63	2.66	23.71	10.08	3.63
Uttarakhand	Hardwar	31.16	11.16	2.53	27.28	13.37	3.96	34.45	13.75	2.64	26.87	12.00	3.45
West Bengal	Darjiling	26.86	5.79	2.77	16.82	4.98	2.23	12.15	4.54	2.34	25.77	5.55	2.74
West Bengal	Jalpaiguri	27.40	5.74	3.19	15.58	4.88	2.54	11.70	4.77	2.38	23.72	5.27	3.45
West Bengal	Koch Bihar	31.05	7.36	2.74	16.44	6.17	2.19	11.91	4.76	2.39	27.85	7.44	2.58
West Bengal	Uttar Dinajpur	35.14	6.60	2.92	16.27	4.60	2.16	11.71	4.70	2.40	30.37	5.70	2.79
West Bengal	Dakshin Dinajpur	27.45	7.39	2.76	18.92	4.98	2.20	11.88	4.94	2.37	29.89	6.46	2.67
West Bengal	Maldah	28.45	6.81	3.93	16.42	4.74	2.50	13.70	6.08	2.34	23.61	5.46	4.09
West Bengal	Murshidabad	29.30	6.12	3.05	16.27	4.75	2.18	12.10	4.39	2.36	26.35	5.55	2.86
West Bengal	Birbhum	28.20	6.43	2.81	16.45	5.63	2.23	11.86	6.30	2.38	25.68	5.69	2.59
West Bengal	Barddhaman	27.49	5.65	2.77	15.41	7.03	2.18	11.12	5.36	2.35	25.72	6.87	2.70
West Bengal	Nadia	25.92	7.25	2.74	16.43	5.61	2.22	11.66	5.79	2.34	24.66	6.53	2.62
West Bengal	North Twenty Four Parganas	27.54	6.59	2.79	16.11	5.05	2.21	12.01	4.39	2.37	25.01	6.10	2.66
West Bengal	Hugli	26.02	5.86	2.78	16.67	5.72	2.23	11.97	4.43	2.37	24.72	6.14	2.74
West Bengal	Bankura	32.12	5.81	2.79	16.97	4.87	2.46	12.71	4.70	2.36	28.28	5.31	2.98
West Bengal	Puruliya	29.12	5.54	2.68	17.53	5.26	2.13	12.39	4.55	2.36	27.14	5.43	2.50
West Bengal	Haora	28.28	6.45	2.75	16.20	4.93	2.18	12.39	4.29	2.36	25.61	6.05	2.67
West Bengal	Kolkata	26.34	5.98	2.78	16.39	5.64	2.26	12.05	5.10	2.36	.	.	.
West Bengal	South Twenty Four Parganas	29.69	5.64	3.05	15.94	4.76	2.77	12.04	4.60	2.35	25.96	5.19	3.74
West Bengal	Paschim Medinipur	29.07	6.41	2.76	16.49	4.89	2.25	11.86	4.76	2.39	26.95	5.85	2.61
West Bengal	Purba Medinipur	25.14	6.42	2.81	16.77	6.17	2.22	11.85	6.29	2.38	23.66	6.20	2.59
Telangana	Adilabad	22.98	7.46	2.78	21.46	7.63	2.07	14.34	6.16	2.69	27.24	7.92	2.06
Telangana	Nizamabad	26.23	7.44	3.07	23.07	7.80	2.07	16.66	6.17	2.77	29.11	7.83	1.95
Telangana	Karimnagar	25.20	6.71	2.82	21.82	7.94	2.09	15.07	7.57	2.68	29.27	6.83	2.05
Telangana	Medak	24.25	8.34	2.78	22.49	9.69	2.06	14.32	13.06	2.68	31.01	7.87	1.98
Telangana	Hyderabad	20.75	6.56	2.74	19.43	6.67	2.33	12.51	4.59	2.72	.	.	.
Telangana	Rangareddy	24.17	6.61	2.70	19.61	7.92	2.01	12.84	6.59	2.67	29.84	7.13	2.05
Telangana	Mahbubnagar	23.49	9.37	2.70	21.05	8.38	2.00	13.98	6.38	2.70	26.72	9.48	1.94
Telangana	Nalgonda	22.39	6.55	2.81	20.94	6.99	2.09	13.81	6.22	2.71	25.84	6.59	1.99
Telangana	Warangal	21.90	7.70	2.76	21.12	6.99	2.08	15.37	8.01	2.74	24.51	6.82	2.03
Telangana	Khammam	22.29	7.60	3.13	21.63	6.88	2.08	13.81	7.96	2.71	27.29	6.76	2.24

Figure S1. Index map of India labeled with state names

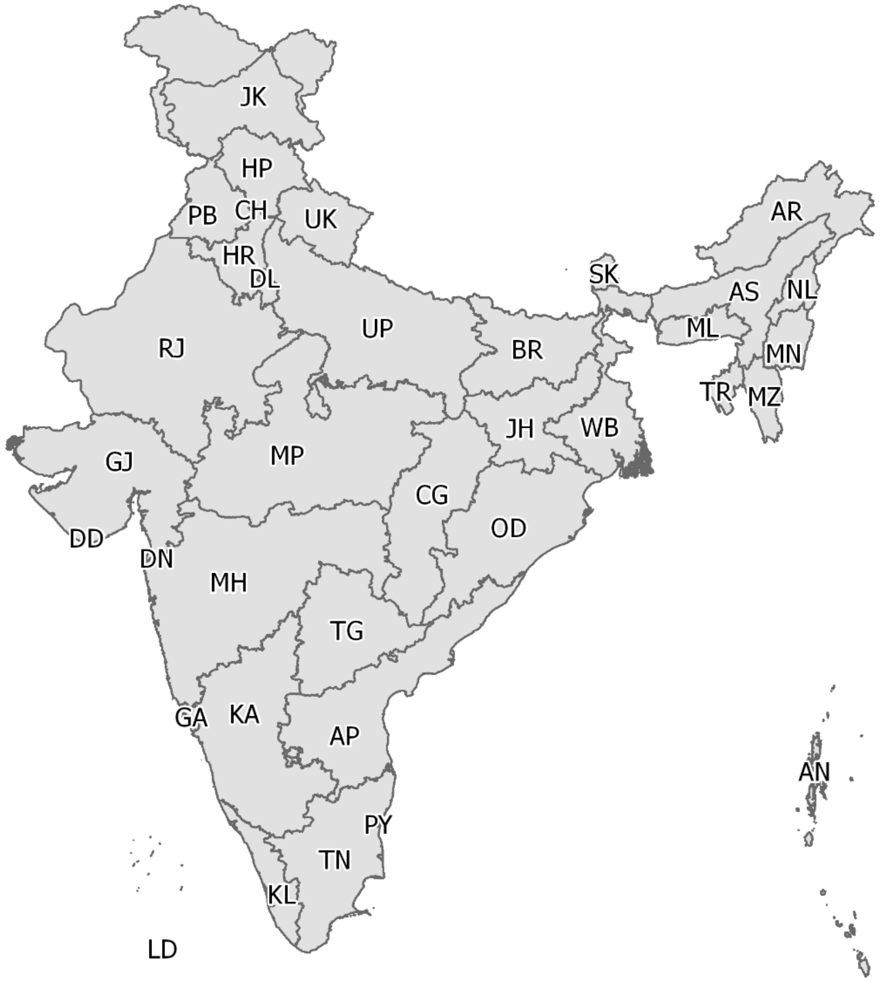

State	Code	State	Code	State	Code	State	Code
Andaman and Nicobar	AN	Delhi	DL	Lakshadweep	LD	Punjab	PB
Andhra Pradesh	AP	Goa	GA	Madhya Pradesh	MP	Rajasthan	RJ
Arunachal Pradesh	AR	Gujarat	GJ	Maharashtra	MH	Sikkim	SK
Assam	AS	Haryana	HR	Manipur	MN	Tamil Nadu	TN
Bihar	BR	Himachal Pradesh	HP	Meghalaya	ML	Telangana	TG
Chandigarh	CH	Jammu and Kashmir	JK	Mizoram	MZ	Tripura	TR
Chhattisgarh	CG	Jharkhand	JH	Nagaland	NL	Uttar Pradesh	UP
Dadra and Nagar Haveli	DN	Karnataka	KA	Odisha	OD	Uttarakhand	UK
Daman and Diu	DD	Kerala	KL	Puducherry	PY	West Bengal	WB

Figure S2A. Spatial clustering of districts with high probability of neonatal mortality detected using the Cluster and Outlier Analysis (Anselin Local Moran's I)

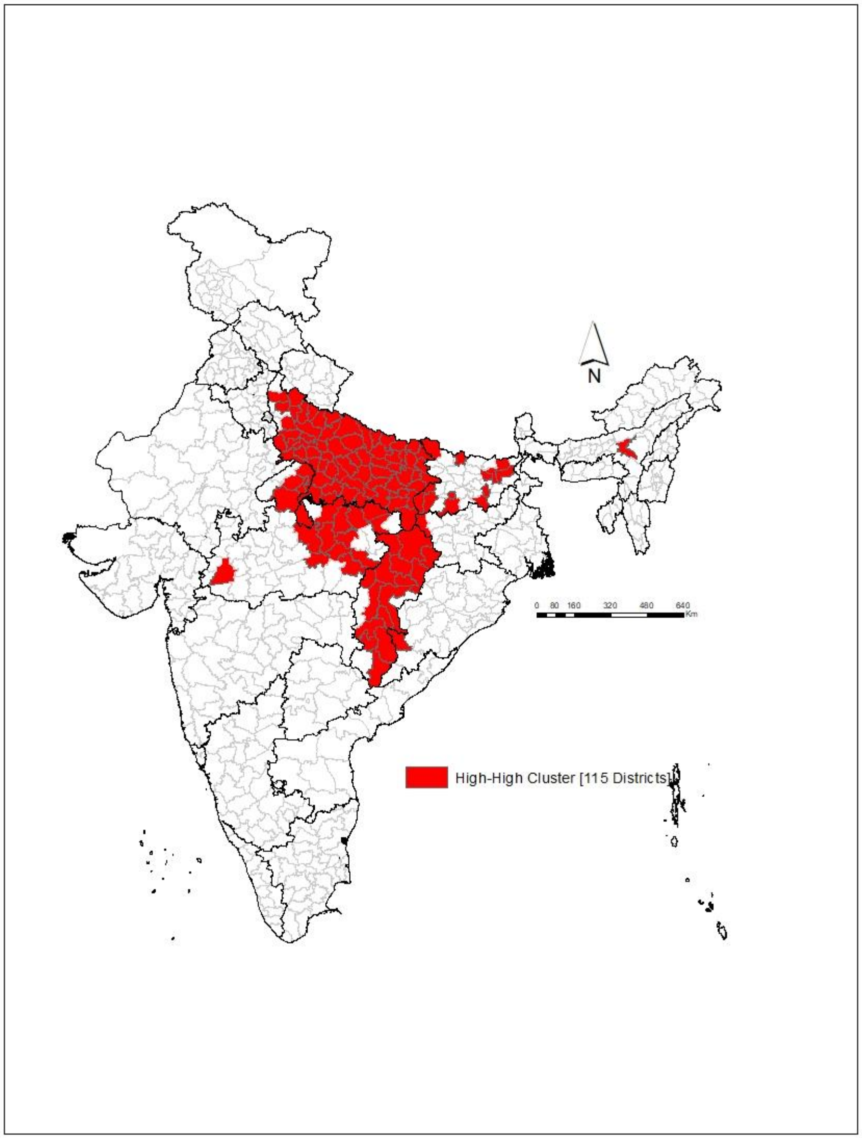

Figure S2B. Spatial clustering of districts with high probability of post-neonatal mortality detected using the Cluster and Outlier Analysis (Anselin Local Moran's I)

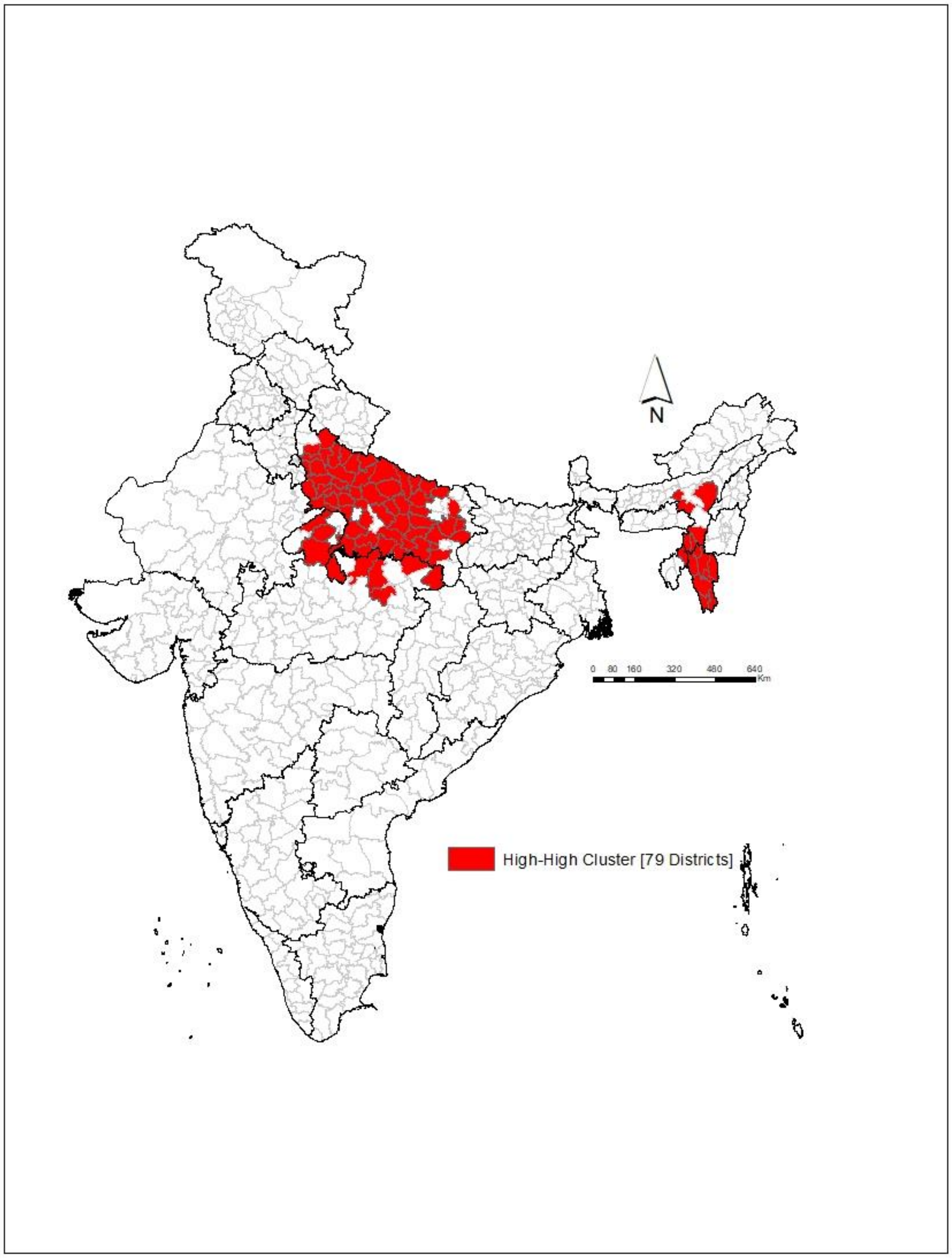

Figure S2C. Spatial clustering of districts with high probability of post-neonatal mortality detected using the Cluster and Outlier Analysis (Anselin Local Moran's I)

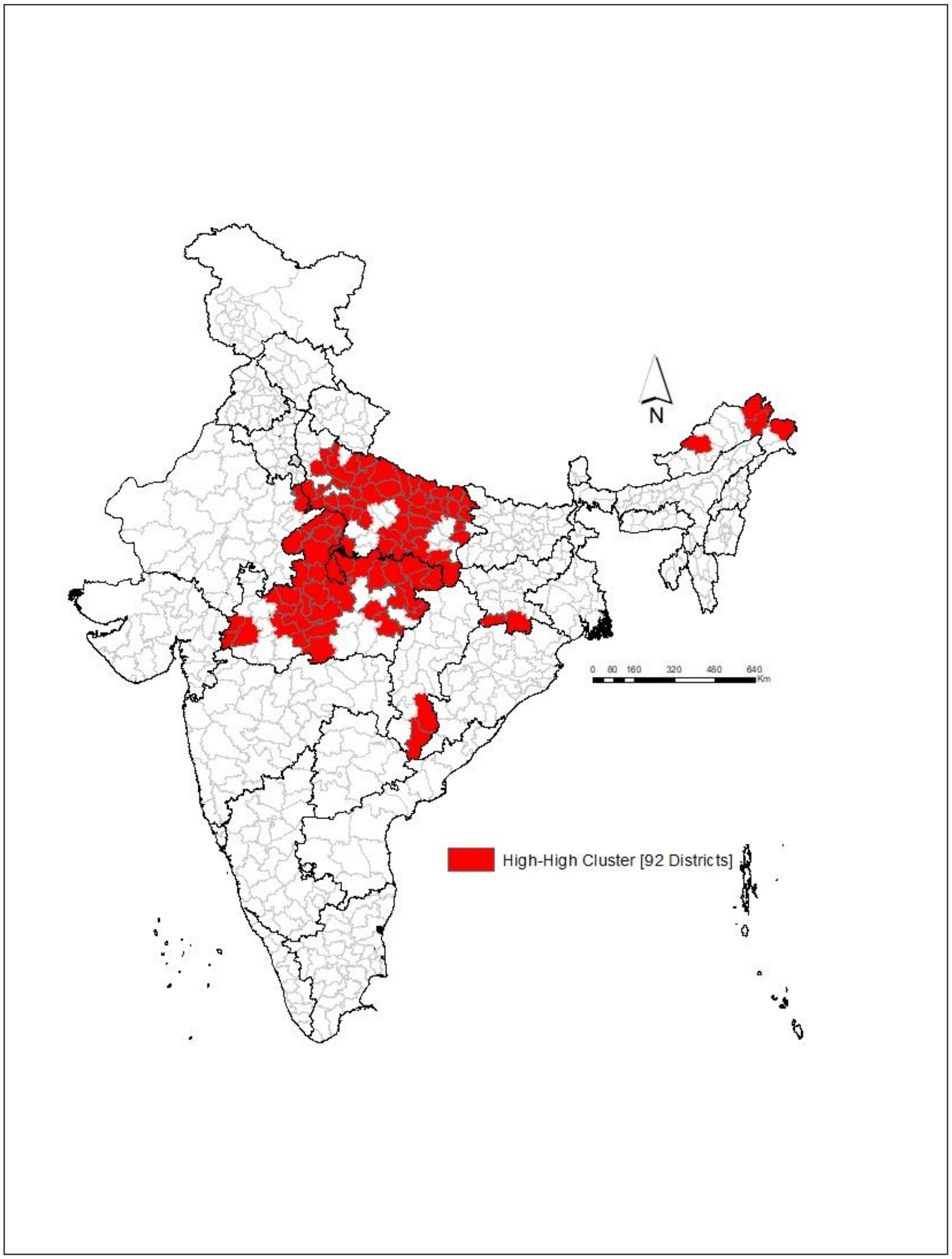

Supplement: Online Supplementary Document [file jogh-10-020405-s001.pdf]
